# Discovery of potent small-molecule inhibitors of lipoprotein(a) formation

**DOI:** 10.1038/s41586-024-07387-z

**Published:** 2024-05-08

**Authors:** Nuria Diaz, Carlos Perez, Ana Maria Escribano, Gema Sanz, Julian Priego, Celia Lafuente, Mario Barberis, Luis Calle, Juan Felix Espinosa, Birgit T. Priest, Hong Y. Zhang, Amanda K. Nosie, Joseph V. Haas, Ellen Cannady, Anthony Borel, Albert E. Schultze, J. Michael Sauder, Jörg Hendle, Ken Weichert, Stephen J. Nicholls, Laura F. Michael

**Affiliations:** 1 Lilly Research Laboratories, Alcobendas, Spain; 2 Lilly Research Laboratories, Indianapolis, IN USA; 3 Lilly Research Laboratories, San Diego, CA USA; 4 Victorian Heart Institute, Monash University, Clayton, Victoria Australia

**Keywords:** Pharmacology, X-ray crystallography, Dyslipidaemias

## Abstract

Lipoprotein(a) (Lp(a)), an independent, causal cardiovascular risk factor, is a lipoprotein particle that is formed by the interaction of a low-density lipoprotein (LDL) particle and apolipoprotein(a) (apo(a))^1,2^. Apo(a) first binds to lysine residues of apolipoprotein B-100 (apoB-100) on LDL through the Kringle IV (K_IV_) 7 and 8 domains, before a disulfide bond forms between apo(a) and apoB-100 to create Lp(a) (refs. ^3–7^). Here we show that the first step of Lp(a) formation can be inhibited through small-molecule interactions with apo(a) K_IV_7–8. We identify compounds that bind to apo(a) K_IV_7–8, and, through chemical optimization and further application of multivalency, we create compounds with subnanomolar potency that inhibit the formation of Lp(a). Oral doses of prototype compounds and a potent, multivalent disruptor, LY3473329 (muvalaplin), reduced the levels of Lp(a) in transgenic mice and in cynomolgus monkeys. Although multivalent molecules bind to the Kringle domains of rat plasminogen and reduce plasmin activity, species-selective differences in plasminogen sequences suggest that inhibitor molecules will reduce the levels of Lp(a), but not those of plasminogen, in humans. These data support the clinical development of LY3473329—which is already in phase 2 studies—as a potent and specific orally administered agent for reducing the levels of Lp(a).

## Abstract (web summary)

Biochemical screening and optimization identify small molecules that inhibit the formation of lipoprotein(a), and these inhibitors reduce the levels of Lp(a) in several animal models, suggesting that they could provide a therapeutic option in humans.

## Main

Lp(a) is a genetically determined, independent cardiovascular risk factor that cannot be modified through lifestyle changes, and no therapeutic agents are available that specifically reduce the levels of Lp(a) without having other effects. High serum levels of Lp(a) (more than 50 mg dl^−1^ or 125 nmol l^−1^), which are found in around 20% of the population, confer an increase in risk of more than 1.6-fold for a first cardiovascular event^8^ and more than 1.42-fold for a second event^9^, with risk increasing in direct proportion to serum Lp(a) levels^10^. Circulating levels of Lp(a) are determined mainly by the hypervariable number of Kringle IV subtype 2 (K_IV_2) domains, as well as by single-nucleotide polymorphisms (SNP) of the *LPA* gene^11^. Epidemiological studies have determined that Lp(a) is an independent cardiovascular risk factor, and because Lp(a) levels are determined by gene structure, Mendelian randomization studies have shown that Lp(a) has a role in causing both atherosclerosis and calcific aortic valve disease^12–14^.

Lp(a) is a lipoprotein particle that is formed by a two-step process involving interactions between a LDL particle and another protein, apo(a). In the first step, apo(a) binds non-covalently to lysine-rich regions in apoB-100 that reside on LDL particles through structural elements of the unique K_IV_5–8, the lysine-binding sites of which are cognate to the lysine-coordinating residues of apoB. In the second step, a disulfide bond is formed between the unpaired Cys67 residue of K_IV_9 of apo(a) (refs. ^5,7^) and Cys3734 of apoB-100 (refs. ^3,6^), which covalently links the two proteins.

**Extended Data Fig. 1:**
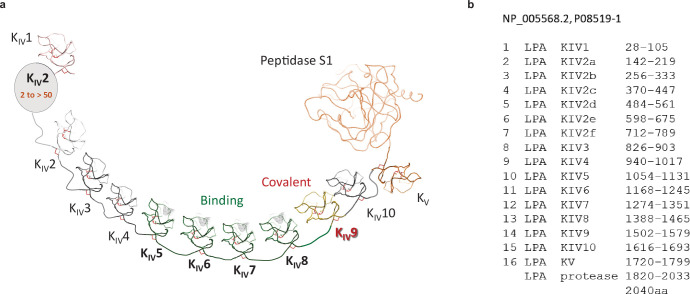
Schematic of apolipoprotein(a) domains. **a**, The type IV Kringle domains of apo(a) are shown, ending with a single type V Kringle domain and C-terminal peptidase domain. **b**, Numbering of domains based on the Uniprot (P08519-1) and GenBank RefSeq (NP_005568.2) amino acid residue numbers are provided in the table, with the Kringle domains defined by their first and last cysteine residues that form a disulfide bond.

Apo(a) is encoded by the *LPA* gene, which is present only in the genomes of humans and some non-human primates^15^. The *LPA* gene arose from the plasminogen (*PLG*) gene by a gene duplication event late in evolution, in which the plasminogen Kringle domains I, II and III were lost, and the K_IV_ domain expanded and diverged to 10 subtypes (K_IV_1–10) (Extended Data Fig. 1); thus, the K_IV_ domains of apo(a) share 70–85% sequence identity with plasminogen Kringle domains.

At present, there are no therapeutics available in clinical practice that specifically reduce the levels of Lp(a) by itself. Pelacarsen, an antisense oligonucleotide that targets *LPA*, and olpasiran, an *LPA* small interfering RNA, are both injectable therapeutics that are currently in phase 3 clinical studies. These interventions have shown a robust Lp(a)-lowering effect, with good tolerability^16,17^. Results from ongoing phase 3 cardiovascular outcome trials, such as HORIZON and OCEAN(a), will provide much-needed evidence about the therapeutic utility of Lp(a)-lowering treatments^18,19^. So far, no small molecule that specifically targets apo(a) has been studied in humans; however, the plasminogen inhibitor tranexamic acid was found to reduce the levels of Lp(a) in a small clinical study^20^. The goal of our research was to develop a potent and selective orally delivered small-molecule therapeutic to inhibit the formation of Lp(a) by blocking the first interaction between apo(a) and apoB to reduce the circulating levels of Lp(a). A potent and selective small-molecule Lp(a)-reducing therapeutic could provide a way to decrease the number of primary and/or secondary major adverse cardiovascular events in patients who are at risk of cardiovascular disease because of their high levels of Lp(a).

Our strategy centred on identifying molecules that bind apo(a) K_IV_7–8 to inhibit the initial interaction between apo(a) and apoB. This approach is founded in knowledge obtained from three sources: (1) in vitro studies of recombinant apo(a) mutant proteins, which show that apo(a) binds non-covalently to lysine-rich regions in apoB-100 through the K_IV_6–8 domains; (2) analyses of the K_IV_8 domain SNP G17R in African populations, which is associated with significantly lower Lp(a) plasma concentrations than would be predicted by apo(a) K_IV_2 repeat polymorphisms; and (3) evidence that a G to A substitution at the +1 donor splice site of the K_IV_8 intron in Caucasian individuals is associated with a congenital deficiency of Lp(a) in the plasma^21–23^. Together, these findings created the hypothesis that blocking the K_IV_7–8 lysine-binding domains of apo(a) might prevent the formation of Lp(a). As a proof of principle, the in vitro assembly of Lp(a) is inhibited by ε-aminocaproic acid and tranexamic acid, which bind to the lysine-binding domain of apo(a) K_IV_7, despite their low binding affinities for K_IV_7 as measured by the equilibrium dissociation constant (*K*_d_) (230 µM and 63 µM, respectively)^24^. Similarly, low-affinity but selective K_IV_7 and K_IV_10 small-molecule binders have been described that reduce the formation of Lp(a) in vitro with micromolar potency^25^.

**Fig. 1:**
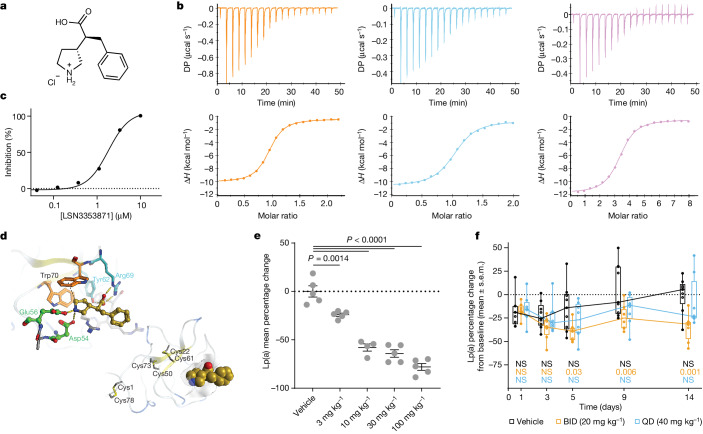
Identification of a small-molecule apo(a) binder and inhibitor of Lp(a) formation. **a**, Chemical structure of LSN3353871. **b**, ITC of LSN3353871 interacting with the apo(a) domains K_IV_8 (orange, one experiment), K_IV_7–8 (blue, two experiments) and K_IV_5–8 (violet, two experiments). DP, power differential; Δ*H*, enthalpy change. **c**, Average data for the inhibition of in vitro Lp(a) assembly by LSN3353871 (43 independent experiments; compound concentration 0.03–10 µM). **d**, Left, crystal structure of the K_IV_8–LSN3353871 complex (Protein Data Bank (PDB) ID: 8TCE), with the amino acids involved in ligand binding labelled. Ligand hydrogen bonds to D54, E56, Y62 and R69 are shown as yellow rods. The weak hydrogen bond between the pyrrolidine ring and W70 is shown as a dotted line. Right, the ligand is also shown in the same orientation as a space-filling model in a ribbon structure of the whole Kringle domain with disulfide bonds labelled. **e**, Percentage change in the steady-state levels of Lp(a) in *LPA* × *ApoB100* transgenic mice after five days of oral dosing. Data are from one experiment with *n* = 5 per group except *n* = 4 for the 10 mg kg^−1^ group, and are shown as mean ± s.e.m. with individuals plotted as circles. Two-sided *P* values were calculated using one-way ANOVA with Dunnett’s comparison to vehicle. **f**, Percentage change from baseline in the steady-state levels of Lp(a) in female cynomolgus monkeys during 14 days of oral dosing (*n* = 8 per group except *n* = 9 for the vehicle group) in a single experiment. Intersecting lines between box plots indicate median values, box limits indicate the 25th and 75th percentile and whiskers indicate the smallest and largest value. Two-sided *P* values are from a repeated measures ANOVA with Bonferroni comparisons to vehicle at each time point. The null hypothesis was rejected at *P* < 0.05. BID, twice daily; QD, once daily; NS, not significant. Source Data

**Extended Data Fig. 2:**
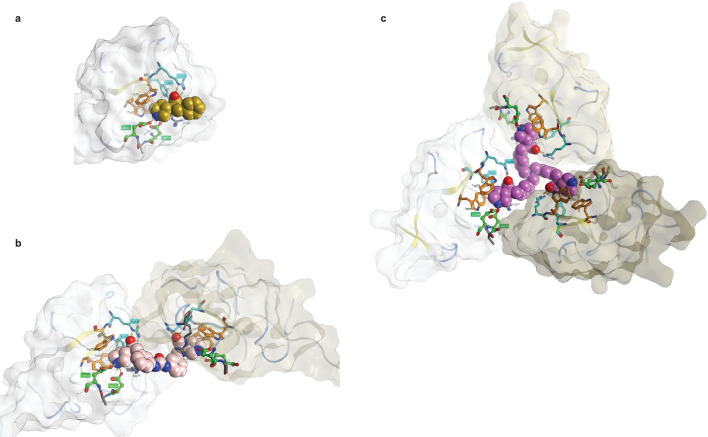
Co-crystal structures of apo(a) K_IV_ domains with small-molecule inhibitors. **a**, Apo(a) K_IV_8 in complex with LSN3353871 (PDB ID: 8TCE). Amino acids involved in ligand binding are labelled and the ligand hydrogen bonds to D54, E56, Y62 and R69 are shown as yellow rods. **b**, Apo(a) K_IV_7 in complex with LSN3441732 (PDB ID: 8V9B). This bivalent compound binds two Kringle domains simultaneously while conserving all the interactions observed in monomeric binding. **c**, Apo(a) K_IV_8 in complex with LY3473329 (PDB ID: 8V8Z), also conserving the same interactions.

Biochemical and biophysical compound screens using purified recombinant apo(a) K_IV_7–8 protein identified interacting small molecules. Optimization of the initial binding molecules led to the development and discovery of LSN3353871, which binds to K_IV_8, K_IV_7–8 and K_IV_5–8 with *K*_d_ values of 756 nM, 605 nM and 423 nM, respectively, as determined by isothermal calorimetry (ITC) (Fig. 1a,b). By contrast, LSN3353871 does not bind to K_IV_2 (Extended Data Table 1). In an in vitro Lp(a) assembly assay, LSN3353871 disrupted the formation of Lp(a) with a half-maximal inhibitory concentration (IC_50_) of 1.69 µM (*n* = 43) (Fig. 1c). Crystal structure analysis showed that LSN3353871 interacts with the K_IV_8 amino acid residues Glu56, Asp54, Tyr62 and Arg69, illustrating the specificity and binding modality of the molecule (Fig. 1d and Extended Data Fig. 2a). Oral administration of LSN3353871 to mice expressing human Lp(a) resulted in dose-dependent increases in the circulating levels of LSN3353871 and dose-dependent decreases of up to 78% in the levels of Lp(a), with a median effective dose (ED_50_) of 14 mg kg^−1^ twice daily (BID) within five days of dosing. In cynomolgus monkeys, administration of LSN3353871 (20 mg kg^−1^ BID for two weeks) resulted in a decrease of up to 40% in the levels of Lp(a) (Extended Data Table 2 and Fig. 1e,f). The difference in the magnitude of effect between these two models could be explained by differences in the expression levels and metabolism of Lp(a), because the mouse genome does not inherently contain the *LPA* gene. These data provide a proof of concept that a small molecule that binds to the lysine-binding domains of apo(a) can inhibit the formation of Lp(a) in vivo.

**Fig. 2:**
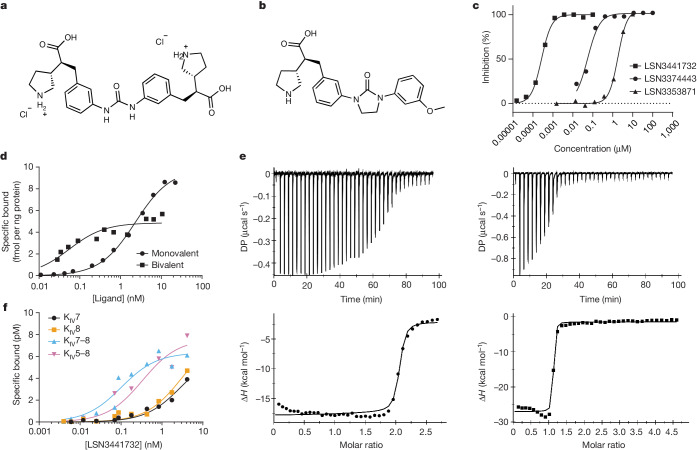
Mechanistic interactions with apo(a) K_IV_ domains and discovery of multivalency. **a**, Chemical structure of the dimeric molecule LSN3441732. **b**, Chemical structure of the optimized monovalent molecule LSN3374443. **c**, Average inhibition of in vitro Lp(a) assembly by LSN3353871 (triangles; 43 independent experiments), LSN3374443 (circles; 3 independent experiments) and LSN3441732 (squares; 8 independent experiments). **d**, Representative data from direct radioligand binding of ^3^H-LSN3374443 (circles; three experiments) and ^3^H-LSN3441732 (squares; one experiment) to full-length recombinant 16K apo(a). **e**, Representative data from ITC of LSN3374443 (circles; two experiments) and LSN3441732 (squares; three experiments) with apo(a) K_IV_5–8. **f**, Plot of direct binding of ^3^H-LSN3441732 to K_IV_7 (black; two experiments), K_IV_8 (orange; two experiments), K_IV_7–8 (blue; two experiments) and K_IV_5–8 (violet; one experiment); data are representative.

The linkage of two molecules that contain the crucial LSN3353871 K_IV_ domain interaction motif created a dimeric molecule, LSN3441732 (Fig. 2a). This dimeric compound inhibits the formation of Lp(a) particles in vitro with an IC_50_ value of 0.18 nM (*n* = 8), representing an increase in potency of three orders of magnitude compared with the parent monomeric molecule, LSN3353871 (IC_50_ = 1.69 µM; *n* = 43) and two orders of magnitude compared with an optimized monomeric ligand, LSN3374443 (IC_50_ = 36 nM; *n* = 3) (Fig. 2b,c).

To specify the apo(a) K_IV_ binding interactions of the multivalent and monovalent ligands, we used direct radioligand-binding and ITC methods. For radioligand binding, we created dimeric ^3^H-LSN3441732 and monovalent ^3^H-LSN3374443 radioligands. To identify K_IV_ domains with the capacity to bind to these compounds, individual K_IV_5, K_IV_6, K_IV_7, K_IV_8 and K_IV_10 were evaluated.

Except for K_IV_10, ^3^H-LSN3374443 bound to all tested K_IV_-containing proteins with a similar affinity to that for binding to full-length apo(a) (*K*_d_ values of 15–31 nM) (Extended Data Table 3). In direct binding assays, ^3^H-LSN3374443 bound to full-length recombinant apo(a) with a mean *K*_d_ value of 2.6 nM and an approximate maximum saturable binding (*B*_max_) value of 9.8 fmol ng^−1^ apo(a), equivalent to two to three ligands per molecule of apo(a) (Fig. 2d). In comparison, ^3^H-LSN3441732 bound to full-length apo(a) with a 50-fold affinity increase of *K*_d_ = 0.05 nM and with *B*_max_ = 4.9 fmol ng^−1^ apo(a), indicative of one ligand per molecule of apo(a) (Fig. 2d). Direct ligand-binding experiments using apo(a) K_IV_5–8 tandem domains showed the same potency and stoichiometry relationship; ^3^H-LSN3374443 is less potent than ^3^H-LSN3441732, with *K*_d_ values of 13 nM and 0.33 nM, respectively (Extended Data Table 3). In agreement with the radioligand-binding studies of full-length apo(a), ITC data with apo(a) K_IV_5–8 tandem domains indicated that LSN3374443 binds with 2:1 stoichiometry, whereas LSN3441732 binds with 1:1 stoichiometry (Fig. 2e); both compounds bind with a *K*_d_ value lower than 15 nM. The affinity of the multivalent ligand ^3^H-LSN3441732 for single K_IV_7 (*K*_d_ = 2.8 nM) and K_IV_8 (*K*_d_ = 2.0 nM) was lower compared with its affinity for K_IV_7–8 (*K*_d_ = 0.21 nM) and K_IV_5–8 (*K*_d_ = 0.33 nM), confirming that multivalency results in a substantial improvement in potency (Fig. 2f). These data, along with a crystal structure analysis of LSN3441732 bound simultaneously to two K_IV_8 domains, show that multiple apo(a) K_IV_ domains can be engaged simultaneously, which leads to an improvement in binding affinity and Lp(a) inhibition potency (Extended Data Fig. 2b). Notably, as observed with the monomeric compound LSN3353871, binding of LSN3441732 to K_IV_2 was ruled out using ITC (Extended Data Table 1).

**Fig. 3:**
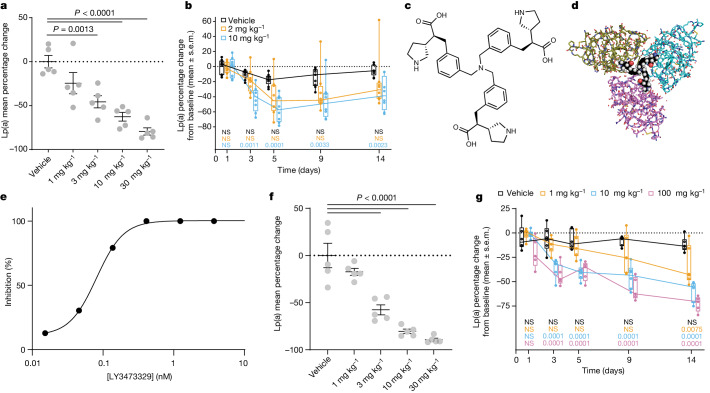
Multivalency increases the potency and efficacy of Lp(a) reduction. **a**, Percentage change in the steady-state levels of Lp(a) in *LPA* × *ApoB100* transgenic mice after five days of BID oral dosing with LSN3441732. Data are from one experiment with *n* = 5 per group, shown as mean ± s.e.m. with individuals plotted as circles. Two-sided *P* values were calculated using one-way ANOVA with Dunnett’s comparison to vehicle. **b**, Percentage change from baseline in the steady-state levels of Lp(a) in female cynomolgus monkeys during 14 days of BID oral dosing (*n* = 8 per group except *n* = 9 for the vehicle group) with LSN3441732 in a single experiment. Box plots as in Fig. 1f. Two-sided *P* values are from a repeated measures ANOVA with Bonferroni comparisons to vehicle at each time point. The null hypothesis was rejected at *P* < 0.05. **c**, Chemical structure of LY3473329. **d**, Crystal structure of LY3473329, shown as a CPK representation, bound to three separate K_IV_8 domains (PDB ID: 8V8Z). **e**, Average inhibition of in vitro Lp(a) assembly by LY3473329 (nine independent experiments; compound concentration 0.03–3 nM). **f**, Percentage change in the steady-state levels of Lp(a) in *LPA* × *ApoB100* transgenic mice after five days of BID oral dosing with LY3473329. Data are from one experiment with *n* = 5 per group, shown as mean ± s.e.m. with individuals plotted as circles. Two-sided *P* values were calculated using one-way ANOVA with Dunnett’s comparison to vehicle. **g**, Percentage change from baseline in the steady-state levels of Lp(a) in male cynomolgus monkeys during 14 days of QD oral dosing (*n* = 6 per group) with LY3473329 in a single experiment. Box plots as in Fig. 1f. Two-sided *P* values are from a repeated measures ANOVA with Bonferroni comparisons to vehicle at each time point. The null hypothesis was rejected at *P* < 0.05. Source Data

In Lp(a) transgenic mice, LSN3441732 plasma concentrations increased in a dose-dependent manner and steady-state plasma Lp(a) levels decreased after five days of oral BID dosing, with an ED_50_ of 4 mg kg^−1^ and a mean maximal inhibition of 79% at the 30 mg kg^−1^ dose (Extended Data Table 4 and Fig. 3a). In cynomolgus monkeys, 2 mg kg^−1^ and 10 mg kg^−1^ oral BID doses of LSN3441732 decreased median Lp(a) levels by a maximum of 45% and 57%, respectively, after 5 days of dosing, and continued to suppress the steady-state levels of Lp(a) for 14 days (Extended Data Table 4 and Fig. 3b). Compatible with a multivalent binding mechanism, LSN3441732 showed improvements in potency and efficacy both in vitro and in vivo compared with the monovalent inhibitor.

Taking further advantage of multivalency, a trimeric molecule, LY3473329, was synthesized (Fig. 3c). Crystal structure analysis of the interactions between LY3473329 and K_IV_8 shows that the trimeric molecule is capable of binding to three K_IV_8 domains simultaneously, which suggests that multi-K_IV_ domain interactions are possible in apo(a) (Fig. 3d and Extended Data Fig. 2c). LY3473329-HCl salt binds to K_IV_8 selectively with a potency of 22 nM and inhibits the formation of Lp(a) particles in vitro with an IC_50_ value of 0.09 nM (*n* = 9) (Extended Data Table 1 and Fig. 3e). In the Lp(a) transgenic mouse model, the plasma levels of LY3473329-HCl salt increased in a dose-dependent manner, and treatment with LY3473329-HCl reduced the levels of Lp(a) with an absolute ED_50_ of 3 mg kg^−1^, with a maximum Lp(a) reduction of 92% after five days of BID oral dosing (Extended Data Table 5 and Fig. 3f). In cynomolgus monkeys, LY3473329 reduced median Lp(a) levels in a dose-dependent manner by up to 71% in the 100 mg kg^−1^ once daily (QD) cohort, compared with baseline (Extended Data Table 5 and Fig. 3g). Multivalency, which allowed the engagement of multiple apo(a) K_IV_ domains, considerably increased the potency and efficacy of inhibitors of Lp(a) formation in both in vitro and in vivo models of Lp(a) reduction, as compared with monovalency (Extended Data Table 6).

Plasminogen is the pro-enzyme precursor of the primary fibrinolytic protease plasmin. Circulating plasminogen, which comprises a Pan-apple domain, five Kringle domains (KI–V) and a serine protease domain, adopts a closed, activation-resistant conformation^26,27^. The regulation of plasminogen activity is tightly controlled to ensure systemic haemostasis, and the inactivation of plasmin is dependent on the physiological inhibitor of plasmin, α2-antiplasmin, which removes free plasmin from circulation. Because apo(a) K_IV_ domains share a high percentage identity with plasminogen Kringle domains, we investigated whether the compounds could modulate plasminogen activity, as measured by plasmin enzymatic activity.

**Fig. 4:**
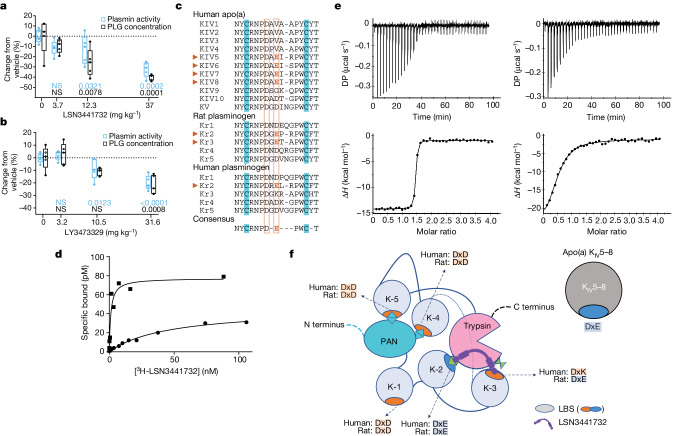
Selectivity against plasminogen. **a**,**b**, Change in plasmin activity (blue) and plasminogen concentration (black) in rat plasma after four days of oral dosing with LSN3441732 (**a**) or LY3473329 (**b**). Data are from one experiment with *n* = 5 per group and individuals are plotted as circles. Two-sided *P* values are from an ANOVA with Dunnett’s comparisons to vehicle. The null hypothesis was rejected at *P* < 0.05. **c**, Primary sequence alignment of human apo(a) K_IV_ domains, rat and human plasminogen Kringle domains and the presence of the consensus binding sequence (DxE) for the binding of the small-molecule inhibitor. **d**, Representative saturation binding curves of ^3^H-LSN3441732 binding to rat (squares; seven experiments) or human (circles; four experiments) plasminogen. **e**, ITC of LSN3441732 binding to rat (left) or human (right) plasminogen. Data are representative of two independent experiments. **f**, Schematic model of human closed plasminogen, on the basis of the crystal structure of human type II plasminogen (PDB ID: 4DUR). Crucial interactions for maintaining the closed conformation are K-4 and K-5 with Lys and Arg from the plasminogen–apple–nematode (PAN) domain, and K-2 with Lys from the trypsin domain. Our hypothesis is that multivalent molecule interactions with the K-2 and K-3 subdomains of rat plasminogen promote a plasminogen open (active) conformation. Box plots show the median (centre line), 25th and 75th percentile (box limits) and the smallest and largest value (whiskers). LBS, lysine-binding site. Source Data

**Extended Data Fig. 3:**
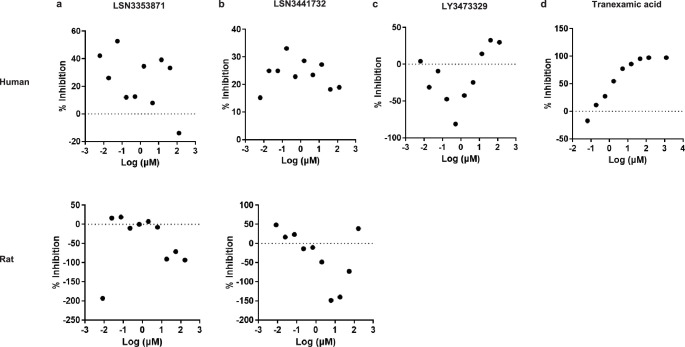
Human and rat in vitro clot dissolution assay. **a**, Percentage inhibition of clot dissolution mediated by LSN3353871 in human platelet-poor plasma (top) or rat citrate plasma (bottom) (compound concentration 0.03–100 µM) as compared to vehicle. **b**, Percentage inhibition of clot dissolution mediated by LSN3441732 in human platelet-poor plasma (top) or rat citrate plasma (bottom) (compound concentration 0.03–100 µM) as compared to vehicle. **c**, Percentage inhibition of clot dissolution mediated by LY3473329 in human platelet-poor plasma (compound concentration 0.03–100 µM) as compared to vehicle. **d**, Percentage inhibition of clot dissolution mediated by plasminogen inhibitor, tranexamic acid (compound concentration 0.1–1,000 µM).

**Extended Data Fig. 4:**
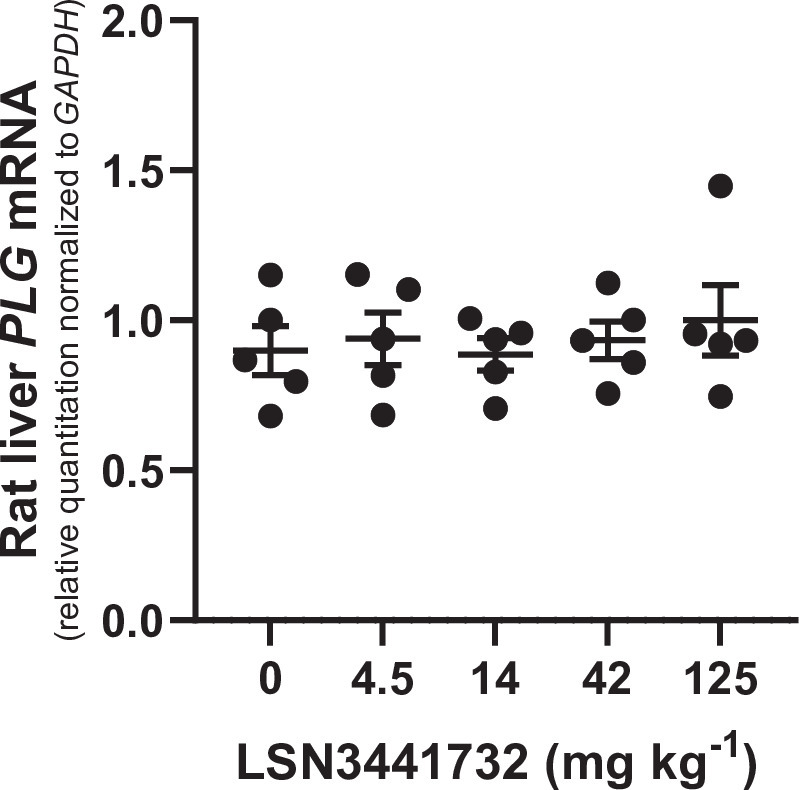
Rat hepatic *Plg* mRNA after oral doses of LSN3441732. Change in *Plg* mRNA in male rat liver after four days of oral dosing with LSN3441732. Data are from one experiment with *n* = 5 per group (circles) with mean ± s.e.m. shown. *Plg* mRNA was measured by RT-qPCR from liver of male Sprague Dawley rats dosed with varying oral doses of LSN3441732 for 4 days. Relative quantification was calculated using the comparative Ct method and *Gapdh* mRNA served as the normalizing control. Source Data

Monomeric and multivalent compounds were added to rat and human plasma in vitro to assess possible interference with plasmin-mediated clot dissolution, but plasmin activity was not affected (Extended Data Fig. 3), providing evidence that Lp(a) inhibitors do not directly modulate plasminogen activation or plasmin activity. Although no inhibitory action on plasmin activity was observed in rats that were treated with the monomeric compound LSN3353871, administration of the multivalent compound LSN3441732 and LY3473329-HCl salt at pharmacological doses decreased plasma plasmin activity as measured ex vivo (Fig. 4a,b). Because adding these compounds to plasma did not affect the enzymatic activity of plasmin in vitro, we investigated mechanisms that might explain the decrease in plasmin activity in animal models. In rats, the levels of hepatic *Plg* mRNA were unchanged after daily oral dosing with the multivalent Lp(a) disruptor LSN3441732 (Extended Data Fig. 4); however, the plasma levels of plasminogen protein were decreased in a dose-dependent manner by both LSN3441732 and LY3473329-HCl salt (Fig. 4a,b). Because these results raised the possibility that the multivalent Lp(a) disruptors could decrease the activity of plasmin in humans, we examined the underlying mechanism that drives the reduction in plasminogen levels mediated by these compounds.

**Extended Data Fig. 5:**
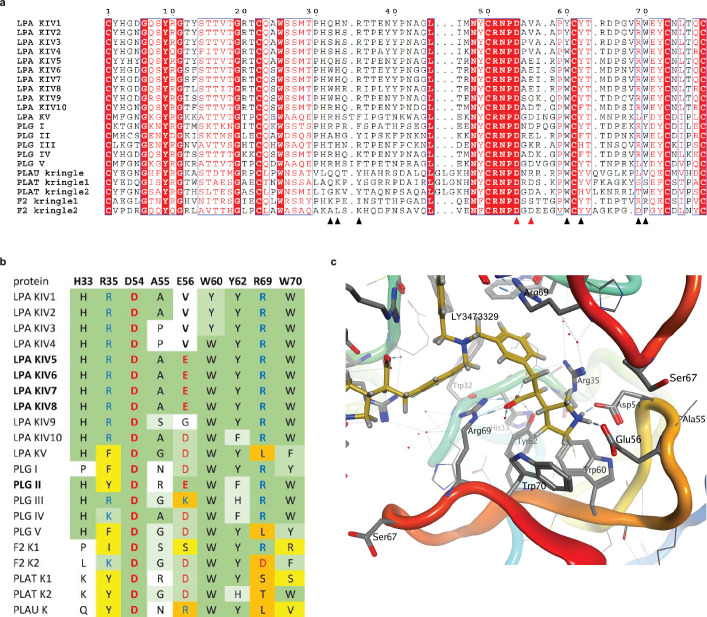
Analysis of Kringle domains in LPA and potential off-target proteins. **a**, Multiple sequence alignment of Kringle domains from LPA, plasminogen (PLG)/angiostatin, plasminogen activator urokinase (PLAU), plasminogen activator tissue type (PLAT, tPA), and prothrombin (F2). Residue numbering is based on the type IV Kringle domains in LPA from the first to the last cysteine, which form a disulfide bond. Residues interacting with compounds published in this study are indicated with black triangles, and the key DxE residues are indicated with red triangles. Disulfides form between Cys1-Cys78, Cys22-Cys61, Cys50-Cys73. **b**, Detailed comparison of LPA Kringle domains with off-targets containing Kringle domains, highlighting variations that are likely to reduce binding affinity. The only off-target Kringle domain that conserves the DxE motif seen in LPA K_IV_5/6/7/8 is that of the human plasminogen Kringle II domain. Yellow squares indicate potentially disruptive variations, and orange squares indicate the most disruptive variations. Asp54 is the only residue strictly conserved across all these human Kringle domains. **c**, LY3473329 is shown binding to one of three copies of LPA K_IV_8; interacting residues are labelled. The protein ribbon is coloured blue to red from the N to the C terminus. Ser67 is also shown, because it is this residue in LPA K_IV_9 (Cys67) that forms a disulfide bond with ApoB (in the absence of inhibitor).

Comparing the primary amino acid sequences of plasminogen from rats, cynomolgus monkeys and humans (Fig. 4c), and understanding the accessibility of Kringle lysine-binding sites in the closed activation-resistant conformation, as illustrated by the human plasminogen structure^26^, we hypothesized that the multivalent compounds would have species-specific plasminogen-binding properties. The structural data with LSN3353871 revealed a molecular interaction between a key motif made up of an aspartic acid (D) residue, any amino acid (x) and a glutamic acid residue (E) (DxE motif) in K_IV_8 (Fig. 1d), so we focused on the presence of these motifs in plasminogen. Notably, in apo(a), each K_IV_ repeat from K_IV_5 through to K_IV_8 contains the DxE motif. Rat plasminogen Kringle (K) K-2 and K-3 domains both contain a DxE motif, whereas this motif is only present in K-2 in human plasminogen (Fig. 4c). We hypothesized that multivalent compounds could act as bivalent binders of K-2 and K-3 in rat plasminogen, whereas they could only act as monovalent binders of human plasminogen through the exclusive interaction with K-2. If this is correct, the multivalent compound should have a higher binding affinity for rat plasminogen than for human plasminogen. Saturation binding experiments with the bivalent ^3^H-LSN3441732 molecule and rat and human plasminogen showed that ^3^H-LSN3441732 had a 50-fold higher affinity for rat plasminogen than for human plasminogen (Fig. 4d). As assessed by ITC, the bivalent molecule bound to the K-2 to K-3 domains of rat plasminogen with an affinity more than 100-fold higher than that for the K-2 to K-3 domains of human plasminogen; the *K*_d_ value for rat K-2 to K-3 was less than 10 nM, whereas the *K*_d_ value for human K-2 to K-3 was 1.6 µM (Fig. 4e). We hypothesize that the consequence of multivalent molecule binding to rat plasminogen in K-2 to K-3 breaks the K-2–Lys708 interaction found in closed plasminogen and triggers an open conformation that favours rapid degradation (Fig. 4f), thus explaining the reduction in plasmin activity in animals treated with LSN3441732 and LY3473329-HCl salt. Evidence that this difference in potency between rat and human plasminogen binding translates into oral doses that are capable of lowering the levels of Lp(a) without affecting plasmin activity can be found in the human phase 1 study of LY3473329 (muvalaplin)^28^. Analyses of other potential off-target protein interactions through multiple sequence alignments of the full Kringle domains from LPA, PLG, plasminogen activator urokinase (PLAU), plasminogen activator tissue type (PLAT) and prothrombin (F2) support the compound selectivity rationale (Extended Data Fig. 5a). A comparison of residues near the inhibitor-binding site (Extended Data Fig. 5b) reveals that only human PLG K-2 preserves the DxE side chains that make key interactions with LY3473329 (Extended Data Fig. 5c).

In summary, we report the discovery and optimization of potent and selective small-molecule inhibitors of Lp(a) formation by exploiting the repeated K_IV_ domain structure of apo(a) through compound multivalency. Gaining a mechanistic understanding of the engagement between such compounds and apo(a) enabled us to develop molecules that are selective for apo(a) over plasminogen in humans. These results provide a proof of concept that an orally delivered small-molecule inhibitor of Lp(a) might be a therapeutic option for patients with high levels of Lp(a) who are at risk of either atherosclerotic cardiovascular disease or calcific aortic valve disease.

## Methods

### Compounds

Chemical synthesis and characterization of the compounds were performed as indicated in the Supplementary Information.

### Recombinant protein expression

The nucleotide sequence encoding the human *LPA* gene (NP_005568.2) was inserted into a mammalian expression vector containing a cytomegalovirus promoter. Protein expression was performed by transient transfection of human embryonic kidney 293 (HEK293) cells cultured in serum-free medium. The culture medium was collected five days after transfection and human apo(a) protein was purified using l-lysine affinity chromatography. Nucleotide sequences encoding various K_IV_ domains of human apo(a) and human and rat plasminogen were inserted into a pET21a *Escherichia coli* expression vector. Bacterial BL21(DE3) was used as the expression host, and the induction of protein expression was performed in 2× TY medium with 1 mM isopropyl β-d-1-thiogalactopyranoside at 37 °C for 5 h. Cells were collected and stored at –80 °C for subsequent protein purification and refolding. The frozen cell pellets were lysed and inclusion bodies were purified. The protein refolding was performed using the rapid-dilution method, and the refolded proteins were further purified by size-exclusion chromatography. All protein concentrations were determined by A280.

### ITC

Experiments were performed on a MicroCal Auto-iTC200 titration calorimeter (Malvern Panalytical), with a cell volume of 200 μl and a 40-μl microsyringe. Experiments were performed at 25 °C while stirring at 750 rpm in ITC buffer (50 mM phosphate buffer at pH 8.0). The microsyringe was loaded with a solution of the respective compounds, with the concentration measured by quantitative nuclear magnetic resonance, and was inserted into the calorimetric cell prefilled with the respective recombinant proteins of known concentration in ITC buffer. The system was equilibrated to 25 °C and an extra delay of 60 s was applied. The first titrations were performed using an initial control injection of 0.5 μl, followed by 18 identical injections of 2 μl, with a duration of 4 s per injection and 150-s intervals between injections. The second titration was performed over the first titration without cleaning the cell and was carried out with continuous injections as an automation method in a MicroCal Auto-iTC200. The second titrations were performed using 19 identical injections of 2 μl, with a duration of 4 s per injection and 150-s injection intervals^29^. The data were corrected for ligand heats of dilution and were deconvoluted using MicroCal PEAQ-ITC Analysis Software to yield the enthalpy of binding (Δ*H*) and the binding constant (*K*). Thermodynamic parameters were calculated using the basic equation of thermodynamics (Δ*G* = Δ*H* – *T*Δ*S* = –*RT*ln*K*_d_), where Δ*G*, Δ*H* and Δ*S* are the changes in free energy, enthalpy and entropy of binding, respectively; *K*_d_ is the equilibrium dissociation constant; *T* is the absolute temperature (kelvins); and *R* is the gas constant (1.987 cal mol^−1^ K^−^^1^). A single-binding-site model was used in MicroCal PEAQ-ITC Analysis Software v.0.9.

### Lp(a) assembly assay

HepG2 cells (HB-8065, ATCC) were cultured in Dulbecco’s modified Eagle’s medium, supplemented with 10% fetal bovine serum, 20 mM 4-(2-hydroxyethyl)-1-piperazineethanesulfonic acid (HEPES) and 100 U ml^−1^ penicillin–streptomycin. apoB-conditioned media were collected after 24 h of culture at 37 °C and 5% CO_2_ from confluent wild-type HepG2 cells that endogenously express and secrete apoB. Apo(a)-conditioned media were collected after 24 h of culture at 37 °C and 5% CO_2_ from HEK293 cells (CRL-1573, ATCC) that stably express and secrete recombinant human 16K apo(a) protein containing one K_IV_ type 1, six K_IV_ type 2 repeats, one each of K_IV_ types 3–10 and one K_V_.

The assembly assay was conducted in 96-well polypropylene plates by combining HepG2 and apo(a)-HEK293-conditioned media in the presence of a range of compound concentrations. After a two-hour incubation period at 37 °C, the reaction was stopped by the addition of 6-aminocaproic acid. Lp(a) was detected using a sandwich enzyme-linked immunosorbent assay (ELISA), with a polyclonal goat anti-apo(a) capture antibody (Abcam ab31675) and a biotin-conjugated, polyclonal goat anti-apoB detection antibody (Abcam ab20898). In brief, ELISA plates were coated with 100 μl of the capture antibody at a 1:12,500 dilution (around 4 μg ml^−1^) into HEPES-buffered saline (HBS), incubated overnight at 4 °C, washed and blocked by the addition of 200 μl of 1% casein. After removal of the blocking buffer, 20 μl of each of the assembly reactions or of a dilution series of purified human Lp(a) (Athens Research, 12-16-121601), diluted 1:5 into HBS supplemented with 0.5% casein, were added to the ELISA plate. After a 2-h incubation period at room temperature, plates were washed five times with HBS + 0.1% Tween-20. To each well, 100 μl of biotin-conjugated detection antibody was added and allowed to bind for one hour at room temperature. Plates were washed five times with HBS + 0.1% Tween-20, and 100 µl of streptavidin–horseradish peroxidase (HRP) was added to each well, followed by another one-hour incubation at room temperature. Unbound streptavidin–HRP was removed by washing plates five times with HBS + 0.1% Tween-20, and the ELISA was developed using the HRP substrate 3,3’,5,5’-tetramethylbenzidine (TMB). The reaction was stopped after 15 min by the addition of 1N sulfuric acid. Absorbance at 450 nm was measured on an Envision plate reader.

The concentration of Lp(a) formed in each test condition was determined on the basis of the standard curve constructed from the purified human Lp(a) used as a reference standard, and was normalized to high (buffer) and low (no apoB) controls. Data were fitted to a standard Hill equation. Data were analysed in GraphPad Prism v.9.5.1.

### Protein crystallography

The K_IV_7 and K_IV_8 domains (1263–1357 and 1377–1470, respectively, on the basis of GenBank NP_005568.2) were expressed in *E. coli* BL21(DE3) cells; inclusion bodies were purified by repeated centrifugation and resuspension, then unfolded in 6 M guanidine hydrochloride, diluted in Tris, NaCl, 0.5 M urea and 1.25 mM oxidized and reduced glutathione. After dialysis with Tris pH 8, the protein was purified and concentrated to 22 mg ml^−1^ (final buffer 50 mM Tris pH 9, 150 mM NaCl and 10% glycerol). For the monovalent compound, tagless K_IV_8 was set up in vapour diffusion sitting drops at 21 °C at a ratio of 1.5:1, with a well solution of 100 mM HEPES pH 6 and 1 M tri-sodium citrate pH 7. Crystals appeared within one day and were transferred on the 30th day to a soaking drop containing 10 mM LSN3353871, 100 mM HEPES pH 6 and 1 M tri-sodium citrate pH 7. They were sealed and left at 21 °C for two hours, then transferred to a drop containing 10 mM LSN3353871, 100 mM HEPES pH 6, 1 M tri-sodium citrate pH 7 and 22% glycerol, then collected and flash frozen in liquid nitrogen. For the bivalent compound LSN3441732, K_IV_7 was set up with a well solution of 100 mM MES pH 6.5 and 1.6 M magnesium sulfate. After 11 days, the crystal was collected in similar conditions plus 22% glycerol and flash frozen in liquid nitrogen. The trimeric compound, LY3473329, was set up at 2 mM with tagless protein in vapour diffusion sitting drops at 21 °C with a well solution of 100 mM sodium acetate pH 4.5 and 30% PEG 300. After two weeks, a plate-shaped crystal was collected in similar conditions with 22% ethylene glycol and was flash frozen in liquid nitrogen.

Diffraction data were collected at the Lilly Research Laboratories Collaborative Access Team (LRL-CAT) beamline at Sector 31 of the Advanced Photon Source. Crystals stored in liquid nitrogen were mounted on a goniometer equipped with an Oxford Cryosystems cryostream maintained at a temperature of 100 K. The wavelength used was 0.9793 Å, collecting 900 diffraction images at a 0.2° oscillation angle and a 0.12-s exposure time on a Pilatus3 S 6M detector at a distance of 392 mm. The diffraction data were indexed and integrated using MOSFLM 7.0.5 and merged and scaled with Scala 3.3 and Truncate 6.5 from the CCP4 6.5 suite^30^.

Non-isomorphous data readily yielded the initial structure by molecular replacement using an internal proprietary crystal structure. The initial structure coordinates for the dataset were further refined using REFMAC v.5.8 (CCP4), applying anisotropic temperature factors. Model building was performed with Coot v.0.8 (CCP4) and final structure validation with MolProbity v.4.02 (ref. ^31^) and CCP4 validation tools. See Supplementary Table 1 for crystallographic data statistics.

Protein coordinates and structure factors have been deposited with the Protein Data Bank (https://www.wwpdb.org/) under the access codes 8TCE (LSN3353871), 8V9B (bivalent LSN3441732) and 8V8Z (trimeric LY3473329).

### In vivo studies

All procedures were conducted in compliance with the Animal Welfare Act, the Guide for the Care and Use of Laboratory Animals and the Office of Laboratory Animal Welfare (Covance Laboratories for cynomolgus monkey studies and Eli Lilly and Company for mouse and rat studies).

### *LPA* transgenic mouse studies

The *LPA* gene (GenBank accession code NM_005577) encoding the human apo(a) protein with a signal peptide, one K_IV_ type 1, six K_IV_ type 2 repeats, one each of K_IV_ types 3–10, one K_V_ and one protease domain was subcloned into a transgenic vector containing a mouse albumin promoter cassette and a human growth hormone polyadenylation signal. The transgenic mouse line was generated by pronucleus injection of the *LPA* transgenic vector. Eight individual transgenic founder mice were assessed for germline transmission of *LPA* by genotyping and assessment of the levels of apo(a) in the plasma. Positive founder mice were crossed with *ApoB100* transgenic mice (Taconic model 1004), and the resulting mouse line nomenclature (B6.SJL-Tg(APOB)1102Sgy Tg(Alb-LPA)32Arte) was selected on the basis of the confirmation of germline transmission, the identification of *LPA* and *huApoB100* transgenes and the levels of Lp(a) in the plasma. Mice hemizygous for both transgenes were used for pharmacology studies. The studies were conducted in female Lp(a) double transgenic mice (age around 7–17 months). Mice were housed in cages with a standard light cycle (12-h light, 12-h dark), at room temperature (22 ± 4 °C), with a relative humidity of 30–70%. Mice were identified by numbers on the cage cards. After arrival, mice were fed on a normal diet (Harlan Teklad diet 2014). Mice were randomized to treatment groups (*n* = 5 per group, unless otherwise noted) by body weight and baseline plasma Lp(a) concentration using a block randomized allocation tool (BRAT) for the study. In an example Lp(a) transgenic mouse study in which 66 mice were prescreened for baseline Lp(a) levels, the average Lp(a) level was 45 ± 1.8 µg ml^−1^ (range 20–78 µg ml^−1^). Mice were dosed with various compounds in vehicle (10 ml kg^−1^, 1% hydroxyethyl cellulose (HEC), 0.25% Tween-80, Antifoam) or with vehicle alone as a control orally BID for five days. Blood samples were collected by tail bleeds at designated times, and Lp(a) levels were determined by ELISA by an investigator who was blinded to group allocation. In brief, lipoprotein particles were captured by a goat anti-Lp(a)-antibody-coated plate (Abcam ab31675, diluted 1:12,500 in HEPES-buffered saline), the plates were washed and samples were detected using an HRP-conjugated goat anti-apoB antibody (Abcam ab27622, diluted 1:3,000). Colorimetric peroxidase substrate 3,3’,5,5’-TMB was added, and the reaction was stopped using 1 N sulfuric acid. Absorbance at 450 nm was read on a Molecular Devices SpectraMax plate reader. Blood samples were collected at four hours after dosing on day 5 of dosing to confirm exposure. Full pharmacokinetic (PK) profiles were not evaluated. Concentrations of compounds were evaluated from dried blood spots through a non-Good Laboratory Practice (GLP) liquid chromatography with tandem mass spectrometry (LC–MS/MS) assay. Data were analysed in GraphPad Prism v.9.5.1

### Cynomolgus monkey studies

Female or male Chinese *Macaca fascicularis* cynomolgus monkeys (as described in the figure legends) of unspecified ages with body weight ranging from 2.0 kg to 5.0 kg were housed in cages with a standard light cycle (12-h light, 12-h dark) at room temperature (20–26 °C); the relative humidity ranged from 30% to 70%. Monkeys were given fruits, vegetables or dietary enrichment as a form of environmental enrichment, and various cage enrichment devices. Purina Lab diet 5048C was provided BID and individual monkeys were identified by cage card. The monkeys were randomized to treatment groups by body weight and baseline plasma Lp(a) concentration, as determined by a commercially available Randox assay (Randox LP2757), using a BRAT for the study. In an example cynomolgus monkey study in which 39 monkeys were prescreened for baseline Lp(a) levels, the average Lp(a) level was 350 ± 57 µg ml^−1^ (range 53–1,667 µg ml^−1^). Monkeys were dosed orally with vehicle (purified water) or compounds QD or BID for 14 days, at dose levels and frequencies as previously described for each compound. Plasma samples were collected on days before dosing to establish baseline Lp(a) and after the morning dose during the study. Lp(a) levels were measured by an investigator who was blinded to group allocation using the Randox assay, and were quantified relative to the calibrator series (Randox LP3404). Data were analysed in GraphPad Prism v.9.5.1. The Lp(a) percentage change from before dosing in non-human primates was analysed by repeated measures ANOVA in SAS v.9.4 (SAS). At each time point, the Lp(a) percentage change for each treated group was compared with vehicle by the Bonferroni method.

Plasma samples were collected from all monkeys before dosing and at four-hour time points only on days 1, 3, 5, 9 and 14. A full PK profile was obtained in the first three monkeys of each dose group on day 15, collecting samples before dosing and 1, 2, 4, 8, 12, 24, 48 and 96 h after dosing. Concentrations of compounds were evaluated in plasma samples using a non-GLP LC–MS/MS assay. The non-compartmental plasma PK parameters were calculated using Watson (v.7.5).

### Radioligand-binding assays

Full-length apo(a) and various shorter constructs were prepared as described. Human and rat plasminogen, purified from plasma by affinity chromatography, were obtained from Innovative Research (IHGPG and IRPLG, respectively).

All reagents used in the binding assays were prepared in assay buffer containing 50 mM Tris-HCl, pH 7.4, and 0.1% bovine serum albumin. All apo(a) proteins were diluted to a final concentration of 200 pM. Human and rat plasminogen were diluted into assay buffer, and 250 ng was added to each well.

Saturation binding studies of ^3^H-LSN3441732 or ^3^H-LSN3374443 were conducted by adding to each well of a white-wall, clear-bottom 96-well plate, 50 µl each of (1) dimethyl sulfoxide (DMSO) or a saturating concentration (40 μM) of an unlabelled ligand; (2) protein diluted into assay buffer; (3) resuspended wheat germ agglutinin polyvinyltoluene scintillation proximity assay beads (2 mg ml^−1^); and (4) a dilution series of radioligand. Plates were incubated for one to two hours at room temperature and counted on a Wallac Trilux 1450 liquid scintillation counter. Non-specific binding, defined as binding in the presence of the unlabelled ligand, was subtracted to determine specific binding. Specific binding was plotted as a function of ligand concentration, and a single-site binding equation of the form *y* = *B*_max_ × *x*/(*K*_d_ + *x*) was fitted to the data, where *B*_max_ is the maximum saturable binding and *K*_d_ is the equilibrium dissociation constant.

### Human and rat in vitro clot dissolution assay

Platelet-poor plasma was generated from whole blood donated by healthy volunteers, and Sprague Dawley rat citrate plasma was purchased from Innovative Research. One hundred microlitres of plasma was added to a 96-well plate (Corning) and the following were added to the indicated concentrations: 7.5 mM CaCl_2_, 117 mM NaCl and 5 µl of test compound in 50% DMSO. Wells were mixed and warmed to 37 °C. A mixture of thrombin (Sigma) and tissue plasminogen activator (Calbiochem) was prepared. Final concentrations in the reaction were 0.05 NIH U ml^−1^ and 0.5 nM, respectively. The mixture was added and the plate was sealed and placed in a prewarmed plate reader, shaken and read every 2 min for 15 h. The maximum slope for each curve was determined and the percentage inhibition was calculated relative to untreated controls^32^. Data were analysed in GraphPad Prism v.9.5.1.

### Rat *Plg* mRNA, plasminogen activity assay and plasminogen ELISA

Male Sprague Dawley rats (Charles River, average body weight 310 g, aged eight to nine weeks old) were housed in cages with a standard light cycle (12-h light, 12-h dark), at room temperature (22 ± 4 °C), with a relative humidity of 30–70%. Rats were identified by numbers on the cage cards. After arrival, rats were fed on a normal diet (Harlan Teklad diet 2014) and randomized to treatment groups (*n* = 5 per group) by baseline plasma plasminogen concentration and activity using a BRAT. LSN3441732 and LY3473329 were administered QD orally at three dose levels (3.7, 12.3 and 37 mg kg^−1^; 3.2, 10.5 and 31.6 mg kg^−1^ per dose, respectively) or vehicle (10 ml kg^−1^, 1% HEC, 0.25% Tween-80, Antifoam) for four days. Terminal blood was drawn under general anaesthesia 24 h after the day-4 oral dose. Plasma samples were collected via the abdominal aorta into a premeasured anticoagulant BD Vacutainer used for blood collection (2.7 ml, buffered sodium citrate). The liver was flash frozen in liquid nitrogen.

RNA was isolated from the liver using the PureLink RNA Mini Kit (Invitrogen) and quantified using the NanoDrop spectrophotometer (Thermo Fisher Scientific). Complementary DNA (cDNA) was prepared using the High Capacity cDNA Reverse Transcriptase Kit (Applied Biosystems). Quantitative PCR was conducted using TaqMan reagents and primers (*Plg* Rn00585167_m1, Gapdh Rn99999916_s1). Relative quantitation was calculated using the comparative Ct method. Statistical significance was evaluated using JMP v.16.1.0 software (SAS).

To measure plasminogen activity, 100 µl plasma per well was added to a 96-well plate (Corning) and the following were added to the indicated concentrations: 7.5 mM CaCl_2_ and 117 mM NaCl. Wells were mixed and warmed to 37 °C. Urokinase (Millipore) was added at 125 U per well and the plate was incubated at 37 °C for 4 min. The plasminogen substrate Chromogenix S-2251 (DiaPharma) was added to a concentration of 3.3 mM. The mixture was added and the plate was sealed and placed in a prewarmed plate reader, shaken, and the absorbance read every 30 s for 2 h. The maximum slope for each curve was determined and the percentage inhibition was calculated relative to untreated controls. Plasminogen protein was measured using ELISA kits for rat (Abcam ab157740) according to the manufacturer’s instructions. Plasminogen activity (percentage) and mass (µg ml^−1^) in rat were analysed by ANOVA in JMP 16.1.0. The percentage reduction of plasminogen activity and mass for each treated group was compared with vehicle by Dunnett’s method.

### Reporting summary

Further information on research design is available in the Nature Portfolio Reporting Summary linked to this article.

## Online content

Any methods, additional references, Nature Portfolio reporting summaries, source data, extended data, supplementary information, acknowledgements, peer review information; details of author contributions and competing interests; and statements of data and code availability are available at 10.1038/s41586-024-07387-z.

### Supplementary information

Supplementary Methods This file is composed of the synthesis data, characterization data and spectra of the compounds described.

Reporting Summary

Supplementary Table 1 This table presents the crystallography data collection and refinement statistics.

### Source data

Source Data Fig. 1

Source Data Fig. 3

Source Data Fig. 4

Source Data Extended Data Fig. 4

## Figures and Tables

**Extended Data Table 1:**
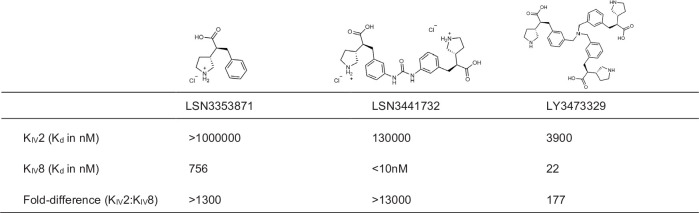
Assessment of apo(a) K_IV_ domain binding selectivity using ITC

ITC of compounds with apo(a) domain K_IV_2 or K_IV_8.

**Extended Data Table 2:**
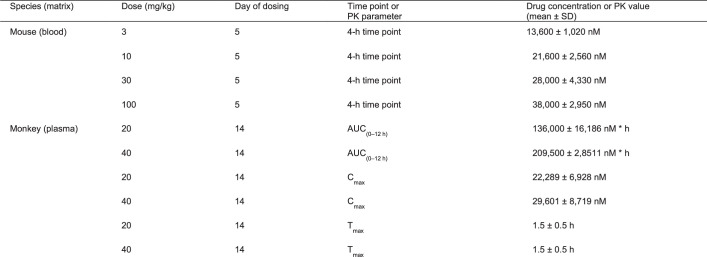
Pharmacokinetics of LSN3353871 in mice and monkeys after oral administration

Abbreviations: AUC(_0–12 hr_), area under the curve from 0 to 12 h after dosing; *C*_max_, maximal concentration; PK, pharmacokinetic; SD, standard deviation; *T*_max_, time at which *C*_max_ occurs.

**Extended Data Table 3:**
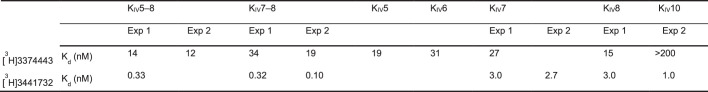
Radioligand binding to recombinant apo(a) K_IV_ subdomains

Results are shown from either one or two independent experiments. Exp 1, Experiment 1; Exp 2, Experiment 2. K_IV_, Kringle IV domain; *K*_d_, equilibrium dissociation constant.

**Extended Data Table 4:**
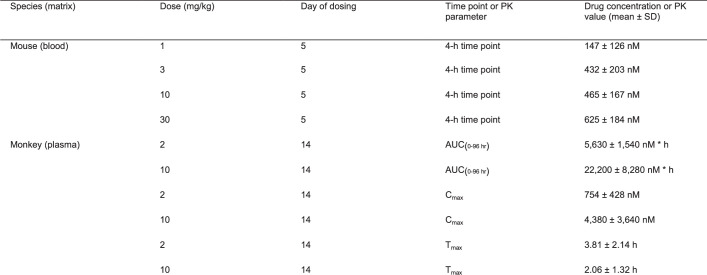
Pharmacokinetics of LSN3441732 in mice and monkeys after oral administration

Abbreviations: AUC(_0–96 hr_), area under the curve from 0 to 96 h after dosing; *C*_max_, maximal concentration; PK, pharmacokinetic; SD, standard deviation; *T*_max_, time at which *C*_max_ occurs.

**Extended Data Table 5:**
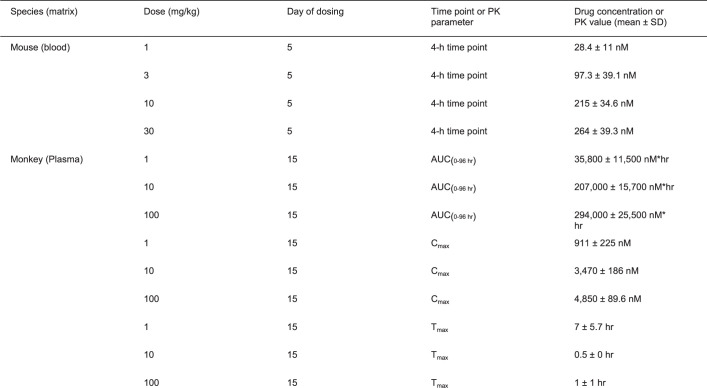
Pharmacokinetics of LY3473329 in mice and monkeys after oral administration

Abbreviations: AUC(_0–96 hr_), area under the curve from 0 to 96 h after dosing; *C*_max_, maximal concentration; PK, pharmacokinetic; SD, standard deviation; *T*_max_, time at which *C*_max_ occurs.

**Extended Data Table 6:**
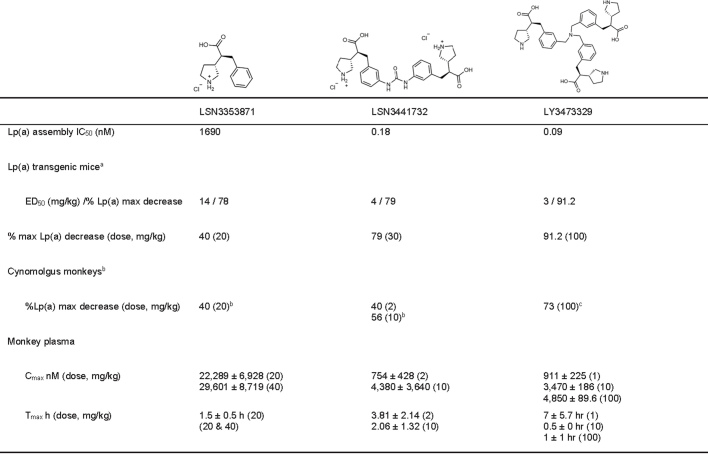
Summary comparison of in vitro and in vivo characterization of compounds

^a^BID, 5 days dosing. ^b^BID, 14 days dosing. ^c^QD, 14 days dosing.

Abbreviations: IC_50_, half-maximal inhibitory concentration; ED_50_, median effective dose; *C*_max_, maximal concentration; *T*_max_, time at which *C*_max_ occurs; BID, twice a day; QD, once a day.

## Data Availability

All data supporting the in vivo findings are available within this article and in the extended data. The coordinates and structure factor files have been deposited in the Worldwide Protein Data Bank (https://www.wwpdb.org/) under the following accession numbers: 8TCE (K_IV_8 + LSN3353871), 8V9B (K_IV_7 + LSN3441732), and 8V8Z (K_IV_8 + LY3473329). Source data are provided with this paper.
