# Supplementary material for: Discovery of potent small-molecule inhibitors of lipoprotein(a) formation
Source: Nature. 2024 May 8;629(8013):945–50. doi: 10.1038/s41586-024-07387-z (PMC11111404; doi:10.1038/s41586-024-07387-z)
Supplement: Supplementary file 1 — This file is composed of the synthesis data, characterization data and spectra of the compounds described. [file 41586_2024_7387_MOESM1_ESM.pdf]

nature portfolio

https://doi.org/10.1038/s41586-024-07387-z

Supplementary information

Discovery of potent small-molecule inhibitors of lipoprotein(a) formation

In the format provided by the authors and unedited

Supplementary Information for

Discovery of potent small-molecule inhibitors of lipoprotein(a) formation

Table of Contents

Supplementary Methods:

Synthesis and characterization data of the compounds described:

General Notes 2
Compound LSN3441732 3
Compound LY3473329 11
Compound LSN3353871 18
Compound LSN3374443 21
Compound ^{3}H-LSN3441732 23

Spectra of the compounds described:

Compound LSN3441732 25
Compound LY3473329 29
Compound LSN3353871 33
Compound LSN3374443 37
Compound ^{3}H-LSN3441732 41

Table with abbreviations 44

Supplementary Methods

Synthesis and characterization of the compounds described.

General notes:

Synthesis of ^{3}H-LSN3374443 is described in WO 2020/247429 Al. Synthesis and characterization of LSN3441732, LY3473329, LSN3353871 and LSN3374443 was carried out at Lilly Research Laboratories. Enantiomeric excess of LY3473329 was determined by HPLC at Asymchem. The synthesis of radiolabeled materials was carried out at Pharmaron UK.

All solvents and starting materials were obtained from commercial sources and used as received. ^{1}H-nuclear magnetic resonance (^{1}H-NMR) spectra were recorded using Bruker NMR spectrometer 400 MHz instrument and ^{13}C-nuclear magnetic resonance (^{13}C-NMR) spectra were recorded on Bruker 100 MHz instrument. Chemical shifts were assigned in ppm relative to respective solvent signals (e.g., signal of residual $CHCl_3$ present in $CDCl_3$ for ^{1}H-NMR). The following abbreviations are used to describe signal splitting patterns: singlet (s), doublet (d), triplet (t), and multiplet (m).

LCMS (ESI) analyses for sample characterization were performed using an Agilent LCMS 1260 instrument, equipped with DAD and MS detectors. LC analyses were obtained after injection of the sample onto a XBridge C18 column (3.5 µm, 2.1 x 50 mm). Chromatographic method in basic media using 10mM ammonium bicarbonate pH 9.0 in channel A and CH_3CN in channel B. Flow Rate: 1.2mL/min; Tª 50ºC; gradient mode: from 5 to 95% B in 1.5 min, hold 0.5 min at 95%B. UV detection was registered at 214 and 300 nm. MS-ESI detection was acquired between 100 and 800 amu. High-resolution mass spectra (HRMS) were recorded using an electrospray ion source (ESI). ESI analysis was performed using an Agilent Accurate-Mass QTOF LCMS instrument, model 6530, equipped with a JetStream ESI Source with two nebulizers. The main nebulizer was hyphenated to a HPLC Agilent 1290 system for sample introduction, and the second one to an isocratic HPLC pump for a continuous introduction of external standards to be used for continuous calibration of the spectra. Diastereomeric excesses (de) were determined by ^{1}H-NMR, high-performance liquid chromatography (HPLC) or supercritical fluid chromatography (SFC) in an

Agilent 1200 Analytical SFC-MS system. The retention times (t_R) described in the chromatograms are those obtained under the conditions described in each case and for a particular equipment configuration; any change may result in modifications to the retention time. Optical rotations were measured on an Anton Paar MCP 500 polarimeter with $[\alpha]_D^{23}$ values reported in degrees; concentration (c) is in g/100 mL.

Synthesis of compound LSN3441732

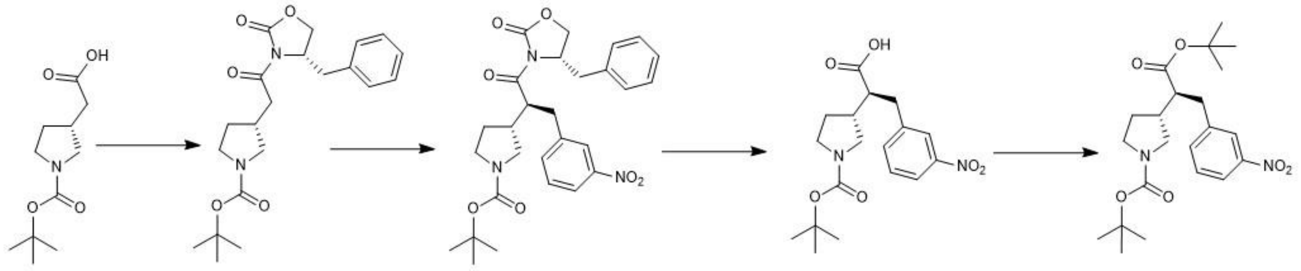

2 **3A** **4A** **5A**

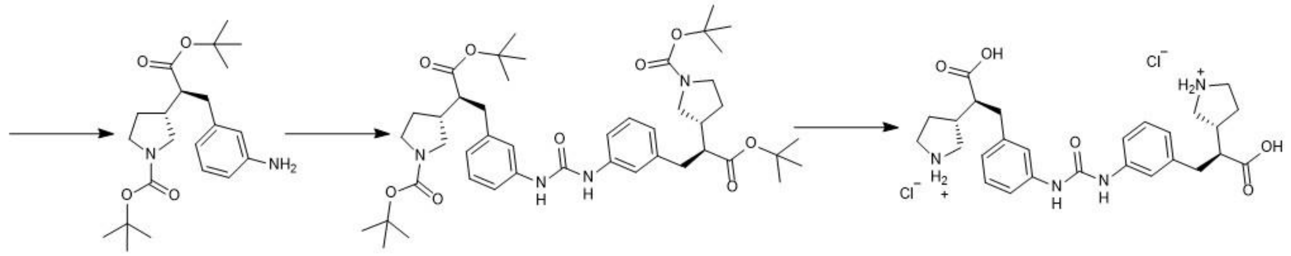

6 **7** **LSN3441732**

<u>*tert*-Butyl (*R*)-3-(2-((*S*)-4-benzyl-2-oxooxazolidin-3-yl)-2-oxoethyl)pyrrolidine-1-carboxylate (**2**)</u>

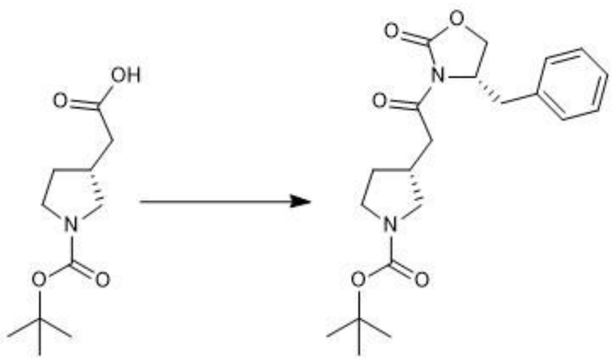

Add triethylamine (56.5 g, 77.9 mL, 559 mmol, 2.5 equivalent) to a solution of (*R*)-2-(1-(tert-butoxycarbonyl)pyrrolidin-3-yl)acetic acid (53.8 g, 235 mmol, 1.05 equivalent) in THF (540 mL) maintained at 10 ºC. After 5 min, add pivaloyl chloride (33.7 g, 34.2 mL, 279 mmol, 1.25 equivalent). After 15 min, add lithium chloride (11.8 g, 279 mmol, 1.25 equivalent) in THF (540 mL) and (4*S*)-4-benzyloxazolidin-2-one (40 g, 223 mmol, 1 equivalent). Allow the mixture to warm to room temperature and stir for 24 h. After 24 h, add 1N aqueous HCl (500 mL) and separate the organic phase from the aqueous phase. Wash the organic phase with 1N aqueous NaOH (500 mL) and saturated aqueous NaCl (500 mL). Separate the layers and dry the organic layer over $MgSO_4$, filter, and concentrate the solution in vacuo. Suspend the residue in a mixture of MeOH/water (1:2, 575 mL) and stir at room temperature overnight. Filter off the solid, wash with hexanes (2 × 150 mL), and dry the solid to give the title compound (65.7 g, 76%). The diastereomeric excess was determined to be >95% by ^{1}H-NMR spectroscopy; the diastereomeric proton resonances of the minor isomer (separated from the proton signals of the major isomer) were not observed in the ^{1}H-NMR spectra of the final product. LCMS (ESI): *m/z* 333.2 [M+H-*tert*-butyl]$^+$. ^{1}H-NMR (400.13 MHz, $CDCl_3$) δ 7.38-7.28 (m, 3H), 7.23-7.17 (m, 2H), 4.73-4.63 (m, 1 H), 4.27-4.16 (m, 2H), 3.75-3.61 (m, 1H), 3.55-3.39 (m, 1H), 3.38-3. 25 (m, 2H), 3.13-2.91 (m, 3 H), 2.84-2.73 (m, 1H), 2.73-2.61 (m, 1H), 2.16-2.06 (m, 1H), 1.64-1.58 (m, 1H), 1.47 (s, 9H).

tert-Butyl (*R*)-3-((*S*)-1-((*S*)-4-benzyl-2-oxooxazolidin-3-yl)-3-(3-nitrophenyl)-1-oxopropan-2-yl)pyrrolidine-1-carboxylate (**3A**)

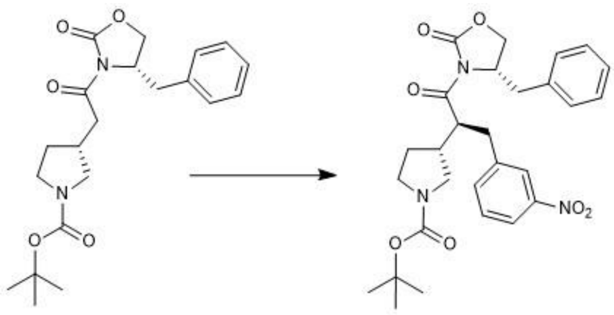

Add lithium bis(trimethylsilyl)amide (1.0 mol/L in THF, 46.33 mL, 46.33 mmol, 1.2 equivalent) to a solution of *tert*-butyl (*R*)-3-(2-((*S*)-4-benzyl-2-oxooxazolidin-3-yl)-2-oxoethyl)pyrrolidine-1-carboxylate (15 g, 38.61 mmol, 1 equivalent) in THF (5 mL/g, 75 mL) at –20 ºC. Stir the mixture at –20 ºC for 20 min. Add a solution of 1-(bromomethyl)-3-nitro-benzene (9.17 g, 42.47 mmol, 1.1 equivalent) in THF (3 mL/g, 45 mL). Stir the solution for 2 h and allow it to warm to room temperature. Dilute the mixture with MTBE and quench with a saturated solution of NH_4Cl, separate the phases, and extract the aqueous phase with MTBE (1×). Combine the organic extracts and sequentially wash the organic extracts with water and brine. Separate the layers and dry the organic layer over Na_2SO_4, filter, and concentrate the filtrate under reduced pressure. Triturate the residue with a mixture of MeOH/H_2O (2:1, 150 mL). Stir the slurry overnight. Filter to collect the solid and wash with hexanes. Dry the solid under vacuum at 40 ºC to give the title compound (19 g, 88%). LCMS (ESI): *m/z* 468.1 [M+H-*tert*-butyl]$^+$. ^{1}H-NMR (400.13 MHz, $CDCl_3$) δ 8.18-8.04 (m, 2H), 7.68-7.58 (m, 1H), 7.52-7.42 (t, *J* = 7.9 Hz, 1H), 7.30-7.21 (m, 3H), 7.04-6.97 (m, 2H), 4.66-4.57 (m, 1H), 4.52-4.42 (m, 1H), 4.16-3.99 (m, 2H), 3.75-3.44 (m, 2H), 3.29-2.96 (m, 5H), 2.62-2.46 (m, 1H), 2.30-2.13 (m, 1H), 2.00-1.90 (m, 1H), 1.80-1.64 (m, 1H), 1.46 (s, 9H).

<u>(*S*)-2-((*R*)-1-(*tert*-Butoxycarbonyl)pyrrolidin-3-yl)-3-(3-nitrophenyl)propanoic acid (**4A**)</u>

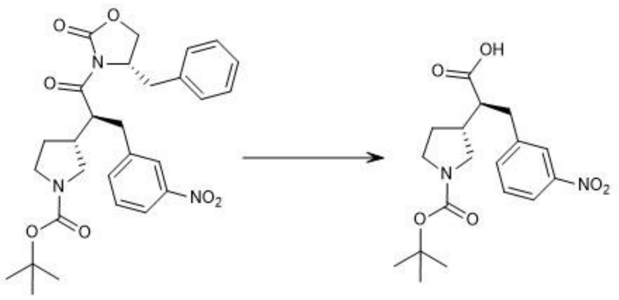

Add lithium hydroxide (11.46 mL, 11.46 mmol, 1.5 equivalent, 1M in water) and hydrogen

peroxide (5.9 mL, 61.12 mmol, 8 equivalent, 35 mass %) to a solution of *tert*-butyl (*R*)-3-((*S*)-1-((*S*)-4-benzyl-2-oxooxazolidin-3-yl)-3-(3-nitrophenyl)-1-oxopropan-2-yl)pyrrolidine-1-carboxylate (4 g, 7.639 mmol, 1 equivalent) in THF (25 mL). Stir at room temperature for 3 h. Add 1N aqueous NaOH and extract the crude with MTBE and discard organics. Add 1N aqueous HCl until acidic pH and extract with EtOAc (x2). Combine organic phases, wash with water and brine, dry over $MgSO_4$, filter and concentrate under reduced pressure to give the title compound (2.5 g, 85%). LCMS (ESI): *m/z* 265.0 $[M+H-BOC]^+$. ^{1}H-NMR (400.13 MHz, $CDCl_3$) δ 8.12-8.01 (m, 2H), 7.55-7.48 (m, 1H), 7.47-7.41 (m, 1H), 3.78-3.43 (m, 2H), 3.32-2.85 (m, 4H), 2.68-2.58 (m, 1H), 2.50-2.35 (m, 1H), 2.06-1.96 (m, 1H), 1.79-1.64 (m, 1H), 1.46 (s, 9H).

tert-Butyl (*R*)-3-((*S*)-1-(*tert*-butoxy)-3-(3-nitrophenyl)-1-oxopropan-2-yl)pyrrolidine-1-carboxylate (**5A**)

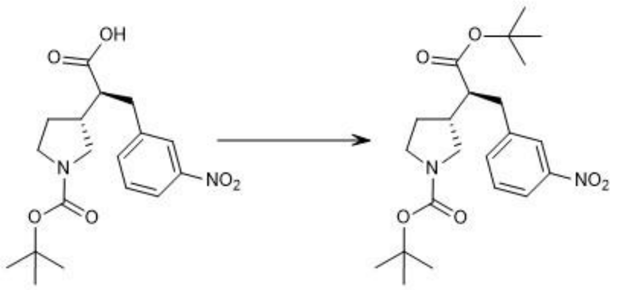

Add 1,1-di-*tert*-butoxy-*N*,*N*-dimethyl-methanamine (18.3 mL, 76.1 mmol, 4 equivalent) to a solution of (*S*)-2-((*R*)-1-(*tert*-butoxycarbonyl)pyrrolidin-3-yl)-3-(3-nitrophenyl)propanoic acid (6.9 g, 19.0 mmol, 1 equivalent) in toluene (190 mL, 10 mL/mmol). Stir and heat the reaction for 6 h at 80 ºC. Add 1,1-di-*tert*-butoxy-*N*,*N*-dimethyl-methanamine (18.3 mL, 76.1 mmol, 4 equivalent) again and stir and heat the reaction overnight at 80 ºC. Concentrate the mixture under reduced pressure. Subject the residue to silica gel chromatography eluting with a 5–20% gradient of EtOAc in hexanes to give the title compound (5.6 g, 70%). LCMS (ESI): *m/z* 309.0 [M+H-2x *tert*-butyl]$^+$. ^{1}H-NMR (400.13 MHz, DMSO-d_6) δ 8.10-8.08 (m, 2H), 7.70 (d, *J* = 7.7 Hz, 1H), 7.59 (t, *J* = 8.1 Hz, 1H), 3.57-3.50 (m, 1H), 3.39-3.34 (m, 1H), 3.22-3.11 (m, 1H), 3.05 (t, *J* = 10.0 Hz, 1H), 2.96-2.84 (m, 2H), 2.64-2.60 (m, 1H), 2.39-2.27 (m, 1H), 1.86 (dd, *J* = 5.6, 11.1 Hz, 1H), 1.67-1.53 (m, 1H), 1.41 (s, 9H), 1.21 (s, 9H).

tert-Butyl (*R*)-3-((*S*)-3-(3-aminophenyl)-1-(*tert*-butoxy)-1-oxopropan-2-yl)pyrrolidine-1-carboxylate (**6**)

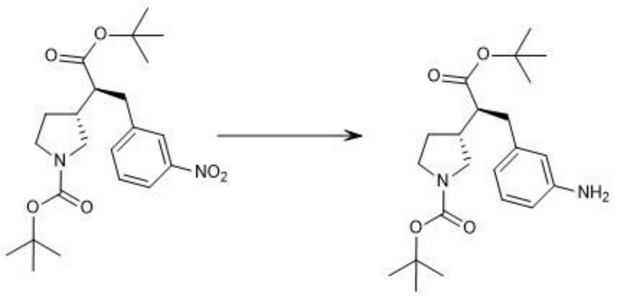

To a stirred solution of *tert*-butyl (*R*)-3-((*S*)-1-(*tert*-butoxy)-3-(3-nitrophenyl)-1-oxopropan-2-yl)pyrrolidine-1-carboxylate (3 g, 7.134 mmol) in EtOAc (0.1 M) under nitrogen atmosphere add palladium on carbon (0.05 equivalent, 0.3567 mmol, 10 mass %). Purge the mixture with hydrogen and stir under a hydrogen atmosphere (balloon) at room temperature overnight. Filter the reaction mixture over a pad of diatomaceous earth. Concentrate the filtrate under reduced pressure to give the title compound (2.62 g, 94%). LCMS (ESI): *m/z* 291.2 $[M+H-BOC]^+$. ^{1}H-NMR (400.13 MHz, $CDCl_3$) δ 7.03 (t, *J* = 7.4 Hz, 1H), 6.59-6.45 (m, 3H), 3.74-3.40 (m, 2H), 3.30-3.17 (m, 1H), 3.07-2.91 (m, 1H), 2.82-2.63 (m, 2H), 2.50-2.29 (m, 2H), 1.99-1.88 (m, 1H), 1.73-1.60 (m, 1H), 1.46 (s, 9H), 1.31 (s, 9H).

di-*tert*-Butyl 3,3'-((2*S*,2'*S*)-((carbonylbis(azanediyl))bis(3,1-phenylene))bis(3-(*tert*-butoxy)-3-oxopropane-1,2-diyl))(3*R*,3'*R*)-bis(pyrrolidine-1-carboxylate) (**7**)

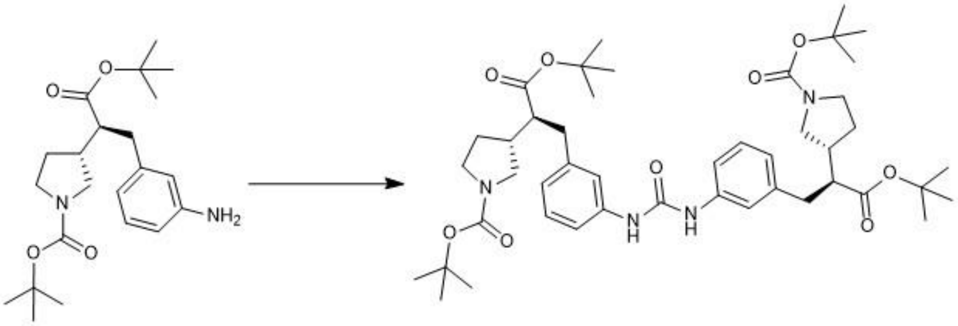

Combine *tert*-butyl (*R*)-3-((*S*)-3-(3-aminophenyl)-1-(*tert*-butoxy)-1-oxopropan-2-yl)pyrrolidine-1-carboxylate (57.80 g, 148.010 mmol, 2 equivalent) and 1,1'-carbonyldiimidazole (12 g, 74 mmol, 1.0 equivalent) in 2-methyl tetrahydrofuran (20 mL/g, 240 mL). Stir and heat the mixture

at 70 ºC overnight. Cool the mixture to room temperature and add water (250 mL) and 2-methyl tetrahydrofuran (250 mL). Separate the layers, discard the aqueous layer, dry the organic layer over $MgSO_4$, filter, and concentrate the filtrate under reduced pressure to provide the crude material. Triturate the crude material at 60 ºC in a mixture of IPA/water 1/1 (600 mL) for 4 h and then at room temperature overnight. Filter to collect the resulting solid, wash the solid with IPA/water 1/1. Dry the solid under reduced pressure at 40 ºC to give the title compound (58 g, 97%). LCMS (ESI): *m/z* 707.2 $[M+H-BOC]^+$. ^{1}H-NMR (400.13 MHz, DMSO-d_6) δ 8.52 (s, 2H), 7.34-7.20 (m, 4H), 7.15 (t, *J*= 7.8 Hz, 2H), 6.7 (d, *J*= 7.3 Hz, 2H), 3.57-3.46 (m, 2H), 3.40-3.27 (m, 2H), 3.22-3.07 (m, 2H), 2.97 (t, *J*= 10.0 Hz, 2H), 2.78-2.62 (m, 4H), 2.52-2.42 (m, 2H), 2.36-2.20 (m, 2H), 1.91-1.79 (m, 2H), 1.68-1.51 (m, 2H), 1.40 (s, 18H), 1.25 (s, 18H).

The diastereomeric excess (de) was determined by HPLC, comparing with the diastereomeric mixture di-*tert*-butyl 3,3'-(((carbonylbis(azanediyl))bis(3,1-phenylene))bis(3-(*tert*-butoxy)-3-oxopropane-1,2-diyl))(3*R*,3'*R*)-bis(pyrrolidine-1-carboxylate) under the assumption that only the stereogenic center in α to the carbonyl will be susceptible to epimerization. SFC analysis (Chiralpak IB 4.6x100mm,5um; gradient from 15 to 55% B in 2 min, hold 1 min at 55% B (A: CO2, B: MeOH-0.2%IPAm), 4 mL/min; 120 bar outlet P/ 40ºC, 220 nm) indicated >98% de: t_R = 0.99 min. The t_R for the other isomers are: t_R = 1.09 min and t_R = 1.16 min. SFC chromatograms included below.

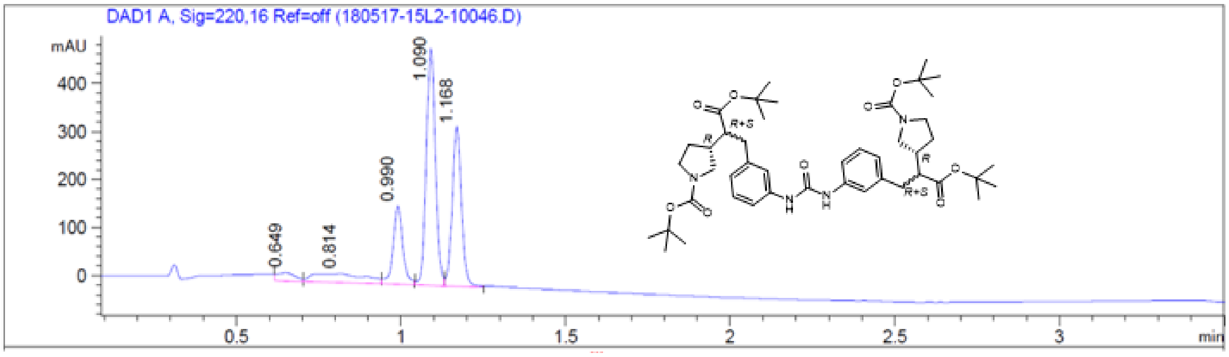

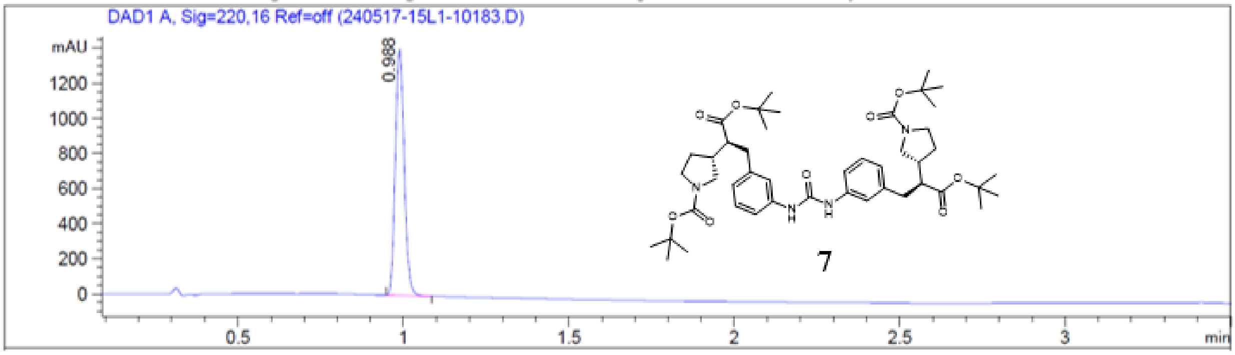

<u>(3*R*,3'*R*)-3,3'-((1*S*,1'*S*)-((Carbonylbis(azanediyl))bis(3,1-phenylene))bis(1-carboxyethane-2,1-diyl))bis(pyrrolidin-1-ium) chloride (**LSN3441732**)</u>

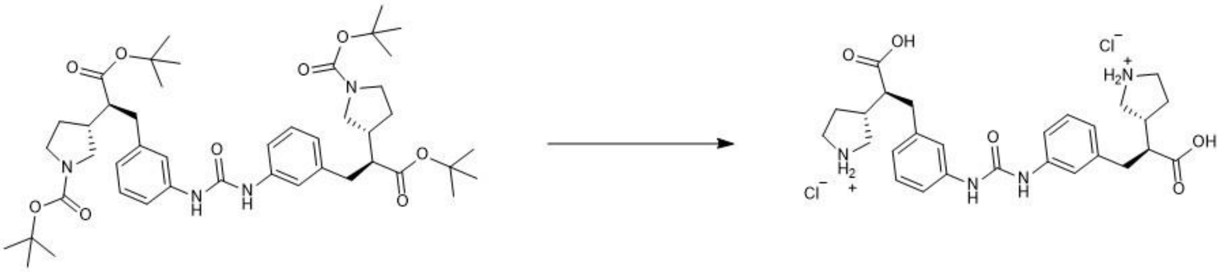

Add HCl solution in dioxane (4.0 mol/l, 170 mL, 693.9 mmol, 10 equivalent) to a mixture of di-*tert*-butyl 3,3'-((2*S*,2'*S*)-((carbonylbis(azanediyl))bis(3,1-phenylene))bis(3-(*tert*-butoxy)-3-oxopropane-1,2-diyl))(3*R*,3'*R*)-bis(pyrrolidine-1-carboxylate) (56 g, 69.39 mmol, 1 equivalent) in 1,4-dioxane (56 mL). Stir the mixture at room temperature overnight. Filter to collect the resulting solid. Wash the solid with dioxane. Dry the resulting solid under reduced pressure at 40 ºC. Triturate the material in *tert*-amyl alcohol (600 mL) overnight. Filter to collect the solid and wash with *tert*-amyl alcohol. Dry the solid under reduced pressure at 40 ºC to give the title compound (39.0 g, 99%). LCMS (ESI): *m/z* 494.8 $[M+H]^+$. ^{1}H-NMR (400.13 MHz, D_2O) δ 7.32 (t, *J* = 7.8 Hz, 2H), 7.66-7.18 (m, 4H), 7.02 (d, *J* = 7.6 Hz, 2H), 3.58 (dd, *J* = 7.9, 11.8 Hz, 2H), 3.48-3.38

(m, 2H), 3.32-3.21 (m, 2H), 3.06 (dd, J = 10.2, 11.6 Hz, 2H), 2.98-2.73 (m, 6H), 2.65-2.50 (m, 2H), 2.26-2.14 (m, 2H), 1.84-1.70 (m, 2H).^{13}C NMR (100.62 MHz, D_2O) δ 177.7, 155.2, 139.3., 138.1, 129.3, 124.1, 120.8, 118.9, 50.2, 48.1, 45.1, 39.5, 36.7, 28.0. HRMS (DART-TOF) *m/z* calculated for C27H34N4O5 [M+H]: 495.2602, found 495.2600. $[\alpha]_D^{23}$ = -33.6 (c = 0.1, H_2O). The diastereomeric excess (de) was measured by HPLC, comparing with the diastereomeric mixture (3*R*,3'*R*)-3,3'-(((carbonylbis(azanediyl))bis(3,1-phenylene))bis(1-carboxyethane-2,1-diyl))bis(pyrrolidin-1-ium) chloride under the assumption that only the stereogenic center in α to the carbonyl will be susceptible to epimerization. HPLC analysis (Column XBridge C18 3.5um, 2.1 x 50mm; gradient mode: from 5 to 95% B in 1.5min, hold 0.5min at 95%B (A: 10mM Ammonium Bicarbonate pH:9.0. B: CH_3CN); 1.2mL/min; 214 nm) indicated >98% de: t_R = 0.41 min. The t_R for the other isomers are: t_R = 0.45 min and t_R = 0.48 min. HPLC chromatograms included below.

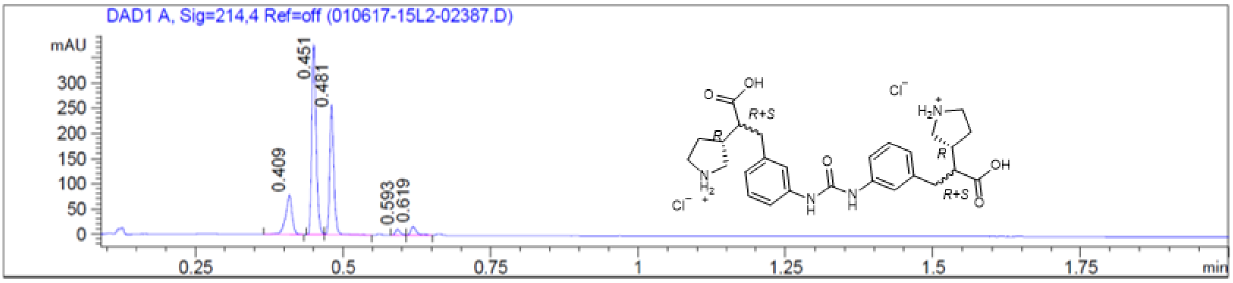

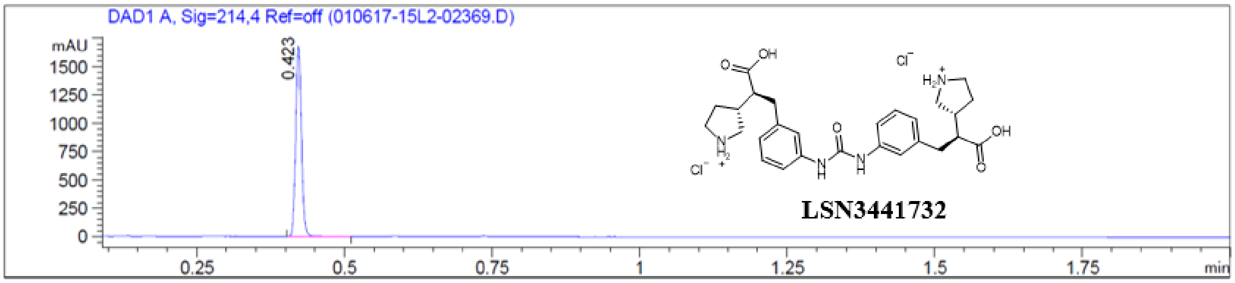

Synthesis of compound LY3473329

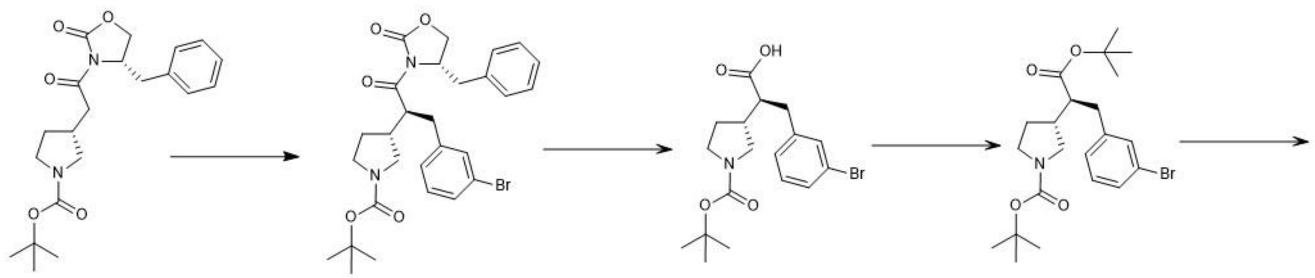

2 **3B** **4B** **5B**

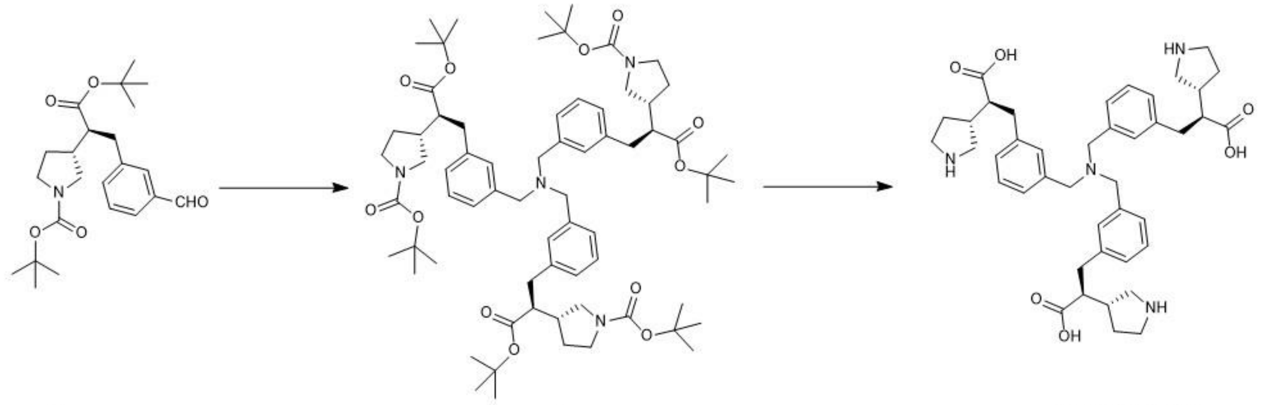

8 **9** **LY3473329**

<u>*tert*-Butyl (*R*)-3-((*S*)-1-((*S*)-4-benzyl-2-oxooxazolidin-3-yl)-3-(3-bromophenyl)-1-oxopropan-2-yl)pyrrolidine-1-carboxylate (**3B**)</u>

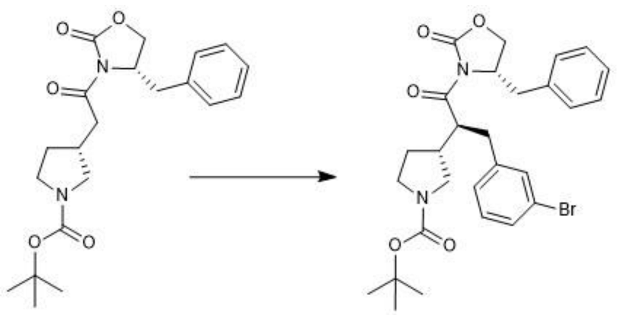

3B was prepared following essentially the same procedure as **3A**, using 1-bromo-3-(bromomethyl)benzene.

LCMS (ESI): *m/z* 501.0/503.0 [M+H-*tert*-butyl]$^+$. ^{1}H-NMR (400.13 MHz, $CDCl_3$) δ 7.43-7.32 (m, 2H), 7.31-7.11 (m, 5H), 7.02 (d, *J* = 6.6 Hz, 2H), 4.66-4.56 (m, 1H), 4.50-4.37 (m, 1H), 4.14-3.96 (m, 2H), 3.78-3.40 (m, 2H), 3.29-3.17 (m, 1H), 3.17-2.86 (m, 4H), 2.60-2.44 (m, 1H), 2.27-2.12 (m, 1H), 1.99-1.89 (m, 1H), 1.80-1.61 (m, 1H), 1.46 (s, 9H).

(2*S*)-3-(3-Bromophenyl)-2-((3*R*)-1-*tert*-butoxycarbonylpyrrolidin-3-yl)propanoic acid (**4B**)

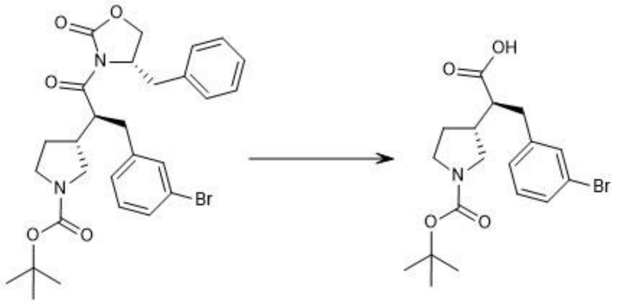

4B was prepared following essentially the same procedure as **4A**.

LCMS (ESI): *m/z* 341.8/343.8 [M+H-*tert*-butyl]$^+$. ^{1}H-NMR (400.13 MHz, $CDCl_3$) δ 7.38-7.29 (m, 2H), 7.19-7.04 (m, 2H), 3.75-3.42 (m, 2H), 3.31-3.19 (m, 1H), 3.12-2.74 (m, 3H), 2.66-2.55 (m, 1H), 2.48-2.31 (m, 1H), 2.05-1.93 (m, 1H), 1.78-1.62 (m, 1H), 1.46 (s, 9H).
The diastereomeric excess was determined to be >95% by NMR spectroscopy as the diastereomeric proton resonances for the minor isomer (which appeared separated from the proton signals of the major isomer) were not observed in the ^{1}H-NMR spectra of the reaction product.

tert-Butyl(*R*)-3-((*S*)-3-(3-bromophenyl)-1-(*tert*-butoxy)-1-oxopropan-2-yl)pyrrolidine-1-carboxylate (**5B**)

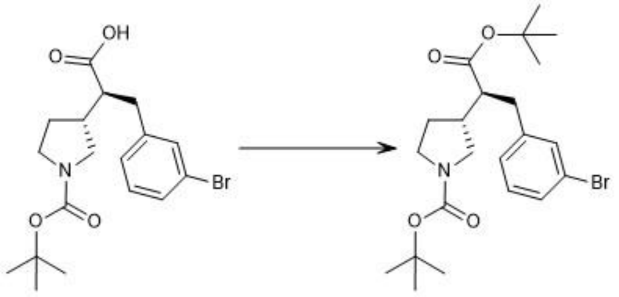

5B was prepared following essentially the same procedure as **5A**.

LCMS (ESI): *m/z* 341.8/343.8 [M+H−2×*tert*-butyl]$^+$. ^{1}H-NMR (400.13 MHz, $CDCl_3$) δ 7.37-7.29 (m, 2H), 7.18-7.05 (m, 2H), 3.75-3.39 (m, 2H), 3.32-3.18 (m, 1H), 3.10-2.90 (m, 1H), 2.89-2.69 (m, 2H), 2.50-2.29 (m, 2H), 2.00-1.89 (m, 1H), 1.75-1.58 (m, 1H), 1.47 (s, 9H), 1.30 (s, 9H).

tert-Butyl(*R*)-3-((*S*)-1-(*tert*-butoxy)-3-(3-formylphenyl)-1-oxopropan-2-yl)pyrrolidine-1-carboxylate (**8**)

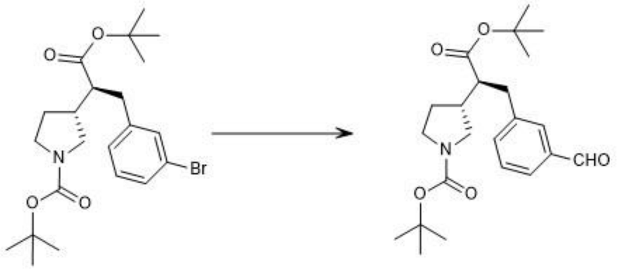

Add *tert*-butyl (*R*)-3-((*S*)-3-(3-bromophenyl)-1-(*tert*-butoxy)-1-oxopropan-2-yl)pyrrolidine-1-carboxylate (48 g, 105.6 mmol, 1.0 equivalent), degassed toluene (0.12 M, 880 mL), *n*-butyldi-1-adamantylphosphine (0.1 mol/mol, 10.56 mmol, 95 mass %, 3.987 g, 0.1 equivalent), palladium(II) acetate (0.05 mol/mol, 5.282 mmol, 1.186 g, 0.05 equivalent) and *N*,*N*,*N'*,*N'*-tetramethylethylenediamine (1.5 equivalent, 158.5 mmol, 23.9 mL, 1.5 equivalent) to a 2L Parr reactor. Pressurize the Parr reactor with 70 psi CO/H_2 1:1. Stir the mixture at 100 ºC overnight. Cool the mixture to room temperature and evaporate the solvent to dryness. Add water (500 mL) to the crude and extract the mixture with EtOAc (500 mL). Wash organic layer with 1M aqueous $KHSO_4$ (200 mL), dry over $MgSO_4$, filter through silica gel and concentrate to dryness to afford the title compound (42 g, 98% yield) as a yellow oil. LCMS (ESI): *m/z* 426.0 [M+Na]. ^{1}H-NMR (400.13 MHz, $CDCl_3$) δ 9.98 (s, 1H), 7.76-7.67 (m, 2H), 7.48-7.41 (m, 2H), 3.79-3.41 (m, 2H), 3.33-3.20 (m, 1H), 3.11-2.80 (m, 3H), 2.57-2.47 (m, 1H), 2.45-2.31 (m, 1H), 2.00-1.91 (m, 1H), 1.76-1.56 (m, 1H), 1.47 (s, 9H), 1.25 (s, 9H).

tri-*tert*-Butyl 3,3',3''-((2*S*,2'*S*,2''*S*)-((nitrilotris(methylene))tris(benzene-3,1-diyl))tris(3-(*tert*-butoxy)-3-oxopropane-1,2-diyl))(3*R*,3'*R*,3''*R*)-tris(pyrrolidine-1-carboxylate) (**9**)

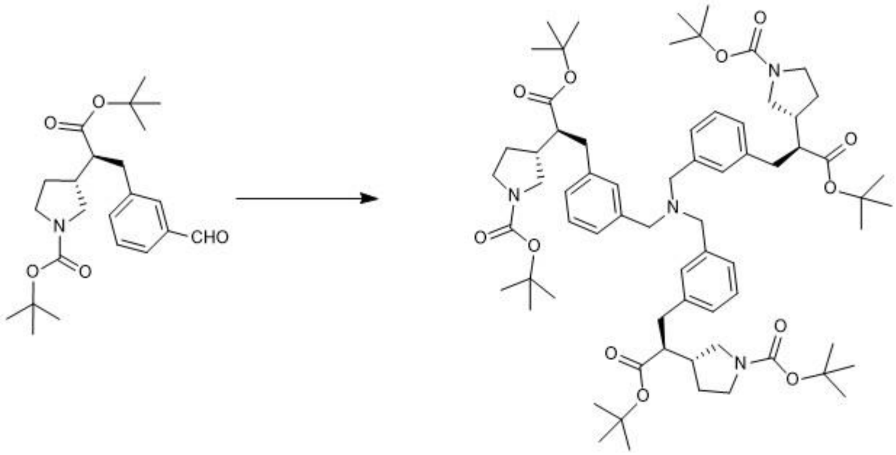

Add *tert*-butyl (*R*)-3-((*S*)-1-(*tert*-butoxy)-3-(3-formylphenyl)-1-oxopropan-2-yl)pyrrolidine-1-carboxylate (222 g, 550.2 mmol, 1.0 equivalent), 2-propanol (4 mL/g, 888 mL), ammonia (2 mol/L in 2-propanol, 302.6 mL, 1.1 equivalent) to a round bottom flask. Cool the mixture to 0–5 ºC with an ice water bath. Add sodium triacetoxyborohydride (550.2 mmol, 1.0 equivalent) in four portions at 40 min intervals. Stir the mixture at room temperature overnight. Evaporate the solvent to dryness. Add water (200 mL) and aqueous 2M K_2HPO_4 (300 mL) and extract with MTBE (2 × 500 mL). Dry the organic layer over $MgSO_4$, filter, and concentrate under reduced pressure. Subject the residue to silica gel chromatography eluting with a 20–80% gradient of EtOAc in hexanes to give the title compound (57.8 g, 27%). ^{1}H-NMR (400.13 MHz, $CDCl_3$) δ 7.30-7.18 (m, 6H), 7.14-7.06 (m, 3H), 7.5-6.98 (m, 3H), 3.75-3.34 (m, 12H), 3.30-3.17 (m, 3H), 3.10-2.92 (m, 3H), 2.89-2.70 (m, 6H), 2.55-2.28 (m, 6H), 1.98- 1.88 (m, 3H), 1.74-1.57 (m, 3H), 1.45 (s, 27H), 1.20 (s, 27H).

(2*S*,2'*S*,2''*S*)-3,3',3''-((Nitrilotris(methylene))tris(benzene-3,1-diyl))tris(2-((*R*)-pyrrolidin-3-yl)propanoic acid) (**LY3473329**)

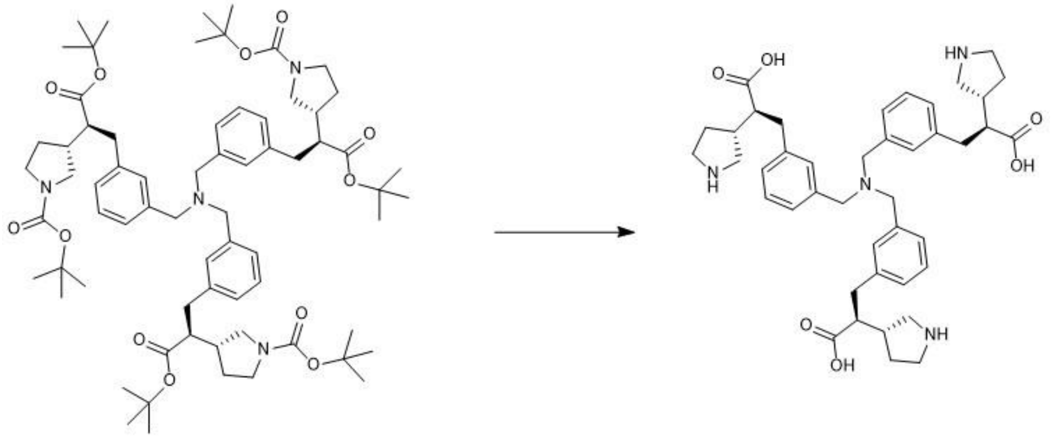

To a round bottom flask, add tri-*tert*-butyl 3,3',3''-((2*S*,2'*S*,2''*S*)-((nitrilotris(methylene))tris(benzene-3,1-diyl))tris(3-(*tert*-butoxy)-3-oxopropane-1,2-diyl))(3*R*,3'*R*,3''*R*)-tris(pyrrolidine-1-carboxylate) (499.3 g, 423.3 mmol), 1,4-dioxane (1,997 mL), and a solution of hydrochloric acid (12 M in water, 529.1 mL, 15 equivalent). Stir the mixture at 40 ºC for 1 h and then concentrate the mixture under reduced pressure to remove 1,4-dioxane, resulting in an aqueous slurry. Filter the mixture through a propylene filter to eliminate insoluble particles. Adjust the pH of the filtrate to 9–10 using a solution of NaOH (2M in water). Stir the mixture at room temperature overnight. Collect the resulting solid using a paper filter. Wash the solid with water and dry under reduced pressure at 45 ºC to obtain the title compound (281 g, 88%) as a white crystalline solid.

LCMS (ESI): *m/z* 711.4 $[M+H]^+$. ^{1}H-NMR (400.13 MHz, D_2O + 1% DCl) δ 7.36-7.25 (m, 6H), 7.15-7.09 (m, 6H), 4.28-4.15 (m, 6H), 3.51 (dd, *J*= 7.9, 11.6 Hz, 3H), 3.37-3.28 (m, 3H), 3.21-3.10 (m, 3H), 2.99 (dd, *J*= 10.1, 11.6 Hz, 3H), 2.89 (dd, *J*= 4.5, 13.1 Hz, 3H), 2.84-2.68 (m, 6H), 2.59-2.45 (m, 3H), 2.17-2.05 (m, 3H), 1.77-1.63 (m, 3H). ^{13}C NMR (100.62 MHz, D_2O + 1% DCl) δ 177.6, 139.4, 131.7, 130.5, 129.6, 129.5, 129.4, 57.3, 50.4, 48.1, 45.2, 39.6, 36.5, 28.1. HRMS (DART-TOF) *m/z* calculated for C42H54N4O6 [M+H]: 711.4116, found 711.4109. $[\alpha]_D^{23}$ = -34.7 (c = 0.1, H_2O). The diastereomeric excess (de) was measured by HPLC, comparing with the diastereomeric mixture 3,3',3''-((nitrilotris(methylene))tris(benzene-3,1-diyl))tris(2-((*R*)-pyrrolidin-3-yl)propanoic acid) under the assumption that only the stereogenic center in α to the carbonyl will be susceptible to epimerization. HPLC analysis (Column XBridge C18 3.5um, 2.1 x 50mm; gradient mode: from 10 to 100% B in 3 min; hold 0.75min at 100%B (A: 10mM Ammonium Bicarbonate pH:9.0, B: CH_3CN); 1.0mL/min; 214 nm) indicated >98% de: t_R = 0.67

min. The t_R for the other isomers are: t_R = 0.71 min, t_R = 0.75 min, and t_R = 0.79 min. HPLC chromatograms included below.

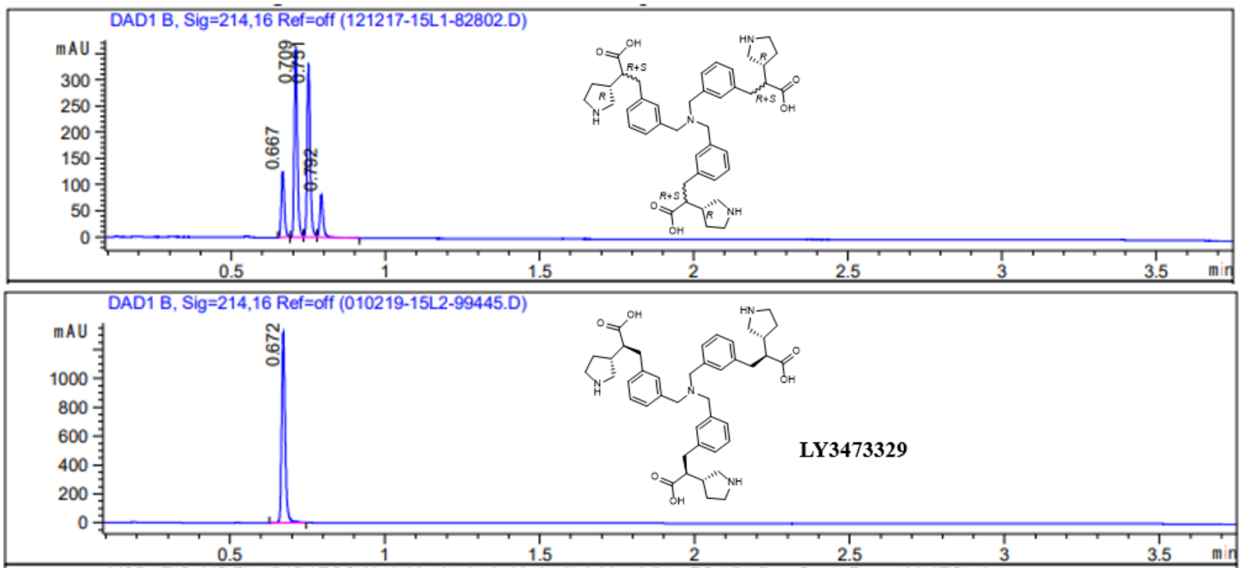

HPLC analysis (Agilent InfinityLab Poroshell 120 Chiral-V, 150x4.6 mm, 2.7 mm P/N:683975-604; gradient mode: 70A:30B Isocratic (A: 20 mM ammonium formate in water (pH=4.0±0.2), B: 60 MeOH:40 CH_3CN); 0.7 mL/min, 200 nm) indicated >99% ee and de: t_R (major enantiomer)= 16.79 min, t_R (minor enantiomer)=13.94 min, t_R (diasteroisomer)= 9.16. % Chiral impurities (%w/w) (sum of enantiomer/diastereoisomer)= 0.1%

Typical Chromatogram of Marker Solution

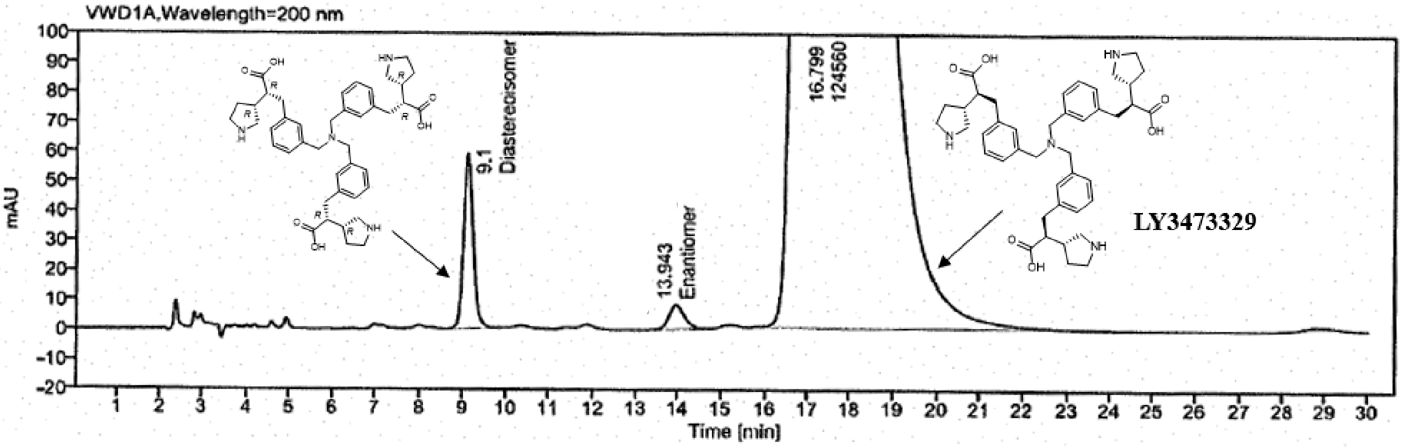

Typical Chromatogram of Sample Solution

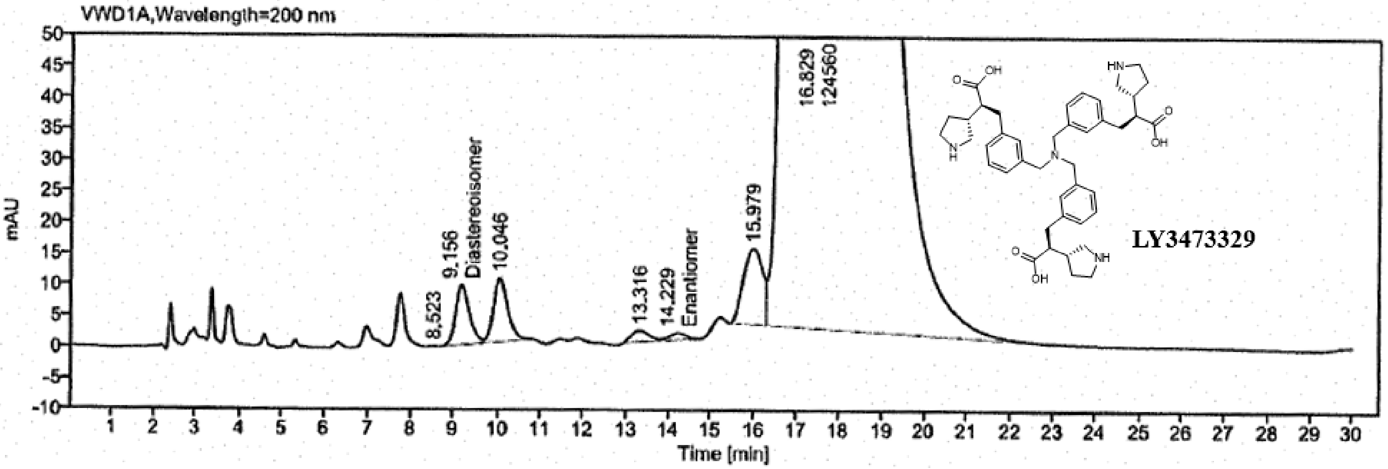

Synthesis of compound LSN3353871

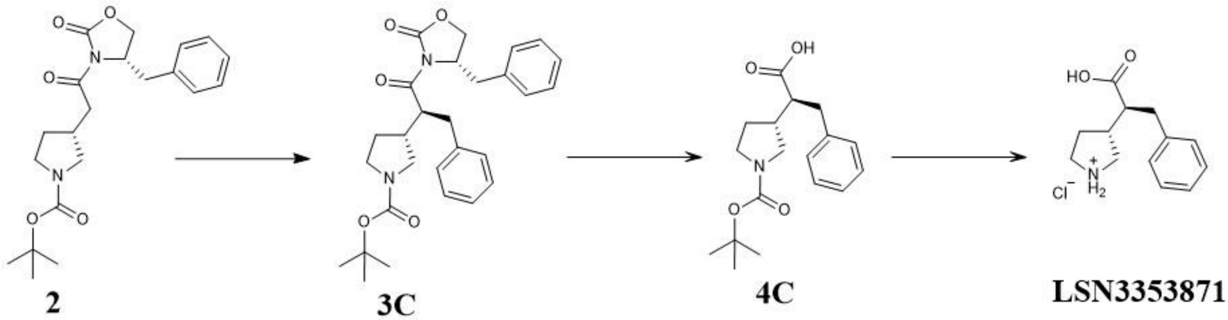

2 3C 4C LSN3353871

tert-Butyl (*R*)-3-((*S*)-1-((*S*)-4-benzyl-2-oxooxazolidin-3-yl)-1-oxo-3-phenylpropan-2-yl)pyrrolidine-1-carboxylate (**3C**)

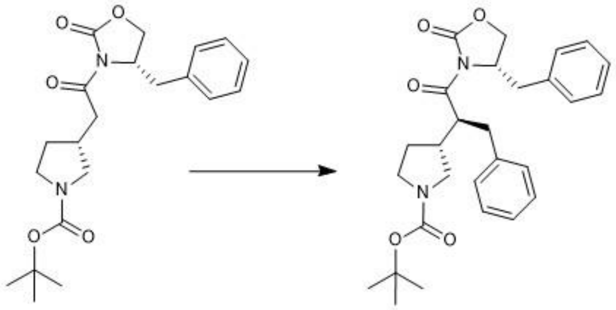

3C is prepared following essentially the same procedure as **3A**, using (bromomethyl)benzene.

LCMS (ESI): *m/z* 423.2 [M+H-*tert*-butyl]$^+$.

(*S*)-2-((*R*)-1-(*tert*-Butoxycarbonyl)pyrrolidin-3-yl)-3-phenylpropanoic acid (**4C**)

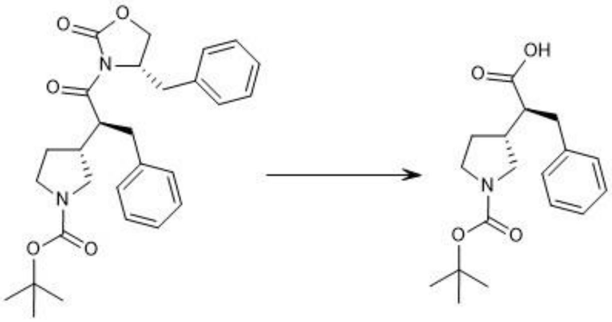

4C is prepared following essentially the same procedure as **4A**.

LCMS (ESI): *m/z* 264.2 [M + H-*tert*-butyl]$^{+}$. ^{1}H-NMR (400.13 MHz, $CDCl_3$) δ 7.33-7.07 (m, 5H), 3.77-3.40 (m, 2H), 3.30-3.16 (m, 1H), 3.12-2.77 (m, 3H), 2.71-2.54 (m, 1H), 2.47-2.30 (m, 1H), 2.06-1.90 (m, 1H), 1.77-1.57 (m, 1H), 1.45 (s, 9H).

HPLC (Chiralcel OJ 4.6 x 250mm, 10um; gradient mode: 7% B Isocratic mode (A: Hexane-TFA 0.05%, B: EtOH; 1 mL/min, 215 nm) indicates de and ee >98%. HPLC chromatograms included below.

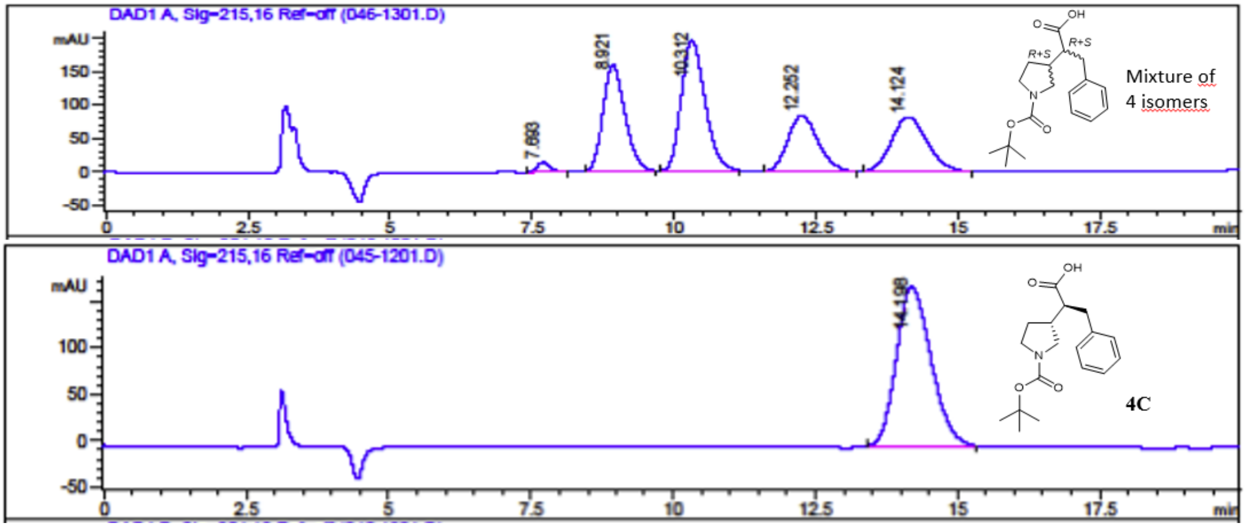

(*R*)-3-((*S*)-1-Carboxy-2-phenylethyl)pyrrolidin-1-ium chloride (**LSN3353871**)

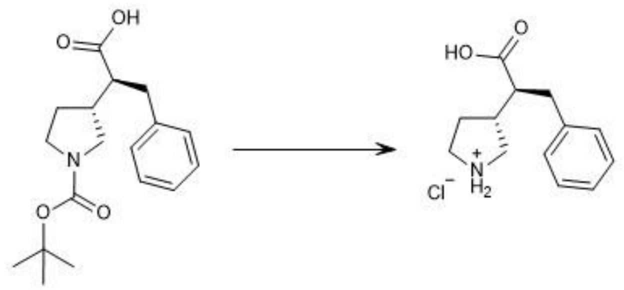

BOC deprotection is carried out following essentially the same procedure as described for the synthesis of **LSN3441732**.

LCMS (ESI): *m/z* 220.2 $[M + H]^+$. ^{1}H NMR (400.13 MHz, D_2O) δ 7.40 – 7.34 (m, 2H), 7.33 – 7.24 (m, 3H), 3.59 (dd, *J* = 11.8, 7.9 Hz, 1H), 3.48-3.38 (m, 1H), 3.33-3.20 (m, 1H), 3.07 (dd, *J* = 11.8, 10.0 Hz, 1H), 2.99 – 2.84 (m, 3H), 2.66-2.51 (m, 1H), 2.25-2.13 (m, 1H), 1.84-1.70 (m, 1H).^{13}C NMR (100.62 MHz, D_2O) δ 178.0, 138.3, 128.9, 128.8, 127.0, 50.5, 48.2, 45.2, 39.5, 36.8, 28.2. HRMS (DART-TOF) *m/z* calculated for C13H17NO2 [M+H]: 220.1332, found 220.1317. $[\alpha]_D^{23}$ = -35.1 (c = 0.1, H_2O). The diastereomeric excess was determined to be >95% by NMR spectroscopy as the diastereomeric proton resonances of the minor isomer that appeared separated from the proton signals of the major isomer were not observed in the ^{1}H-NMR spectra of the reaction product.

Synthesis of compound LSN3374443

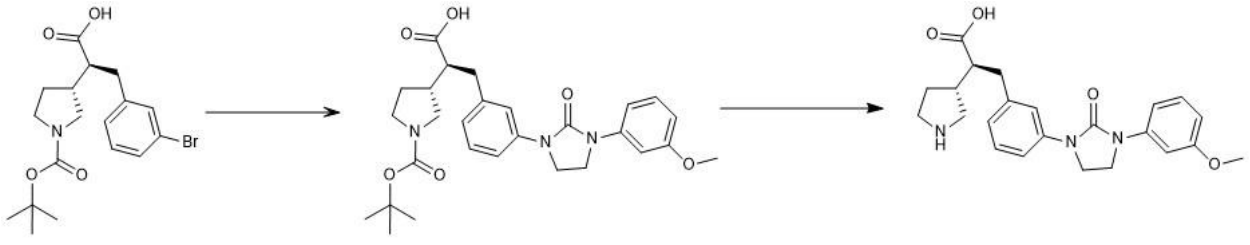

4B 10 LSN3374443

<u>(*S*)-2-((*R*)-1-(*tert*-Butoxycarbonyl)pyrrolidin-3-yl)-3-(3-(3-(3-methoxyphenyl)-2-oxoimidazolidin-1-yl)phenyl)propanoic acid (**10**)</u>

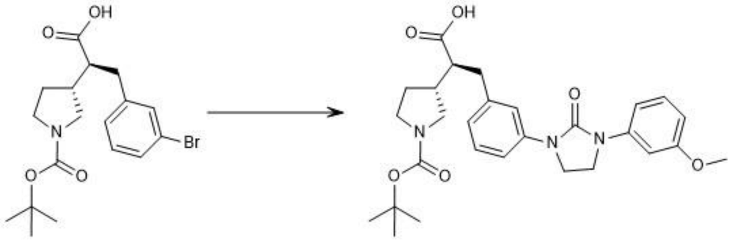

Mix (*S*)-3-(3-bromophenyl)-2-((*R*)-1-(tert-butoxycarbonyl)pyrrolidin-3-yl)propanoic acid (190 mg, 0.4770 mmol), 1-(3-methoxyphenyl)imidazolidin-2-one (0.2751 g, 1.431 mmol, 3 equivalent), [(2-di-*tert*-butylphosphino-2',4',6'-triisopropyl-l,1'-biphenyl)-2-(2'-amino-l,1'-biphenyl)]palladium(ll) methanesulfonate (*t*BuXPhos-Pd-G3, 0.0379 g, 0.0477 mmol, 0.1 equivalent) and sodium *tert*-butoxide (0.1418 g, 1.431 mmol, 3 equivalent) in 1,4-dioxane (4.8 mL) and stir the mixture under a nitrogen atmosphere at 100 ºC overnight. Dilute the mixture with EtOAc and acidify with an aqueous solution of HCl (1N). Filter the mixture through a pad of diatomaceous earth, separate the layers, and dry the organics over $MgSO_4$. Filter and concentrate the organics, then purify the residue by reverse-phase flash chromatography (silica-bound C18 column) using a gradient of 40–70% acetonitrile in aqueous NH_4CO_3 (pH 9) to give the title compound (70 mg, 28%) as a white solid. LCMS (ESI): *m/z* 510.2 $[M+H]^+$. ^{1}H-NMR (400.13 MHz, DMSO-d_6) δ 7.50-7.44 (m, 2H), 7.32 (t, *J* = 2.2 Hz, 1H), 7.30-7.23 (m, 2H), 7.17-7.13 (m, 1H), 6.91 (d, *J* = 7.6 Hz, 1H), 6.68-6.64 (m, 1H), 3.97-3.93 (m, 4H), 3.77 (s, 3H), 3.56-3.44 (m,

1H), 3.21-3.07 (m, 1H), 3.01-2.91 (m, 1H), 2.86-2.71 (m, 2H), 2.60-5.53 (m, 2H), 2.34-2.23 (m, 1H), 1.92-1.82 (m, 1H), 1.69-1.53 (m, 1H), 1.40 (s, 9H).

(*S*)-3-(3-(3-(3-Methoxyphenyl)-2-oxoimidazolidin-1-yl)phenyl)-2-((*R*)-pyrrolidin-3-yl)propanoic acid (**LSN3374443**)

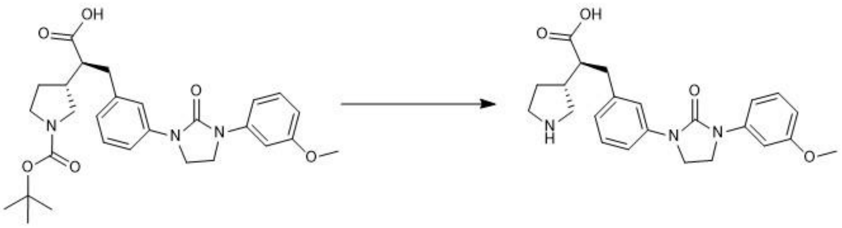

BOC deprotection is carried following essentially the same procedure as described for the synthesis of LSN3441732. Purify by reverse-phase flash chromatography (silica-bound C18 column) using a gradient of 5–50% acetonitrile in aqueous NH_4CO_3 (pH 9) to give the title compound.

LCMS (ESI): *m/z* 410.2 $[M+H]^+$. ^{1}H NMR (400.13 MHz, D_2O + 1% DCl) δ 7.14 – 7.05 (m, 4H), 6.95 (t, *J* = 2.3 Hz, 1H), 6.79 (ddd, *J* = 12.8, 7.9, 3.0 Hz, 2H), 6.49 (dd, *J* = 8.3, 2.4 Hz, 1H), 3.55 (s, 3H), 3.43-3.33 (m, 6H), 3.16 (ddd, *J* = 11.7, 9.8, 7.2 Hz, 1H), 2.91 (dd, *J* = 11.8, 10.0 Hz, 1H), 2.65-2.63 (m, 3H), 2.43 (m, 1H), 2.10-2.03 (m, 1H), 1.66 (m, 1H).^{13}C NMR (100.62 MHz, D_2O + 1% DCl) δ 177.4, 159.2, 155.6, 140.6, 139.4, 139.1, 129.8, 129.1, 123.7, 118.6, 116.7, 110.8, 108.5, 104.3, 55.1, 50.1, 48.0, 45.2, 41.8, 41.7, 39.6, 36.8, 28.0. HRMS (DART-TOF) *m/z* calculated for C23H27N3O4 [M+H]: 410.2074, found 410.2053.

Synthesis of compound ^{3}H-LSN3441732

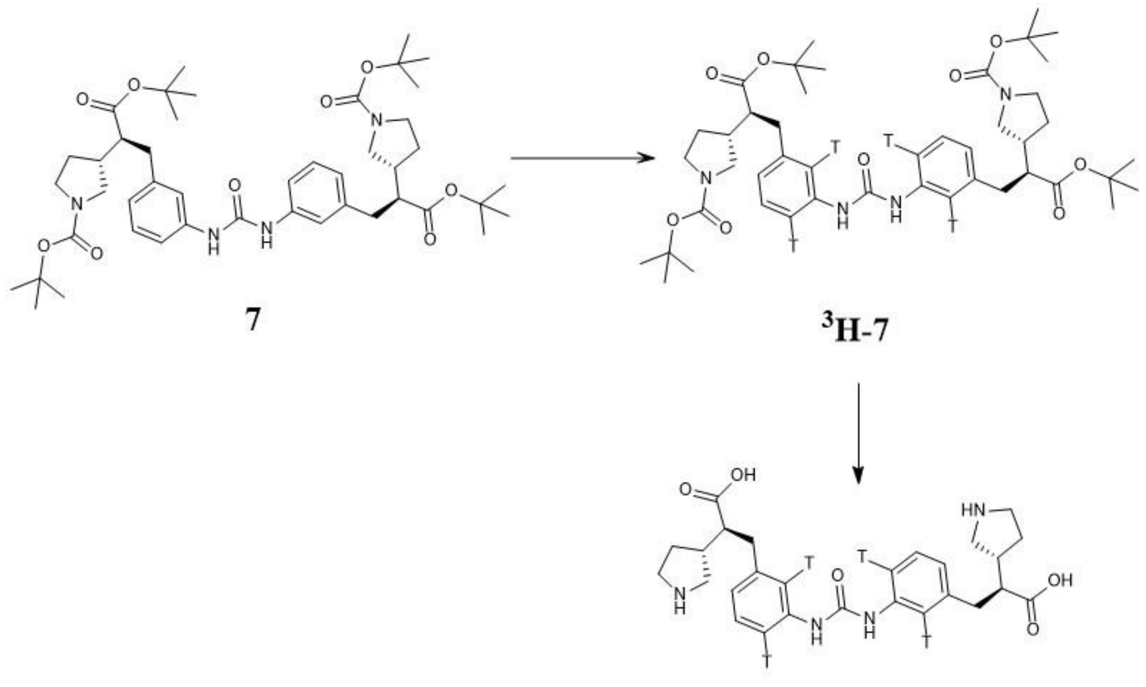

^{3}H-LSN3441732

di-*tert*-Butyl 3,3'-((2*S*,2'*S*)-((carbonylbis(azanediyl))bis(3,1-phenylene-2,4-t_2))bis(3-(tert-butoxy)-3-oxopropane-1,2-diyl))(3*R*,3'*R*)-bis(pyrrolidine-1-carboxylate) (^{3}**H-7**)

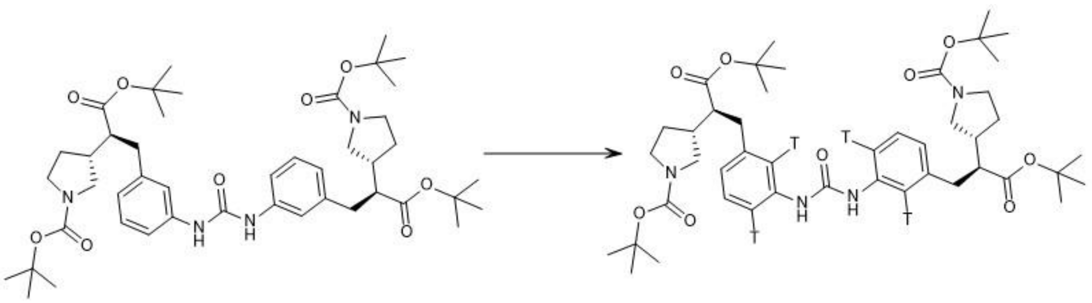

Combine **7** (5 mg), Crabtree’s catalyst (10 mg), and dichloromethane (1 mL) in a tritiation flask and stir under 5 Ci of tritium gas for 16 h. Remove labile tritium by repeated evaporations to dryness from ethanol. Dissolve the residues in ethanol.

(2*S*,2’*S*)-3,3’-((Carbonylbis(azanediyl))bis(3,1-phenylene-2,4-t_2))bis(2-((*R*)-pyrrolidin-3-yl)propanoicacid) (**^{3}H-LSN3441732**)

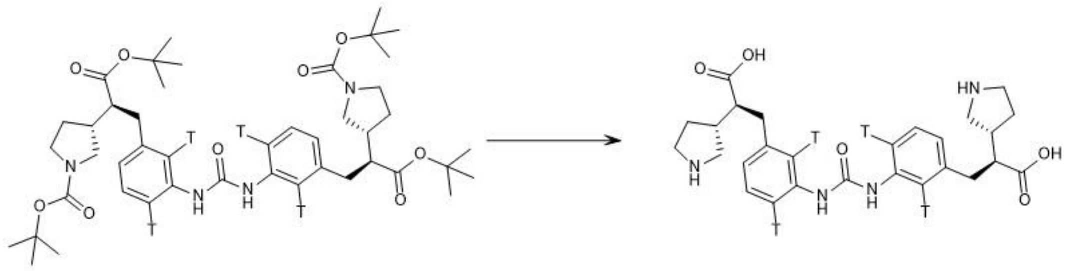

Dry ^{3}H-**7** to dryness in a small round bottom flask. Add 2-methyl-2-butanol (2 mL). Stir the slurry and add 4M hydrochloric acid in dioxane (1 mL) was added dropwise under a nitrogen atmosphere. The resulting solution is stirred at 45 °C for 4 h then overnight at room temperature.

Crude ^{3}H-LSN3441732 was purified by HPLC as follows:

Column: Gemini C18 25 × 1 cm

Eluent A: water + 0.1% TFA

Eluent B: acetonitrile + 0.1% TFA

Gradient: 5–100% B over 2 h

Flow: 3 mL/min

^{3}H-LSN3441732 was collected, rotary evaporated to dryness, and dissolved in ethanol.

The radiochemical purity of ^{3}H-LSN3441732, determined by high-performance liquid chromatography was 98%. Mass spectrometry of ^{3}H-LSN3441732 gave a spectrum that was consistent with the inactive material LSN3441732 and a specific activity of 40 Ci/mmol. Tritium NMR spectroscopy of ^{3}H-LSN3441732 was consistent with the structure. Assignment of the tritium NMR is based on the results of a deuteration experiment in the protected intermediate **7**.

Spectra of the compounds described

LSN 3441732 (^1H NMR in D_2O)

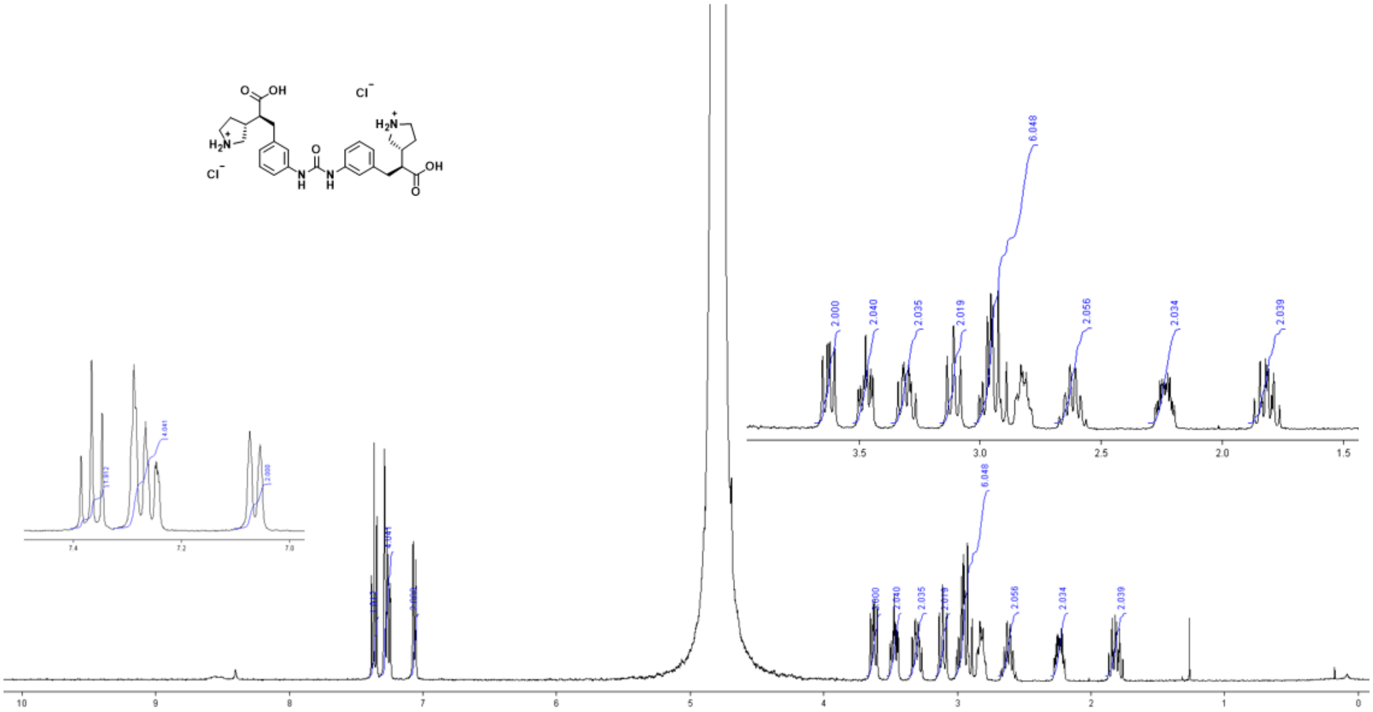

LSN 3441732 (^{13}C NMR in D_2O)

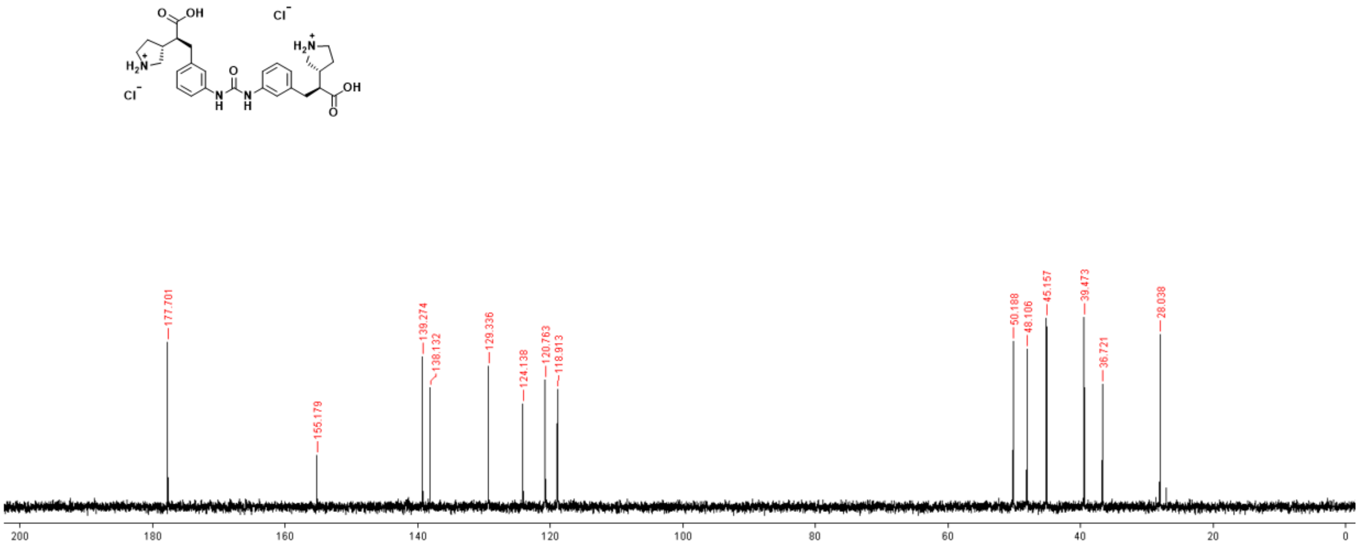

FILE NAME: D:\DATOS\2017\15L\050617-15L2-02481.D
INJ. METHOD: C:\Chem32\1\METHODS\NEW_OA\HIGHPH.M Vial: P1-B-09
Method info: Column XBridge C18 3.5um, 2.1 x 50mm; UV: 214 and 300; MS-ESI
Alcobendas AT 100-800 A: 10mM Ammonium Bicarbonate ph:9.0. B: CH3CN
Flow Rate: 1.2ml/min; Tª 50ºC; Gradient mode: From 5 to 95% B
in 1.5min. Hold 0.5min at 95%B.

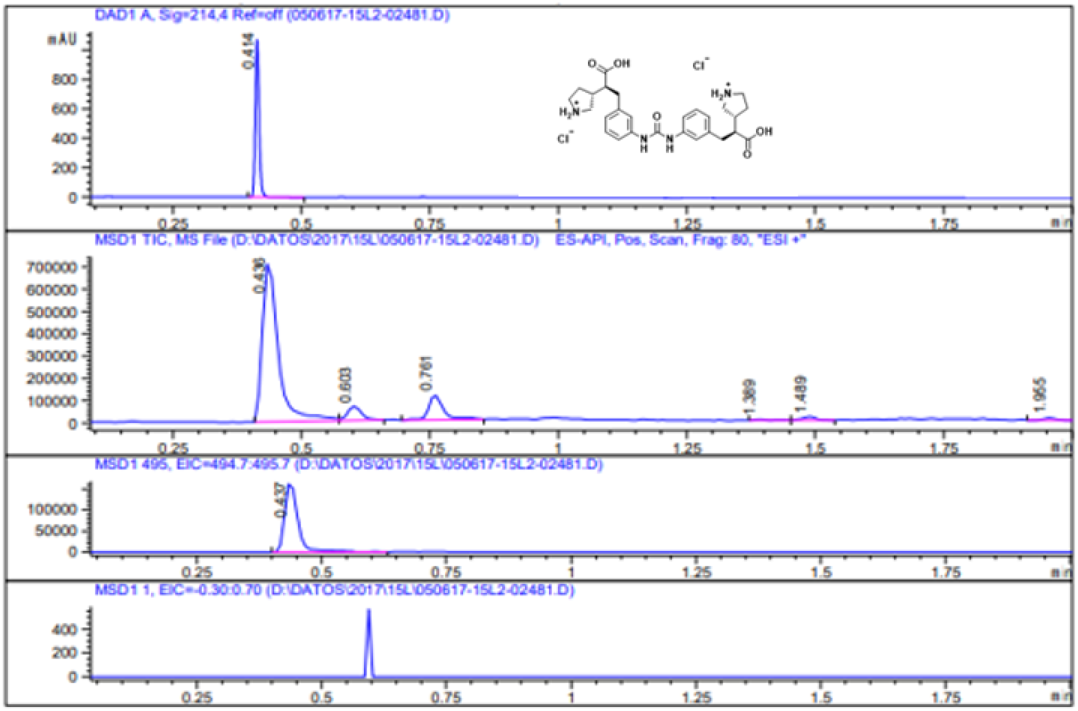

Area % Table of Signal DAD1 A, Sig=214,4

RET.T	Height	Area	Area%
0.414	982	567	100.00

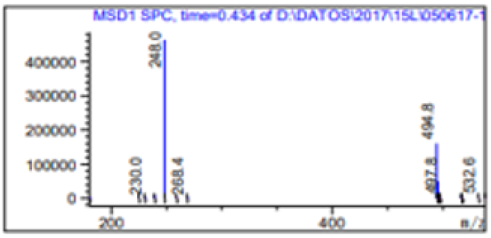

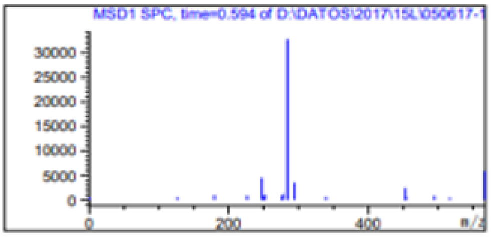

Target 1 : 494.00
UV RT T1 : 0.414
Purity T1 : 100.00
PosThr T1 : 167453

High Resolution Mass Spectrometry Report: LSN3441732

Acquisition method: Column: X-Bridge C18 3.5µM 2.1x50mm. Mobile phase: A= 0.1% FA in water; B= 0.1% FA in CH_3CN. Run length: 2 min. Column temperature: 50ºC. Acquisition mode: ES+. Injection volume: 1µL

Time [min]	A [%]	B [%]	Flow [mL/min]	Max. Pressure Limit [bar]
0.00	95.00	5.00	1.200	1000.00
0.25	95.00	5.00	---	---
1.25	5.00	95.00	---	---
1.75	5.00	95.00	---	---
2.00	95.00	5.00	---	---

Ref. Standards from Agilent's Internal Reference Mass Kit (p/ G1969-85001)

- Purine (m/z 121.05087)
- HP-921 (m/z 922.00980)

**Sample**	
LSN	**3441732**
Molecular Formula	C27H34N4O5
Exact Mass	494.2529
Exact Mass (M+H)	495.2602
**Experimental Results**	
**Measured Exact Mass of $(M+H)^+$**	**495.2600**
ppm error with expected Exact Mass:	**-0.36**

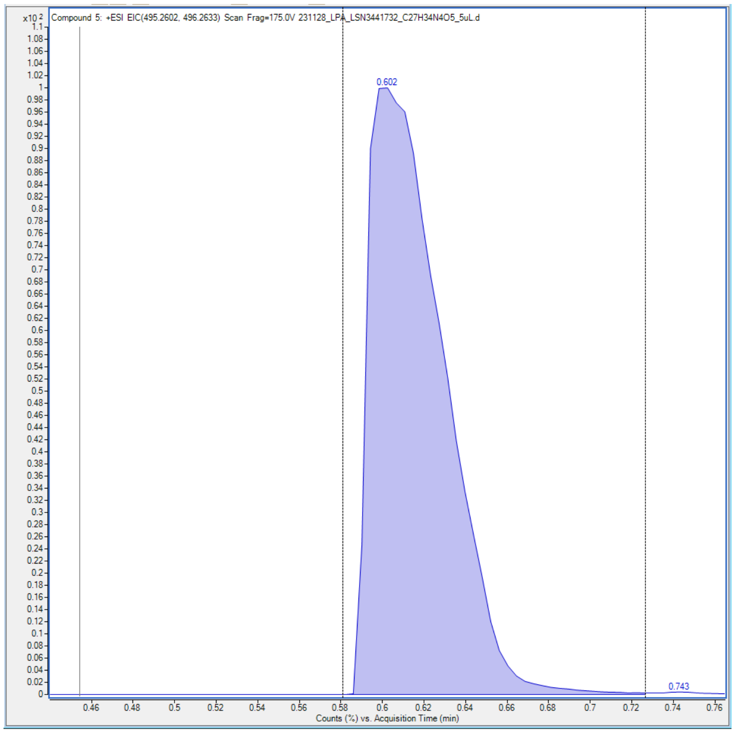
Compound 5: +ESI EIC(495.2602, 496.2633) Scan Frag=175.0V 231128_LPA_LSN3441732_C27H34N4O5_5uL.d
0.602
0.743
Counts (%) vs. Acquisition Time (min)

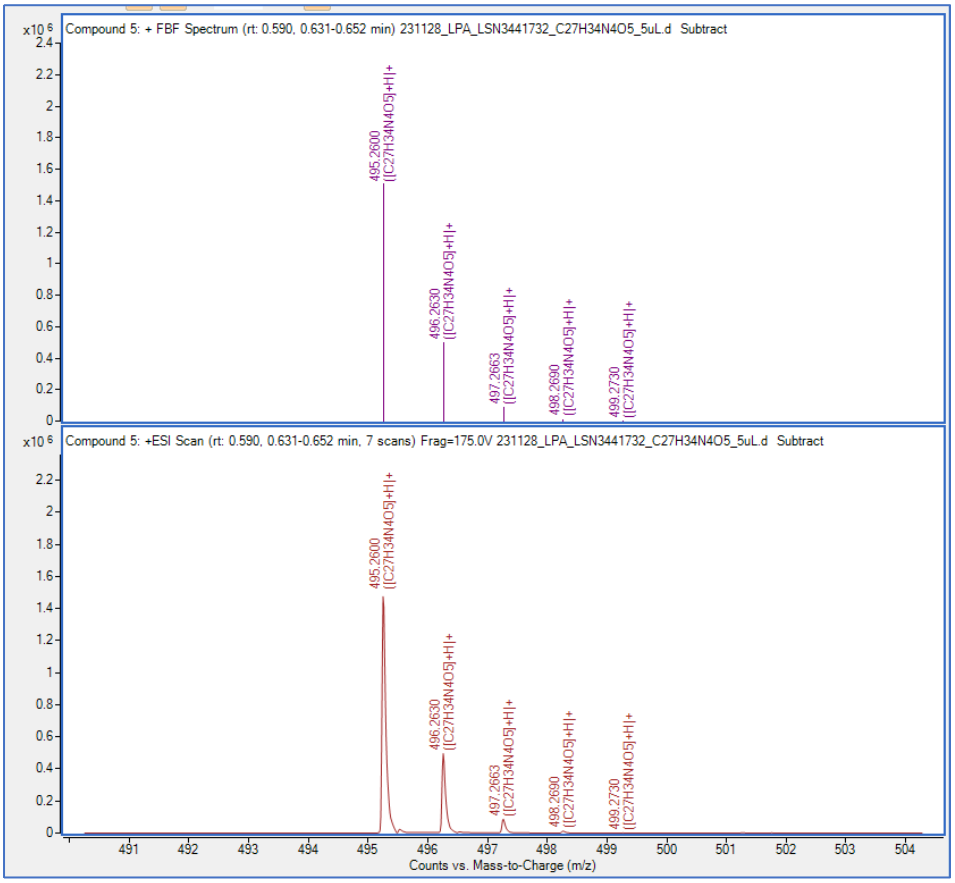
Compound 5: + FBF Spectrum (rt: 0.590, 0.631-0.652 min) 231128_LPA_LSN3441732_C27H34N4O5_5uL.d Subtract
495.2600 ([C27H34N4O5]+H]+
496.2630 ([C27H34N4O5]+H]+
497.2663 ([C27H34N4O5]+H]+
498.2690 ([C27H34N4O5]+H]+
499.2730 ([C27H34N4O5]+H]+
Compound 5: +ESI Scan (rt: 0.590, 0.631-0.652 min, 7 scans) Frag=175.0V 231128_LPA_LSN3441732_C27H34N4O5_5uL.d Subtract
495.2600 ([C27H34N4O5]+H]+
496.2630 ([C27H34N4O5]+H]+
497.2663 ([C27H34N4O5]+H]+
498.2690 ([C27H34N4O5]+H]+
499.2730 ([C27H34N4O5]+H]+
Counts vs. Mass-to-Charge (m/z)

LY 3473329 (^{1}H NMR in D_2O + 1% DCl)

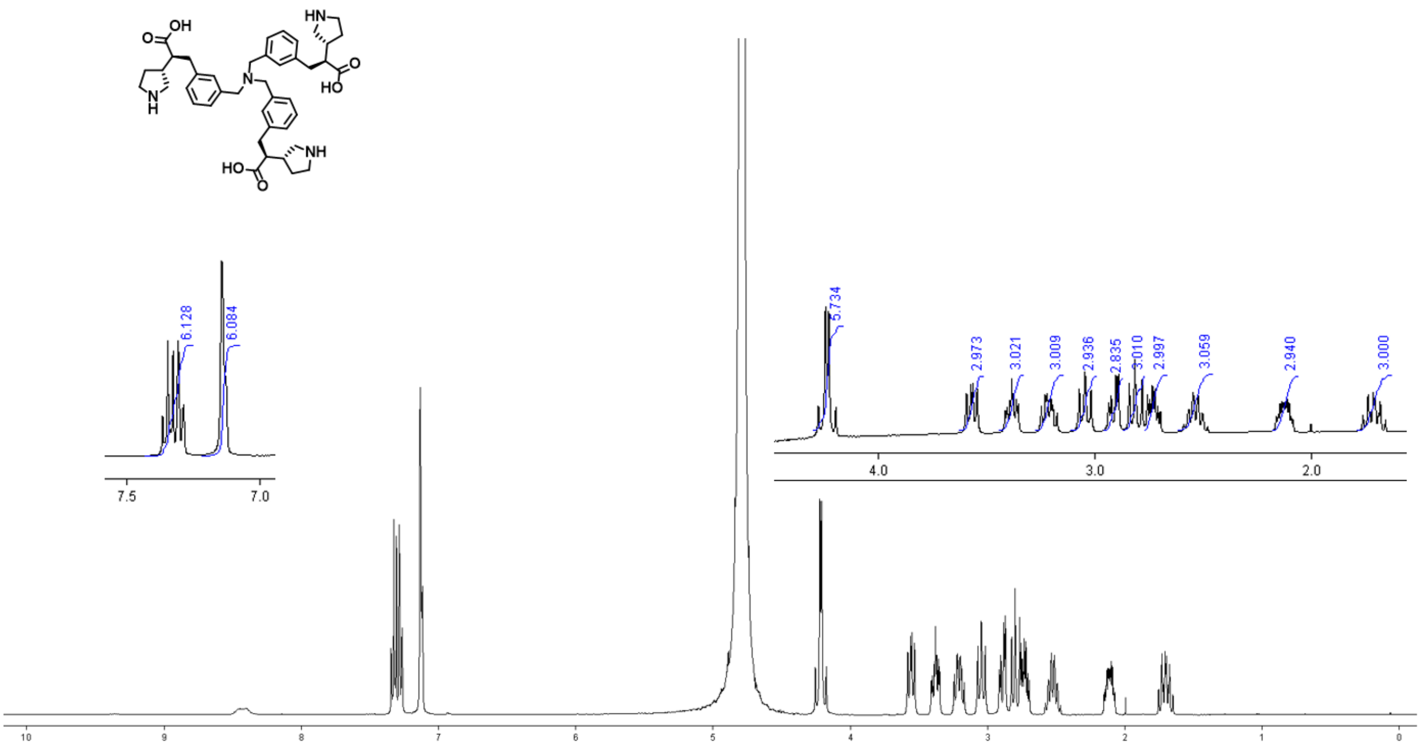

LY 3473329 (^{13}C NMR in D_2O + 1% DCl)

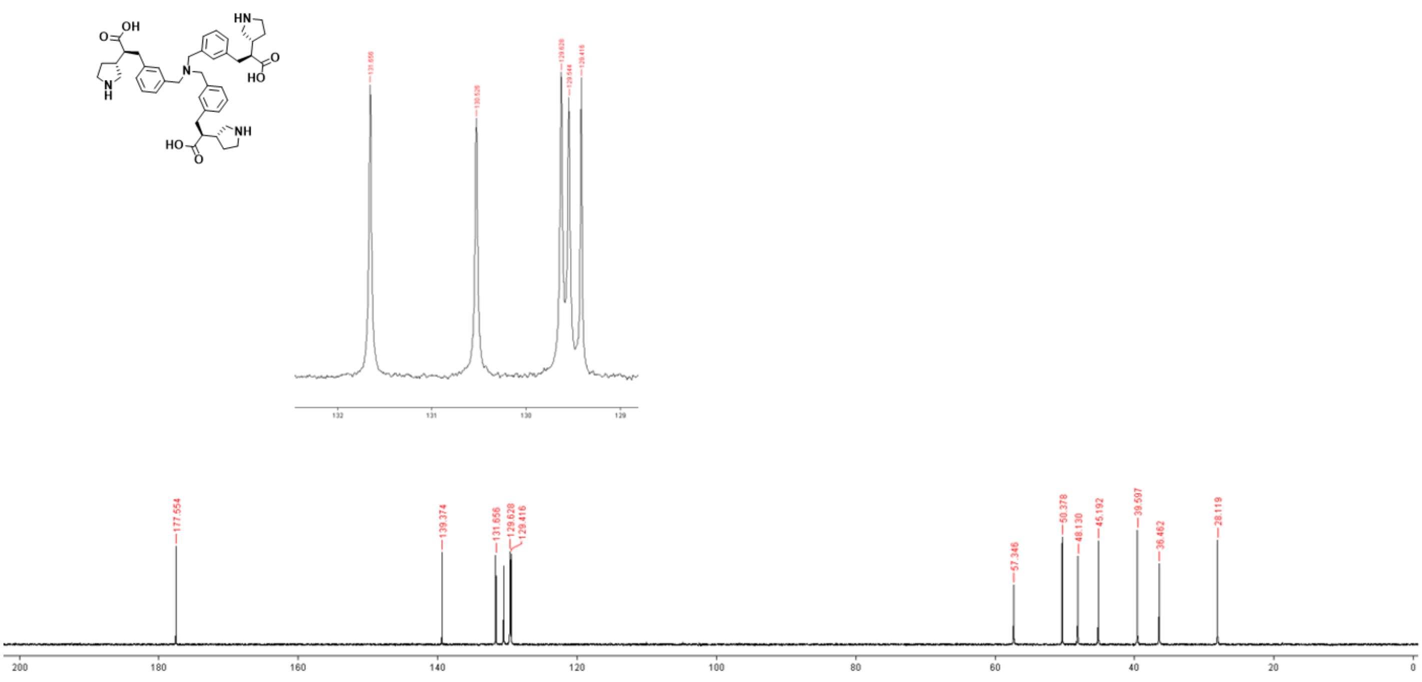

Alcobendas AT

```
Operator:  Admin     Comment:  CHECK                                       ->
FILE NAME:  D:\Datos\20XX\23-12\15L\15L-E18295-081-22312042331.D
INJ. METHOD:  HIGHPH.M                                        Vial:  P1-C5
Method info:  Column XBridge C18 3.5um, 2.1 x 50mm; UV: 214 and 300; MS-ESI
              100-800    A: 10mM Ammonium Bicarbonate ph:9.0.    B: CH3CN
              Flow Rate: 1.2ml/min; T* 50ºC;  Gradient mode: From 5  to 95% B
              in 1.5min. Hold 0.5min at 95%B.
```

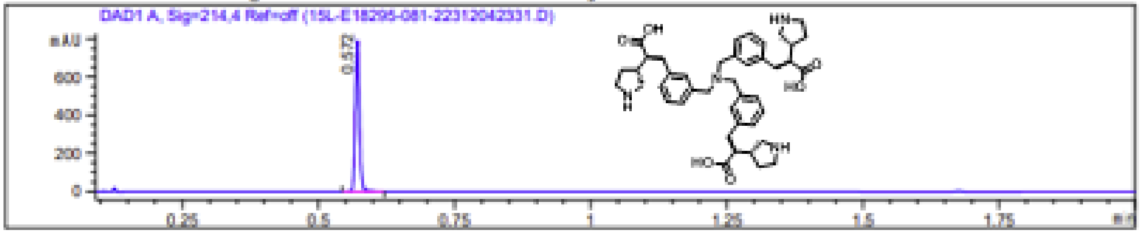

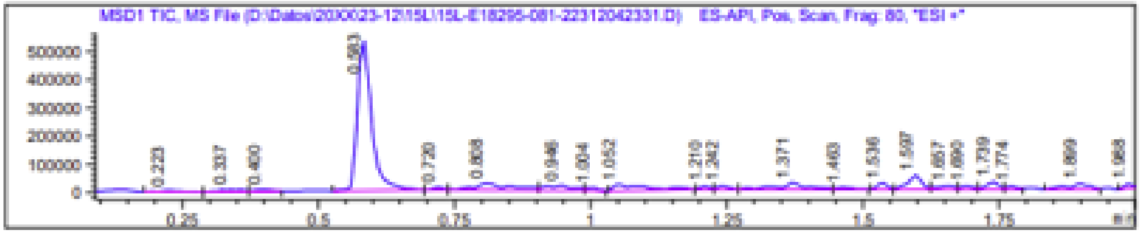

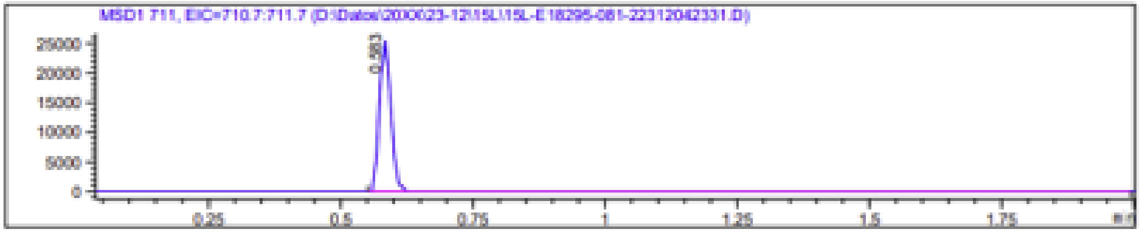

Area % Table of Signal DAD1 A, Sig=214,4

RET.T	Height	Area	Area%
0.572	754	409	100.00

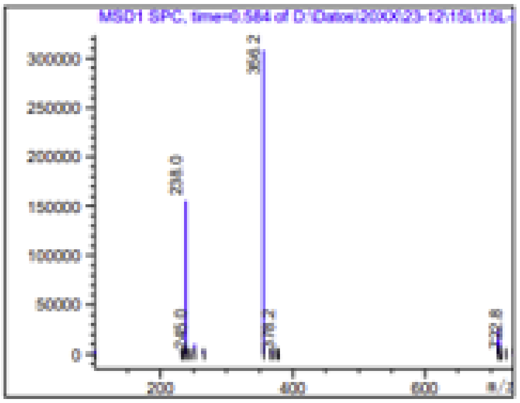

Target 1 : 710
UV RT T1 : 0.572
Purity T1 : 100.00

High Resolution Mass Spectrometry Report: LY3473329

Acquisition method: Column: X-Bridge C18 3.5µM 2.1x50mm. Mobile phase: A= 0.1% FA in water; B= 0.1% FA in CH_3CN. Run length: 2 min. Column temperature: 50ºC. Acquisition mode: ES+. Injection volume: 1µL

Time [min]	A [%]	B [%]	Flow [mL/min]	Max. Pressure Limit [bar]
0.00	95.00	5.00	1.200	1000.00
0.25	95.00	5.00	---	---
1.25	5.00	95.00	---	---
1.75	5.00	95.00	---	---
2.00	95.00	5.00	---	---

Ref. Standards from Agilent's Internal Reference Mass Kit (p/ G1969-85001)

- Purine (m/z 121.05087)
- HP-921 (m/z 922.00980)

**Sample**	
LSN	**3473329**
Molecular Formula	C42H54N4O6
Exact Mass	710.4043
Exact Mass (M+H)	711.4116
**Experimental Results**	
**Measured Exact Mass of $(M+H)^+$**	**711.4109**
ppm error with expected Exact Mass:	**-0.79**

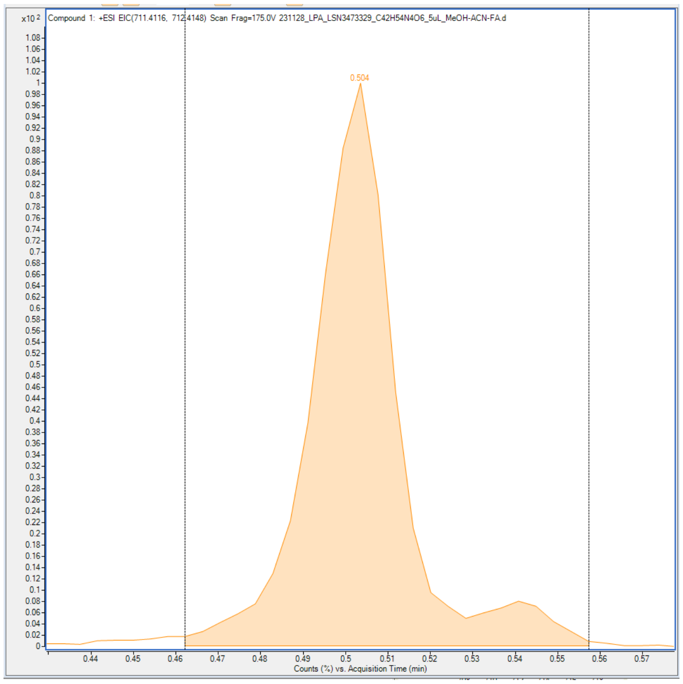
Compound 1: +ESI EIC(711.4116, 712.4148) Scan Frag=175.0V 231128_LPA_LSN3473329_C42H54N4O6_5uL_MeOH-ACN-FA.d
0.504
Counts (%) vs. Acquisition Time (min)

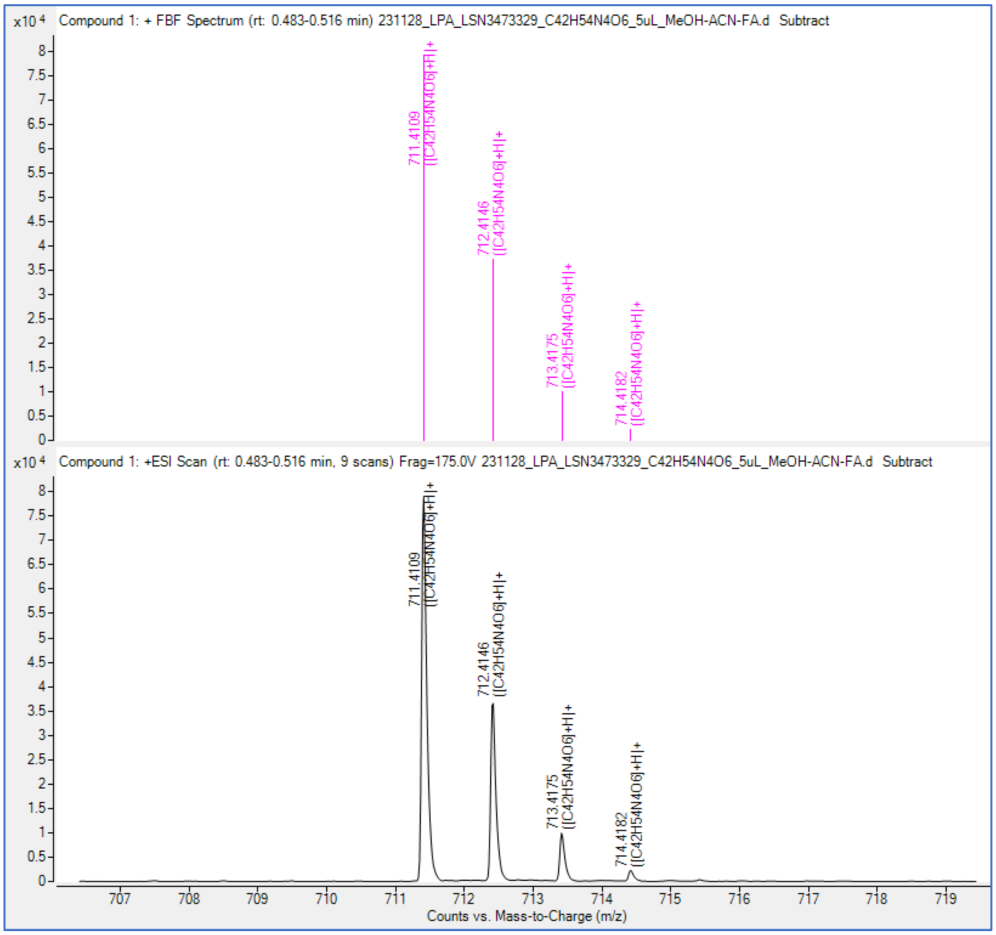
Compound 1: + FBF Spectrum (rt: 0.483-0.516 min) 231128_LPA_LSN3473329_C42H54N4O6_5uL_MeOH-ACN-FA.d Subtract
711.4109 ([C42H54N4O6]+H]+
712.4146 ([C42H54N4O6]+H]+
713.4175 ([C42H54N4O6]+H]+
714.4182 ([C42H54N4O6]+H]+
Compound 1: +ESI Scan (rt: 0.483-0.516 min, 9 scans) Frag=175.0V 231128_LPA_LSN3473329_C42H54N4O6_5uL_MeOH-ACN-FA.d Subtract
711.4109 ([C42H54N4O6]+H]+
712.4146 ([C42H54N4O6]+H]+
713.4175 ([C42H54N4O6]+H]+
714.4182 ([C42H54N4O6]+H]+
Counts vs. Mass-to-Charge (m/z)

LSN 3353871 (^{1}H NMR in D_2O)

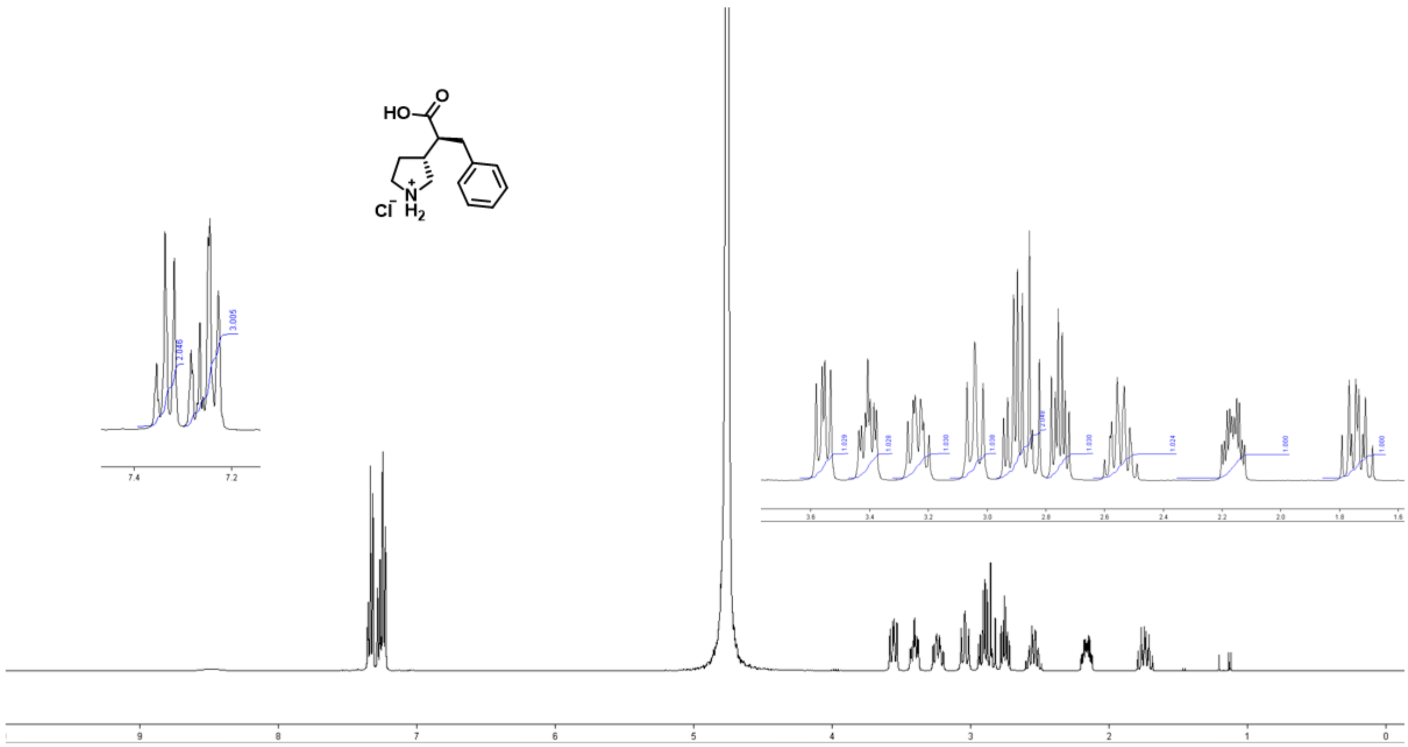

LSN 3353871 (^{13}C NMR in D_2O)

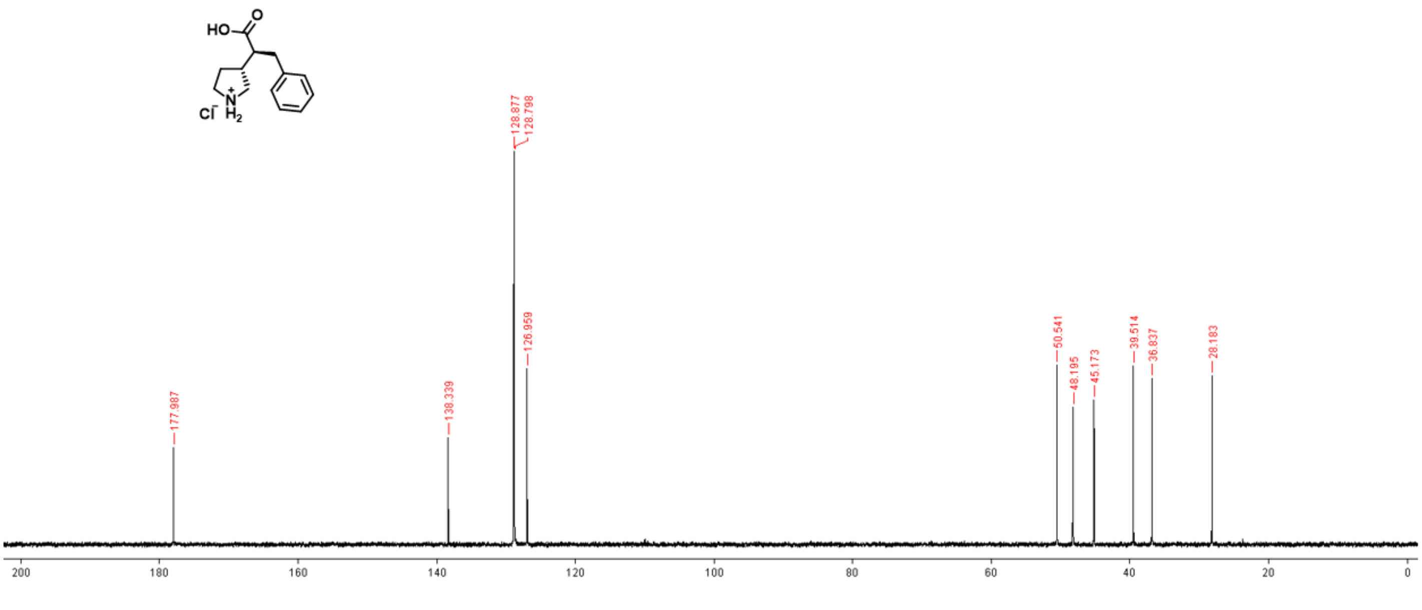

Alcobendas AT

Operator: 15L Comment: PRECIPITATE ->
FILE NAME: D:\DATOS\2015\15L\231115-15L1-19821.D
INJ. METHOD: C:\Chem32\1\METHODS\NEW_OA\MC_BASIC.M Vial: P1-E-08
Method info: Column XBridge C18 3.5um, 2.1 x 50mm; UV: 214 & 300; MS ESI(+/-) 150-700 A: 10mM Ammonium Bicarbonate ph:9.0. B: CH3CN Flow Rate: 1.0ml/min; Tª 50ºC; Gradient mode: From 10 to 100% B in 3 min. Hold 0.75min at 100%B.

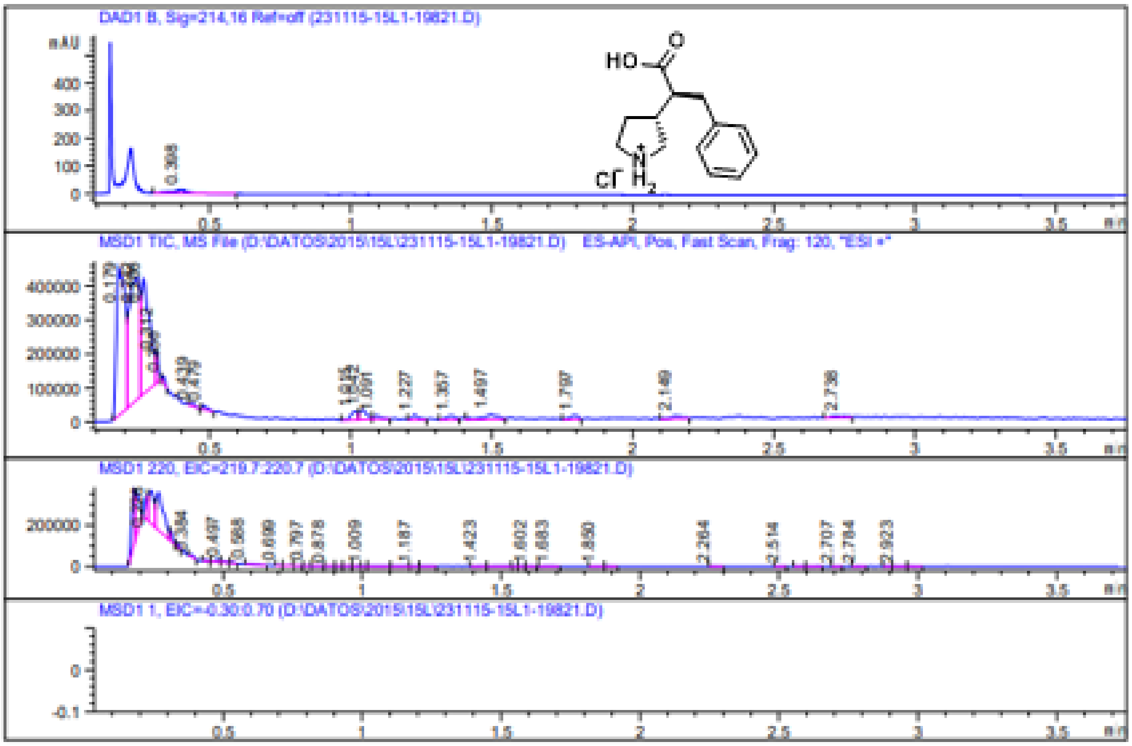

Area % Table of Signal DAD1 B, Sig=214,16

RET.T	Height	Area	Area%
0.398	13	50	100.00

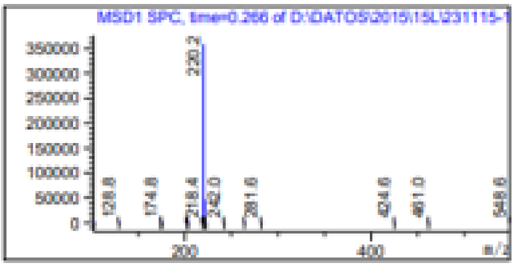

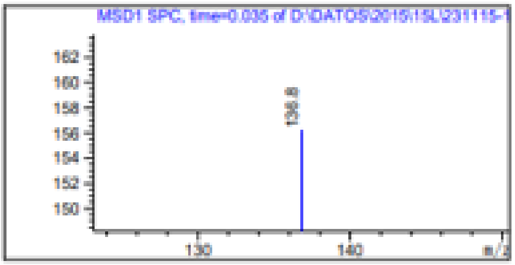

Target 1 : 219.00
UV RT T1 : 0.000
Purity T1 : 0.00
PosThr T1 : 181150

High Resolution Mass Spectrometry Report: LSN3353871

Acquisition method: Column: X-Bridge C18 3.5µM 2.1x50mm. Mobile phase: A= 0.1% FA in water; B= 0.1% FA in CH_3CN. Run length: 2 min. Column temperature: 50ºC. Acquisition mode: ES+. Injection volume: 1µL

Time [min]	A [%]	B [%]	Flow [mL/min]	Max. Pressure Limit [bar]
0.00	95.00	5.00	1.200	1000.00
0.25	95.00	5.00	---	---
1.25	5.00	95.00	---	---
1.75	5.00	95.00	---	---
2.00	95.00	5.00	---	---

Ref. Standards from Agilent's Internal Reference Mass Kit (p/ G1969-85001)

- Purine (m/z 121.05087)
- HP-921 (m/z 922.00980)

**Sample**	
LSN	**3353871**
Molecular Formula	C13H17NO2
Exact Mass	219.1259
Exact Mass (M+H)	220.1332
**Experimental Results**	
**Measured Exact Mass of $(M+H)^+$**	**220.1317**
ppm error with expected Exact Mass:	**-5.72**

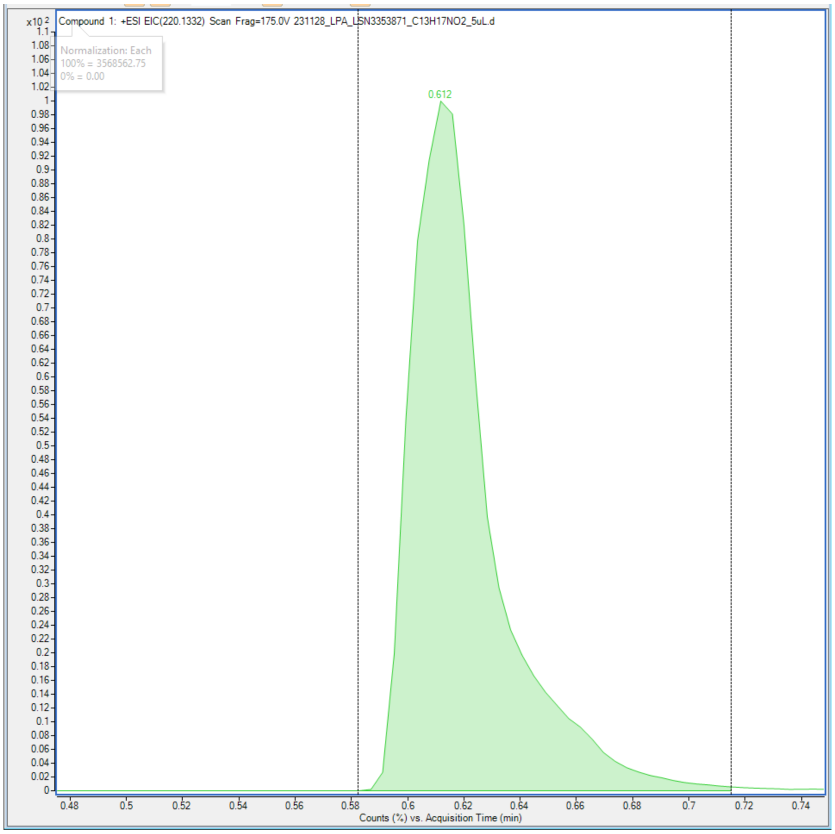
Compound 1: +ESI EIC(220.1332) Scan Frag=175.0V 231128_LPA_LSN3353871_C13H17NO2_5uL.d
Normalization: Each
100% = 3568562.75
0% = 0.00
0.612
Counts (%) vs. Acquisition Time (min)

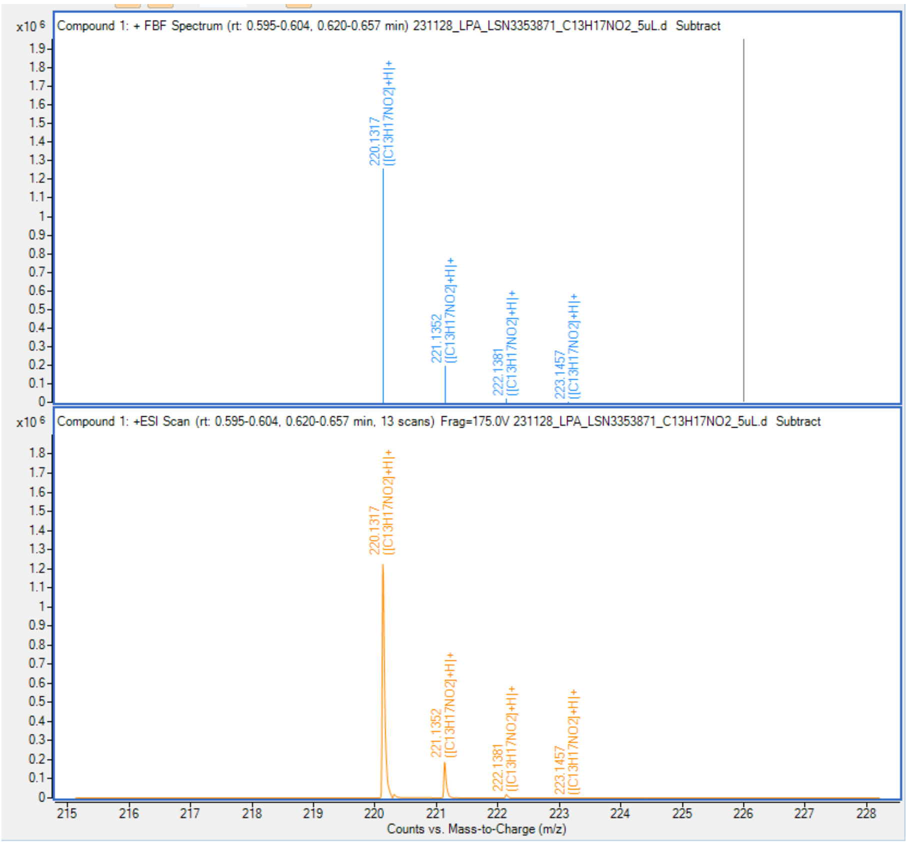
Compound 1: + FBF Spectrum (rt: 0.595-0.604, 0.620-0.657 min) 231128_LPA_LSN3353871_C13H17NO2_5uL.d Subtract
220.1317 ([C13H17NO2]+H]+
221.1352 ([C13H17NO2]+H]+
222.1381 ([C13H17NO2]+H]+
223.1457 ([C13H17NO2]+H]+
Compound 1: +ESI Scan (rt: 0.595-0.604, 0.620-0.657 min, 13 scans) Frag=175.0V 231128_LPA_LSN3353871_C13H17NO2_5uL.d Subtract
Counts vs. Mass-to-Charge (m/z)

LSN 3374443 (^{1}H NMR in D_2O + 1% DCl)

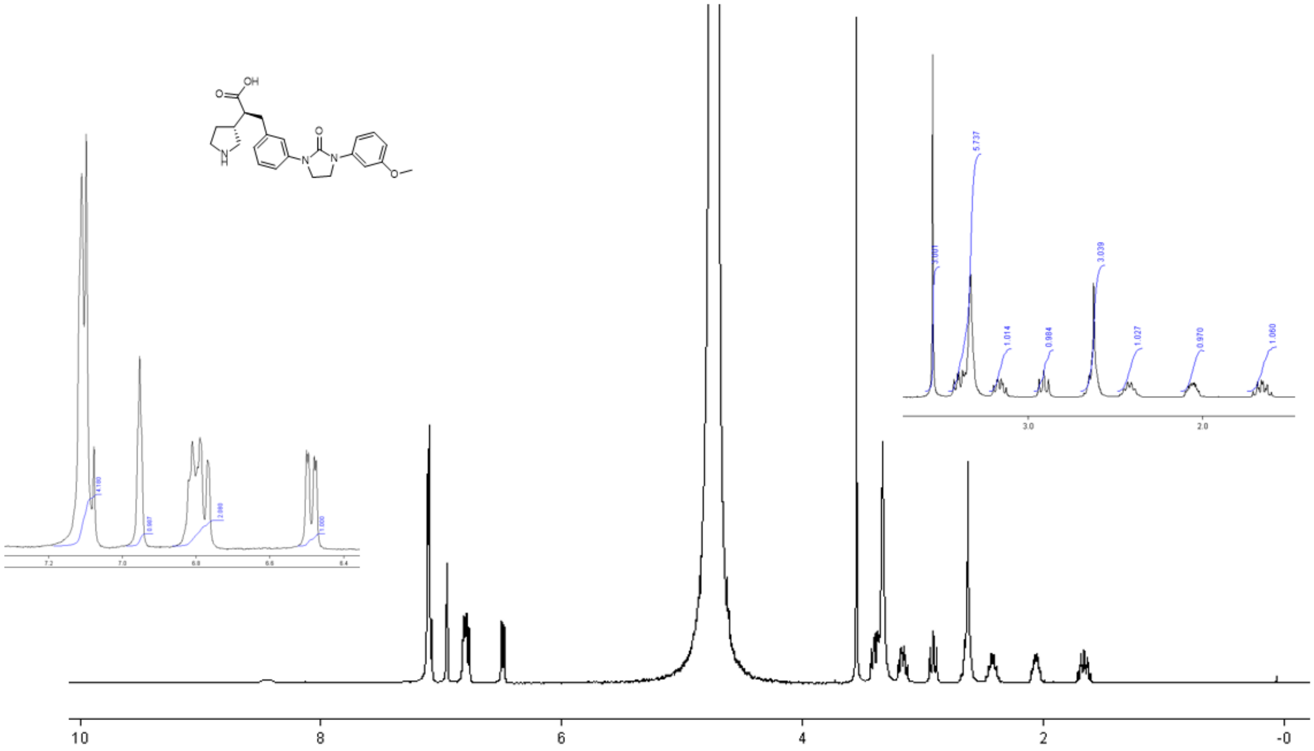

LSN 3374443 (^{13}C NMR in D_2O + 1% DCl))

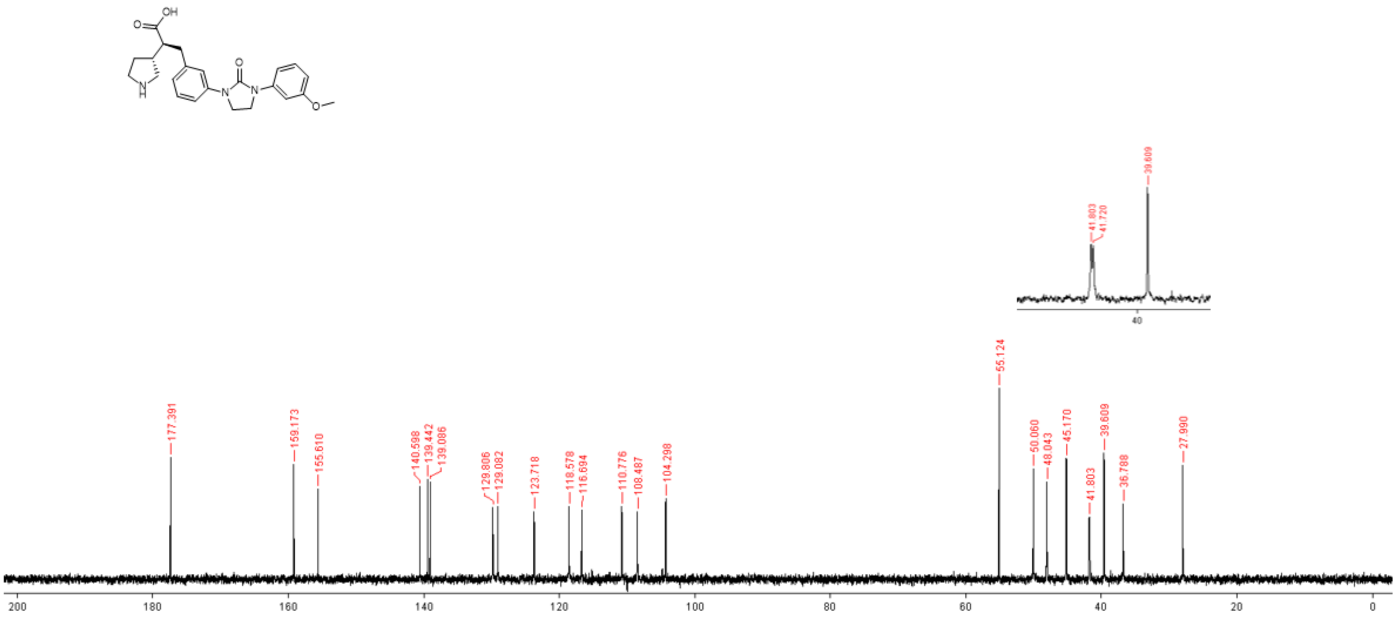

Alcobendas AT

Operator: Admin Comment: PURE ISCO REV ->
FILE NAME: D:\Datos\20XX\06-23\80K\80K-C00259-249-22306146068.D
INJ. METHOD: HIGHPH.M Vial: P1-D6
Method info: Column XBridge C18 3.5um, 2.1 x 50mm; UV: 214 and 300; MS-ESI 100-800 A: 10mM Ammonium Bicarbonate ph:9.0. B: CH3CN Flow Rate: 1.2ml/min; Tª 50ºC; Gradient mode: From 5 to 95% B in 1.5min. Hold 0.5min at 95%B.

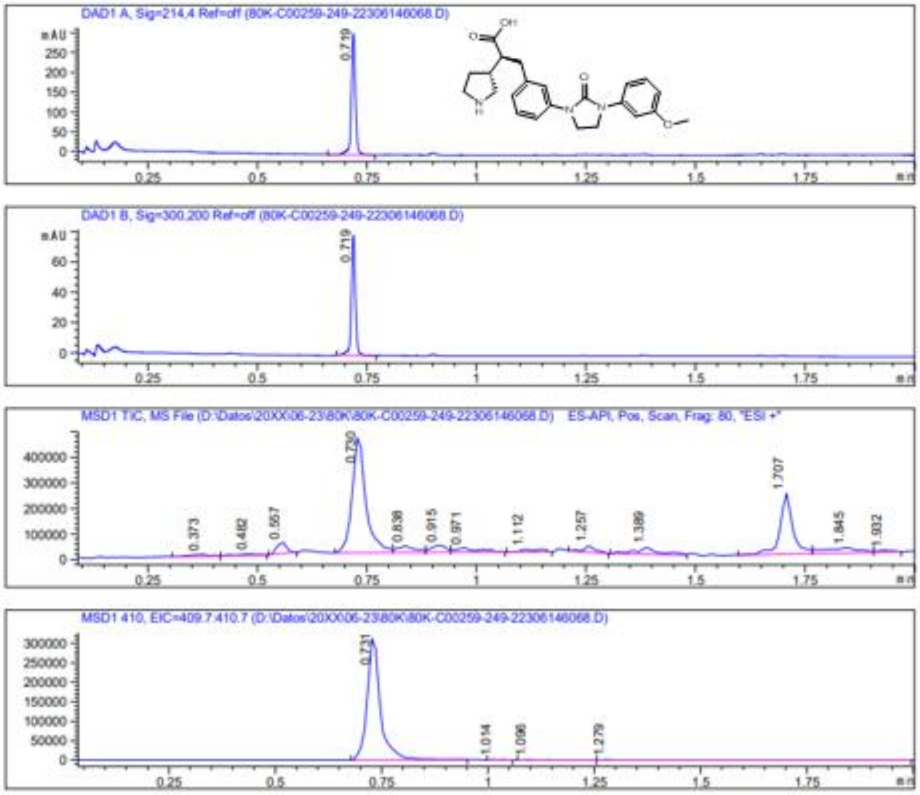

Area % Table of Signal DAD1 A, Sig=214,4

RET.T	Height	Area	Area%
0.719	291	196	100.00

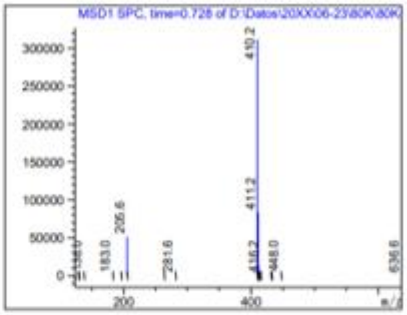

Target 1 : 409
UV RT T1 : 0.719
Purity T1 : 100.00
PosThr T1 : 318046

High Resolution Mass Spectrometry Report: LSN3374443

Acquisition method: Column: X-Bridge C18 3.5µM 2.1x50mm. Mobile phase: A= 0.1% FA in water; B= 0.1% FA in CH_3CN. Run length: 2 min. Column temperature: 50ºC. Acquisition mode: ES+. Injection volume: 1µL

Time [min]	A [%]	B [%]	Flow [mL/min]	Max. Pressure Limit [bar]
0.00	95.00	5.00	1.200	1000.00
0.25	95.00	5.00	---	---
1.25	5.00	95.00	---	---
1.75	5.00	95.00	---	---
2.00	95.00	5.00	---	---

Ref. Standards from Agilent's Internal Reference Mass Kit (p/ G1969-85001)

- Purine (m/z 121.05087)
- HP-921 (m/z 922.00980)
-

**Sample**	
LSN	**3374443**
Molecular Formula	C23H27N3O4
Exact Mass	409.2002
Exact Mass (M+H)	410.2074
**Experimental Results**	
**Measured Exact Mass of $(M+H)^+$**	**410.2053**
ppm error with expected Exact Mass:	**-4.82**

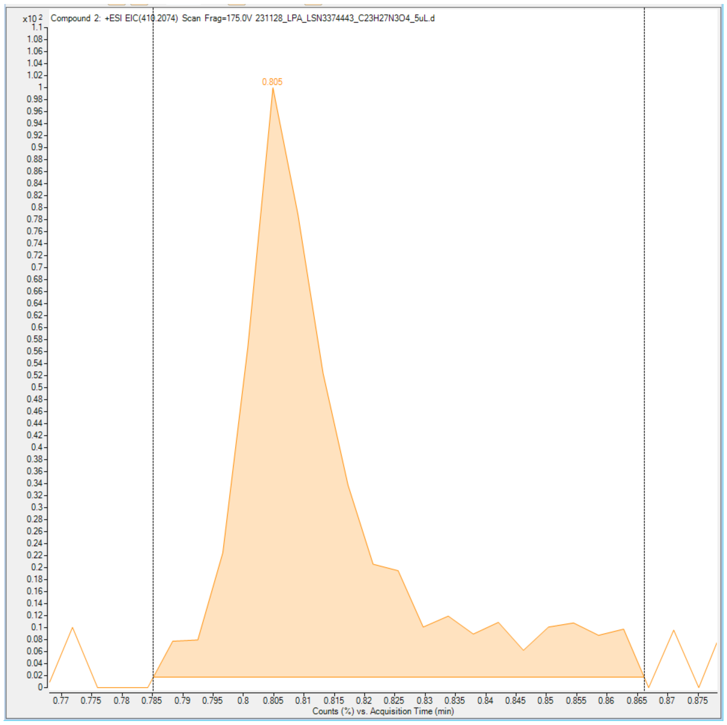
x10 2
Compound 2: +ESI EIC(410.2074) Scan Frag=175.0V 231128_LPA_LSN3374443_C23H27N3O4_5uL.d
0.805
Counts (%) vs. Acquisition Time (min)

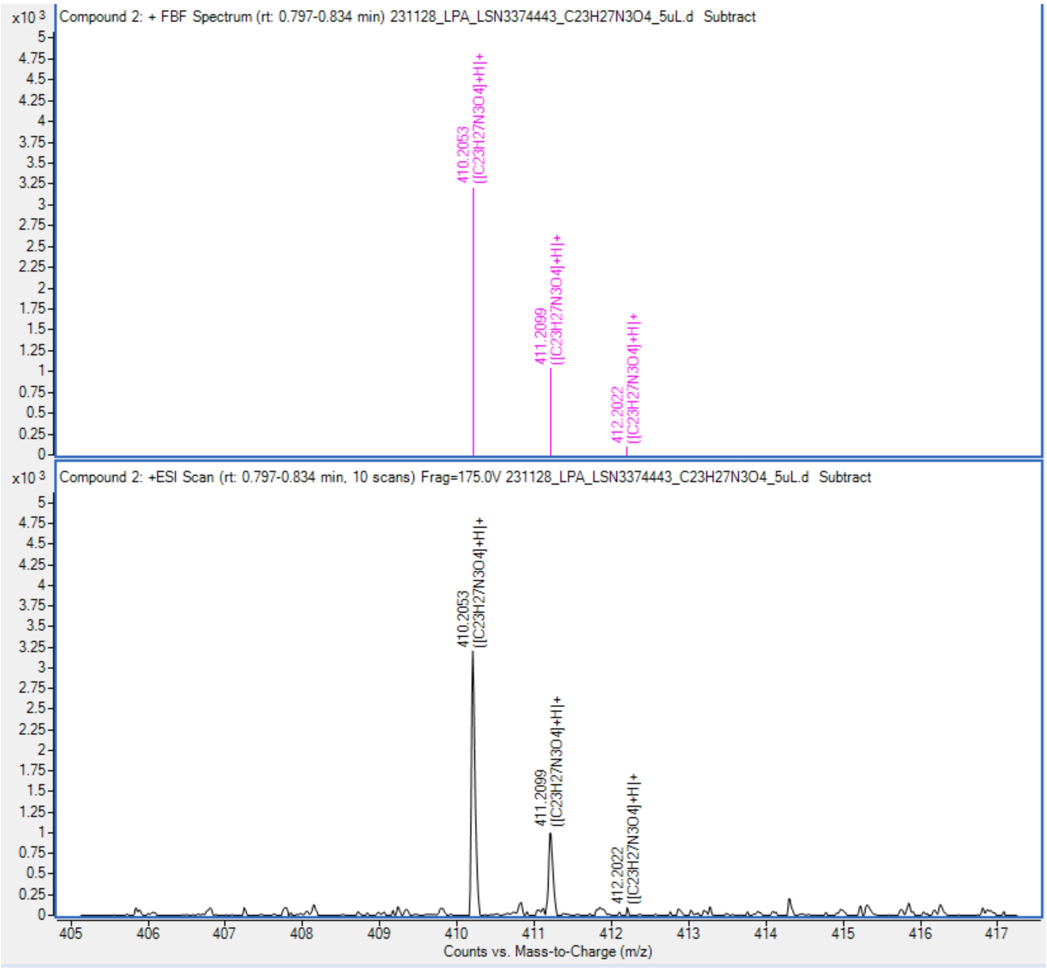
x10 3
Compound 2: + FBF Spectrum (rt: 0.797-0.834 min) 231128_LPA_LSN3374443_C23H27N3O4_5uL.d Subtract
410.2053 ([C23H27N3O4]+H]+
411.2099 ([C23H27N3O4]+H]+
412.2022 ([C23H27N3O4]+H]+
x10 3
Compound 2: +ESI Scan (rt: 0.797-0.834 min, 10 scans) Frag=175.0V 231128_LPA_LSN3374443_C23H27N3O4_5uL.d Subtract
410.2053 ([C23H27N3O4]+H]+
411.2099 ([C23H27N3O4]+H]+
412.2022 ([C23H27N3O4]+H]+
Counts vs. Mass-to-Charge (m/z)

TRQ41776 [3H]LSN3441732

Injection Date/Time: 02/10/2017 17:41:27   Injection Volume: 8 µL

Sample info: Active
Column: Inertsil ODS3 5 µm 250 x 4.6 mm
Column temperature: 22 °C
Eluent A: Water:trifluoroacetic acid (1000:1)
Eluent B: Acetonitrile:trifluoroacetic acid (1000:1)
Gradient T(min)/%B: 0/10, 20/40, 21/100, 31/100, 32/10, 35/10 @ 1 mL/min

Radiochemical Signal

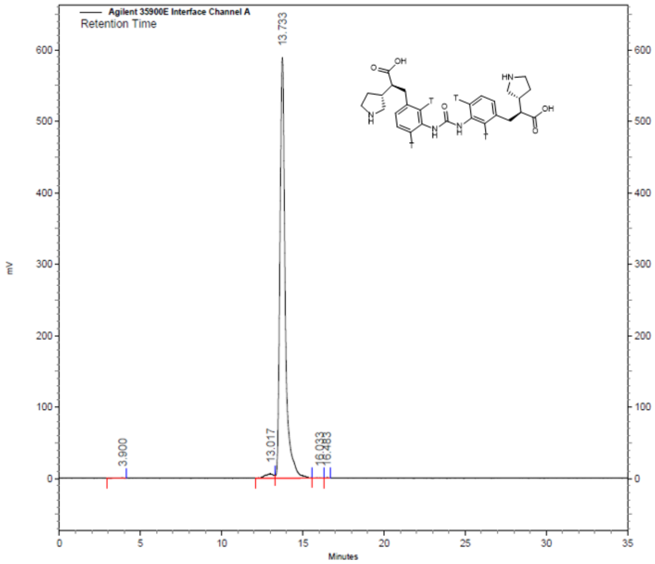

TRQ41776 [3H]LSN3441732

Agilent 35900E
Interface Channel A
Results

Name	Retention Time	Area	Area Percent
	3.900	2396238	0.2
	13.017	22328988	1.7
	13.733	1323533567	98.0
	16.033	1983292	0.1
	16.483	758097	0.1
Totals			
		1351000182	100.0

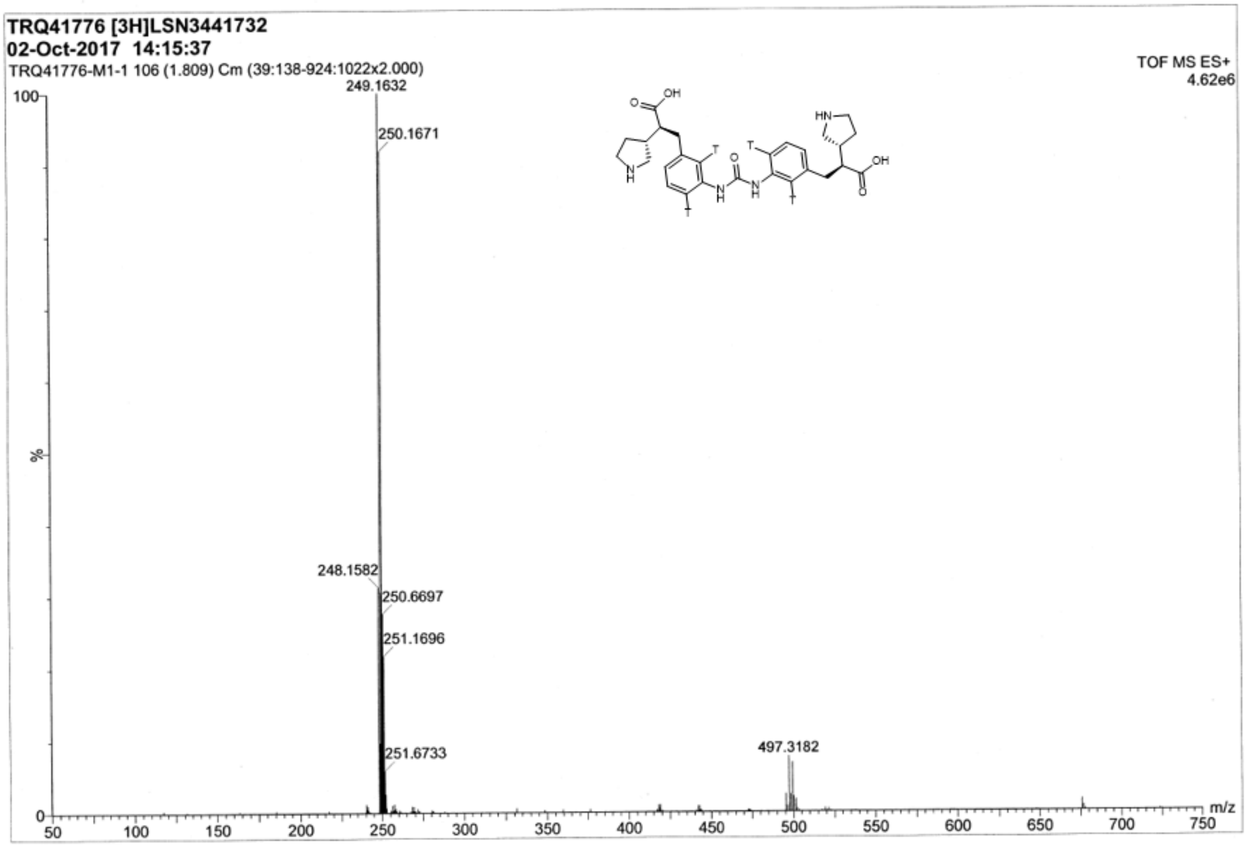

TRQ41776 [3H]LSN3441732
02-Oct-2017 14:15:37
TRQ41776-M1-1 106 (1.809) Cm (39:138-924:1022x2.000)
TOF MS ES+
4.62e6
249.1632
250.1671
248.1582
250.6697
251.1696
251.6733
497.3182
%
m/z

TRQ41776 [3H]LSN3441732

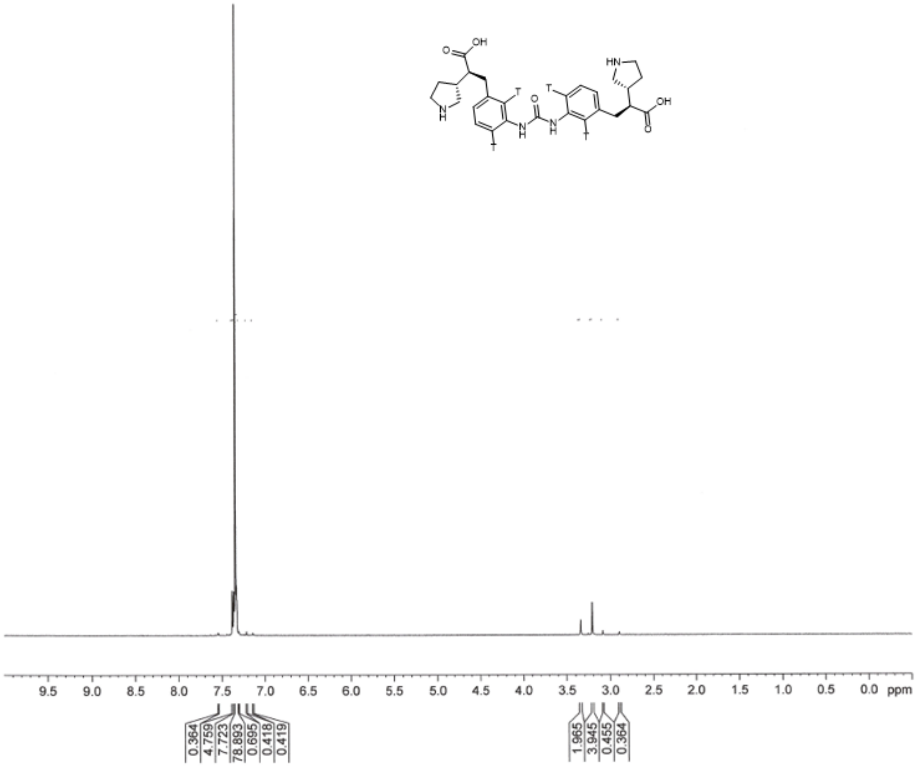

NAME	TRQ41776-N1-1.171004
EXPNO	2
PROCNO	1
Date_	20171004
Time	15.55
INSTRUM	av400
PROBHD	5 mm DUX 3H-1H
PULPROG	zgdc
TD	32000
SOLVENT	DMSO
NS	8448
DS	0
SWH	8278.146 Hz
FIDRES	0.258692 Hz
AQ	1.9328500 sec
RG	1625.5
DW	60.400 usec
DE	6.00 usec
TE	298.0 K
D1	5.00000000 sec
D11	0.03000000 sec
TD0	33
======== CHANNEL f1 ========	
NUC1	3H
P1	13.85 usec
PL1	-1.00 dB
PL1W	2.52982211 W
SFO1	426.7966930 MHz
======== CHANNEL f2 ========	
CPDPRG2	waltz16
NUC2	1H
PCPD2	101.00 usec
PL2	-0.90 dB
PL12	18.00 dB
PL2W	8.96185684 W
PL12W	0.11545108 W
SFO2	400.1324710 MHz
SI	32768
SF	426.7945590 MHz
WDW	EM
SSB	0
LB	0.30 Hz
GB	0
PC	1.00

Table with abbreviations

BOC	*tert*-butyloxycarbonyl
C	Celsius
Ci	Curie
d	doublet
DMSO	Dimethylsulfoxide
DAD	diode-array detection
ESI	Electrospray ionization
EtOAc	Ethyl Acetate
g	gram
h	hour
HPLC	High Performance Liquid Chromatography
HRMS	High-resolution mass spectrometry
DART-TOF	High-resolution mass spectrometry direct analysis in real time-time of flight
de	Diastereomeric excesses
ee	Enantiomerically excesses
IPA	Isopropylalcohol
LC	Liquid chromatography
LCMS	Liquid chromatography mass spectrometry
m	multiplet
M	Molar
MHz	Megahertz
mL	milliliter
mmol	millimol
MS	mass spectrometry
MTBE	methyl *tert*-butyl ether
N	Normal
NMR	Nuclear magnetic resonance
ppm	parts per million

psi	Pounds per square inch
ºC	Degree Celsius
QTOF	Quadrupole Time-of-Flight
s	singlet
SFC	Supercritical Fluid Chromatography
t	triplet
t_R	Retention time
TFA	Trifluoroacetic acid
THF	Tetrahydrofuran
UV	Ultraviolet
