# Supplementary material for: Discovery of potent small-molecule inhibitors of lipoprotein(a) formation
Source: Nature. 2024 May 8;629(8013):945–50. doi: 10.1038/s41586-024-07387-z (PMC11111404; doi:10.1038/s41586-024-07387-z)
Supplement: Supplementary file 2 — Reporting Summary [file 41586_2024_7387_MOESM2_ESM.pdf]

nature portfolio

Corresponding author(s): Laura F Michael, PhD

Last updated by author(s): Apr 16, 2024

Reporting Summary

Nature Portfolio wishes to improve the reproducibility of the work that we publish. This form provides structure for consistency and transparency in reporting. For further information on Nature Portfolio policies, see our Editorial Policies and the Editorial Policy Checklist.

Statistics

For all statistical analyses, confirm that the following items are present in the figure legend, table legend, main text, or Methods section.

n/a	Confirmed	
☐	☒	The exact sample size (*n*) for each experimental group/condition, given as a discrete number and unit of measurement
☐	☒	A statement on whether measurements were taken from distinct samples or whether the same sample was measured repeatedly
☐	☒	The statistical test(s) used AND whether they are one- or two-sided<br>*Only common tests should be described solely by name; describe more complex techniques in the Methods section.*
☒	☐	A description of all covariates tested
☒	☐	A description of any assumptions or corrections, such as tests of normality and adjustment for multiple comparisons
☐	☒	A full description of the statistical parameters including central tendency (e.g. means) or other basic estimates (e.g. regression coefficient) AND variation (e.g. standard deviation) or associated estimates of uncertainty (e.g. confidence intervals)
☐	☒	For null hypothesis testing, the test statistic (e.g. *F*, *t*, *r*) with confidence intervals, effect sizes, degrees of freedom and *P* value noted<br>*Give P values as exact values whenever suitable.*
☒	☐	For Bayesian analysis, information on the choice of priors and Markov chain Monte Carlo settings
☒	☐	For hierarchical and complex designs, identification of the appropriate level for tests and full reporting of outcomes
☒	☐	Estimates of effect sizes (e.g. Cohen's *d*, Pearson's *r*), indicating how they were calculated

Our web collection on statistics for biologists contains articles on many of the points above.

Software and code

Policy information about availability of computer code

Data collection	The crystallography diffraction data were indexed and integrated using MOSFLM 7.0.5 and merged and scaled with Scala 3.3 and Truncate 6.5 from the CCP4 6.5 suite. The coordinates were refined using Refmac 5.8 (CCP4). Model building was performed with Coot 0.8 (CCP4) and final structure validation with MolProbity 4.02 and CCP4 validation tools.
Data analysis	All in vitro and in vivo data were analyzed in GraphPad Prism version 9.5.1. For Isothermal Calorimetry, MicroCal PEAQ-ITC Analysis Software v.0.9. Non-human primate data were analyzed by repeated measures analysis of variance (ANOVA) in SAS 9.4. Statistical significance for Quantitative PCR was evaluated using JMP 16.1.0 software. The non-compartmental plasma PK parameters were calculated using Watson (version 7.5). Structural data images shown were generated with Molecular Operating Environment (MOE), 2022.02.

For manuscripts utilizing custom algorithms or software that are central to the research but not yet described in published literature, software must be made available to editors and reviewers. We strongly encourage code deposition in a community repository (e.g. GitHub). See the Nature Portfolio guidelines for submitting code & software for further information.

Data

Policy information about availability of data

All manuscripts must include a data availability statement. This statement should provide the following information, where applicable:

- Accession codes, unique identifiers, or web links for publicly available datasets
- A description of any restrictions on data availability
- For clinical datasets or third party data, please ensure that the statement adheres to our policy

All data supporting the in vivo findings are available within this article and in the extended data. The coordinates and structure factor files have been deposited in the Worldwide Protein Data Bank (https://www.wwpdb.org/) under the following accession numbers: 8TCE (KIV8 + LSN3353871), 8V9B (KIV7 + LSN3441732), and 8V8Z (KIV8 + LY3473329).

Research involving human participants, their data, or biological material

Policy information about studies with human participants or human data. See also policy information about sex, gender (identity/presentation), and sexual orientation and race, ethnicity and racism.

Reporting on sex and gender	Not applicable to this study
Reporting on race, ethnicity, or other socially relevant groupings	Not applicable to this study
Population characteristics	Not applicable to this study
Recruitment	Not applicable to this study
Ethics oversight	Not applicable to this study

Note that full information on the approval of the study protocol must also be provided in the manuscript.

Field-specific reporting

Please select the one below that is the best fit for your research. If you are not sure, read the appropriate sections before making your selection.

☒ Life sciences ☐ Behavioural & social sciences ☐ Ecological, evolutionary & environmental sciences

For a reference copy of the document with all sections, see nature.com/documents/nr-reporting-summary-flat.pdf

Life sciences study design

All studies must disclose on these points even when the disclosure is negative.

Sample size	Sample sizes were powered to detect significant Lp(a) lowering while ensuring no more animals were studied than necessary
Data exclusions	No data were excluded
Replication	Replicates are noted in the Figure legends
Randomization	Mice and cynomolgus monkeys were randomized to treatment groups by body weight and baseline plasma Lp(a) concentration using block randomized allocation tool (BRAT). Rats were randomized to treatment groups by baseline plasma plasminogen concentration and activity using BRAT.
Blinding	The investigator measuring Lp(a) efficacy in mouse and monkey pharmacology studies was blinded to group allocation.

Reporting for specific materials, systems and methods

We require information from authors about some types of materials, experimental systems and methods used in many studies. Here, indicate whether each material, system or method listed is relevant to your study. If you are not sure if a list item applies to your research, read the appropriate section before selecting a response.

Materials & experimental systems

n/a	Involved in the study	
☐	☒	Antibodies
☐	☒	Eukaryotic cell lines
☒	☐	Palaeontology and archaeology
☐	☒	Animals and other organisms
☒	☐	Clinical data
☒	☐	Dual use research of concern
☒	☐	Plants

Methods

n/a	Involved in the study	
☒	☐	ChIP-seq
☒	☐	Flow cytometry
☒	☐	MRI-based neuroimaging

Antibodies

Antibodies used	Goat anti-Lp(a) antibody (Abcam ab31675) was diluted 1:12500 in Hepes-buffered saline to create capture plates. HRP-conjugated goat anti-apoB antibody (Abcam ab27622) was diluted 1:3000 for detection.
Validation	As reported by the manufacturer, ab31675 recognizes lipoprotein a: 100%, Plasminogen: 0%, LDL: 0%, Apo B: 0%, and ab27622 recognizes Apolipoprotein B (Abcam, Cambridge, UK).

Eukaryotic cell lines

Policy information about cell lines and Sex and Gender in Research

Cell line source(s)	HepG2 (HB-8065, ATCC) HEK293 (CRL-1573, ATCC)
Authentication	None were authenticated
Mycoplasma contamination	Mycoplasma presence was not routinely evaluated
Commonly misidentified lines (See ICLAC register)	None of the commonly misidentified cell lines were used in this study

Animals and other research organisms

Policy information about studies involving animals; ARRIVE guidelines recommended for reporting animal research, and Sex and Gender in Research

Laboratory animals	Male Sprague Dawley rats, body weights ranged from 280-330 g, and age ranged from 8-9 weeks old.<br><br>Lp(a) transgenic mouse studies were conducted in female Lp(a) mice (B6.SJL-Tg(APOB)1102Sgy Tg(Alb-LPA)32Arte, Taconic, Rensselear, NY) ranging in age from 7 to 17 months old in each study.<br>Female or male cynomolgus monkey studies were conducted in subjects of unspecified ages with body weights ranging from 2-5 kg.
Wild animals	No wild animals were used in the study
Reporting on sex	Male and female as noted in Methods and figure legends
Field-collected samples	No field collected samples were used in the study
Ethics oversight	All procedures were conducted in compliance with the Animal Welfare Act, the Guide for the Care and Use of Laboratory Animals, and the Office of Laboratory Animal Welfare (Covance Laboratories Inc, Greenfield, IN, USA for cynomolgus monkey studies and Eli Lilly and Company, Indianapolis, IN, USA for mouse and rat studies).

Note that full information on the approval of the study protocol must also be provided in the manuscript.

Plants

Seed stocks	Not applicable to this study
Novel plant genotypes	Not applicable to this study
Authentication	Not applicable to this study
