# Supplementary material for: Discovery of potent small-molecule inhibitors of lipoprotein(a) formation
Source: Nature. 2024 May 8;629(8013):945–50. doi: 10.1038/s41586-024-07387-z (PMC11111404; doi:10.1038/s41586-024-07387-z)
Supplement: Supplementary file 3 — This table presents the crystallography data collection and refinement statistics. [file 41586_2024_7387_MOESM3_ESM.pdf]

Supplementary Information for

Discovery of potent small-molecule inhibitors of lipoprotein(a) formation

Supplementary Table S1

Crystallography data collection and refinement statistics

	3353871<br>8TCE	3441732<br>8V9B	3473329<br>8V8Z
**Data collection**			
Space group	$P2_1$	$P3_1$	$P2_12_12_1$
Cell dimensions			
a, *b*, *c* (Å)	37.6, 57.57, 38.31	49.27, 49.27, 63.82	37.76, 75.18, 93.31
α, β, γ (°)	90, 94.7, 90	90, 90, 120	90, 90, 90
Resolution (Å)	1.07 (1.07-1.1)	1.19 (1.19-1.25)	2.01 (2.01-2.12)
R_{merge}	0.068 (0.61)	0.112 (0.638)	0.102 (0.499)
$I / \sigma I$	7.9 (1.8)	5.7 (1.2)	6.7 (1.5)
Completeness (%)	91.1 (70.7)	94.3 (94.3)	99.3 (98.7)
Redundancy	3.5 (2.9)	5.8 (5.3)	7.3 (7.5)
**Refinement**			
Resolution (Å)	1.07	1.19	2.01
Number of reflections	61,502	55,431	18,167
R_{work} / R_{free}	0.168/0.187	0.141/0.175	0.217/0.260
Number of atoms			
Protein	1,505	1,385	1,993
Ligand	32	70	107
Water	200	215	91
B-factors			
$K_{IV}7$	-	9.54	-
$K_{IV}8$	9.45	-	29.10
3353871	16.76	-	-
3441732	-	13.92	-
3473329	-	-	31.38
Water	18.62	21.39	28.50
R.m.s deviations			
Bond lengths (Å)	0.007	0.011	0.008
Bond angles (°)	1.303	1.450	1.199

K_{IV}, kringle type IV domain.
